# Tackling *N*‐Alkyl Imines with 3d Metal Catalysis: Highly Enantioselective Iron‐Catalyzed Synthesis of α‐Chiral Amines

**DOI:** 10.1002/anie.202006557

**Published:** 2020-06-25

**Authors:** Clemens K. Blasius, Niklas F. Heinrich, Vladislav Vasilenko, Lutz H. Gade

**Affiliations:** ^1^ Anorganisch-Chemisches Institut Universität Heidelberg Im Neuenheimer Feld 270 69120 Heidelberg Germany

**Keywords:** enantioselective catalysis, hydroboration, imine reduction, iron, *N*-alkyl amines

## Abstract

A readily activated iron alkyl precatalyst effectively catalyzes the highly enantioselective hydroboration of N‐alkyl imines. Employing a chiral bis(oxazolinylmethylidene)isoindoline pincer ligand, the asymmetric reduction of various acyclic N‐alkyl imines provided the corresponding α‐chiral amines in excellent yields and with up to >99 % ee. The applicability of this base metal catalytic system was further demonstrated with the synthesis of the pharmaceuticals Fendiline and Tecalcet.

## Graphical abstract

**Ironing imines**: The first iron‐catalyzed asymmetric reduction of *N*‐alkyl imines is reported. A molecularly defined iron complex catalyzes the highly enantioselective hydroboration of various *N*‐alkyl imines, providing access to the corresponding α‐chiral amines in high yields and stereoselectivities. The earth‐abundant metal catalyst was applied in the synthesis of the pharmaceuticals Fendiline and Tecalcet.

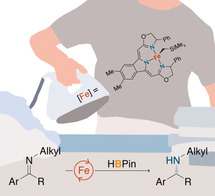

Optically pure *N*‐alkyl amines are essential structural motifs in numerous bioactive compounds, as exemplified by pharmaceuticals such as BMS‐394136,^1^ Sertraline,^2^ Methamphetamine,^3^ (*S*)‐Rivastigmine,^4^ and Cinacalcet^5^ (Figure 1).^6^ Of the various strategies for their enantioselective synthesis, the direct formation of α‐chiral amines via asymmetric reduction of prochiral imines is among the most useful and attractive synthetic routes to date.^7^ In view of the resulting highly basic amine products, however, catalyst deactivation processes limit the applicability of many catalytic systems to the conversion of “activated” substrates, such as *N*‐tosyl or *N*‐phosphinoyl imines, and render the efficient conversion of *N*‐alkyl imines particularly challenging.^8^

**Figure 1:**
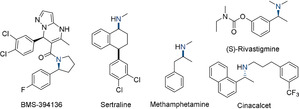
Selected α‐chiral amines in bioactive compounds and pharmaceuticals.

To address this issue, List et al. reported the use of chiral disulfonimide catalysts in combination with the Hantzsch ester in the presence of Boc_2_O for the synthesis of Boc‐protected, enantiomerically pure *N*‐alkyl amines.^9^ Subsequent studies on diazaphospholenes^10^ and phosphenium cations^11^ also demonstrated the applicability of pinacolborane as a reducing agent, albeit only with moderate stereoinduction except in reactions with cyclic imines.

Despite the early contributions by Buchwald and co‐workers on the titanium‐mediated asymmetric hydrogenation^12^ and hydrosilylation^13^ of imines, research efforts in transition metal catalysis mainly focused on the use of platinum metals, producing a variety of catalytic systems for the enantioselective hydrogenation of several prochiral imines.^7^, ^8^, ^14^ However, although the reduction of *N*‐aryl,^15^ *N*‐benzyl,15j, 16 and endocyclic *N*‐alkyl imines12a, 17 is nowadays well‐established, it was not until recently that an iridium complex was efficiently applied in the asymmetric hydrogenation of acyclic *N*‐methyl and *N*‐alkyl imines (up to 94 % *ee*).^18^

Given the current interest in replacing noble metals by earth‐abundant 3d metals, which generally operate via different mechanisms,^19^ recent studies have focused especially on the development of iron‐based catalysts.^20^ Despite the tremendous progress in the field of enantioselective ketone reduction, versatile methods for the iron‐catalyzed asymmetric reduction of imines remain scarce (Scheme 1).^21^ In 2010, Beller et al. reported a catalytic system for the enantioselective transfer hydrogenation of *N*‐phosphinoyl imines, employing an iron carbonyl hydride cluster and a tetradentate P_2_N_2_ ligand.^22^ One year later, the limitation to this electronically privileged substrate class was overcome and the method was extended to *N*‐aryl imines by exploiting cooperative effects between a chiral Brønsted acid and an iron carbonyl complex.^23^ In addition, Morris and co‐workers explored the application of amine(imine)diphosphine iron catalysts for the asymmetric transfer hydrogenation of *N*‐phosphinoyl imines.^24^ More recent methods still rely on the use of “activated” substrates for an efficient stereoinduction.19a, 25 However, to our knowledge, no iron catalyst has been developed to date which effects the enantioselective reduction of *N*‐alkyl imines.

**Scheme 1:**
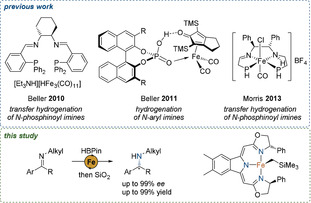
Recent examples for the iron‐catalyzed enantioselective reduction of privileged imines and our approach for the hydroboration of *N*‐alkyl imines.

Our previous studies established iron(II) alkyl complexes **1** supported by the bis(oxazolinylmethylidene)isoindoline (“boxmi”)^26^, ^27^ pincer ligand as suitable precatalysts for the enantioselective hydroboration of various functionalized ketones.^28^ Their high activity and excellent selectivity after initial conversion of the alkyl to the active hydrido species, combined with the observed tolerance towards ketones bearing amino groups, motivated us to expand this methodology to the reduction of *N*‐alkyl imines, and thus a class of compounds which hitherto eluded this type of transformation.

Reacting *N*‐methyl imine **2 a** in the presence of precatalyst ^H,Ph^boxmiFeCH_2_SiMe_3_ (**1 a**, 2.5 mol %) with pinacolborane resulted in 98 % conversion after 25 h, furnishing the corresponding α‐chiral amine with 95 % *ee* after workup (Table 1, entry 1). Interestingly, backbone methylation of the catalyst (**1 b**) led to a significant increase of the reaction rate and facilitated complete imine depletion without any impact on the enantiomeric excess (entry 2). In contrast, the introduction of phenyl groups at the oxazoline moieties was proven to be vital for sufficient enantiodiscrimination as demonstrated for the oxazoline substituents isopropyl, *tert*‐butyl, and benzyl (entries 4–6). In addition, solvent screening revealed the use of nonpolar reaction media as prerequisite for rapid conversions (entries 7–13, see the Supporting Information for details), allowing for further lowering of catalyst loadings and reaction temperatures for toluene/*n*‐hexane mixtures as solvents. Indeed, the hydroboration of *N*‐methyl imine **2 a** with 1.5 mol % catalyst loading of **1 b** was completed within 14 h when the reaction mixture was slowly warmed from −40 °C to room temperature, enhancing the observed stereodiscrimination to 96 % *ee* (entry 15).

**Table 1:** Screening of reaction conditions for the enantioselective reduction of *N*‐alkyl imines. 
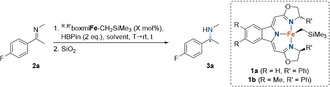

Entry	R	R′^[a]^	Solvent	Cat. [mol %]	*T* [°C]	*t* [h]	Conv. [%]^[b]^	*ee* [%]^[c]^
1	H	Ph	toluene	2.5	+26	25	98	95
2	Me	Ph	toluene	2.5	+26	25	>99	95
3	Ph	Ph	toluene	2.5	+26	25	99	95
4	H	*i*Pr	toluene	2.5	+26	25	71	n.d.
5	H	*t*Bu	toluene	2.5	+26	25	4	n.d.
6	Me	Bn	toluene	2.5	+26	25	75	n.d.
7	Me	Ph	MeCN	2.5	+26	16.7	<5	n.d.
8	Me	Ph	CH_2_Cl_2_	2.5	+26	16.7	<5	n.d.
9	Me	Ph	THF	2.5	+26	17.8	96	92
10	Me	Ph	Et_2_O	2.5	+26	16.7	95	89
11	Me	Ph	*n*‐hexane	2.5	+26	16.7	95	92
12	Me	Ph	t/h 1:11^[d]^	2.5	+26	4.3	98	94
13	Me	Ph	t/h 1:5^[d]^	2.5	+26	4.3	98	94
14	Me	Ph	t/h 1:5^[d]^	2.5	−40	14	>99	96
15	Me	Ph	t/h 1:5^[d]^	1.5	−40	14	>99	96
16	Me	Ph	t/h 1:5^[d]^	1.0	−40	14	97	76

[a] (*S*,*S*) enantiomers of all ligands were employed. [b] Determined by ^19^F NMR spectroscopy. [c] Determined by HPLC analysis after derivatization to the corresponding benzamide. [d] Solvent mixtures of toluene (t) and *n*‐hexane (h) (*v*:*v* given). [e] Identical results were obtained for isolated and in situ generated precatalysts; reactions were performed at 0.1 mmol scale.

With these optimized reaction conditions in hand, we subjected a variety of *N*‐methyl imines derived from acetophenone and related structures to our standard hydroboration protocol (Scheme 2). Generally, a strong dependence of the catalytic activity on the steric and electronic properties of the respective imine was observed, requiring varying catalyst loadings in the range of 0.5–3.0 mol % and reaction times of up to 56 h. From a practical point of view, Boc (*tert*‐butyloxycarbonyl) or Bz (benzoyl) protection subsequent to the reduction step was carried out routinely for facile isolation of the amine products and determination of enantiomeric excess.

**Scheme 2:**
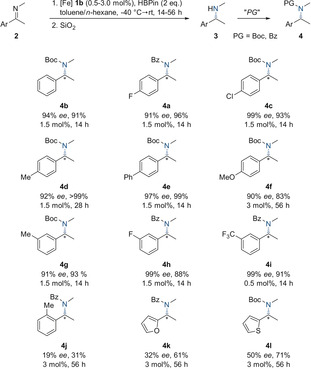
Substrate scope of the iron‐catalyzed reduction of *N*‐methyl imines. [a] Reactions were performed at 0.3 mmol scale in toluene/*n*‐hexane solvent mixtures (*v*:*v*=1:5). [b] Boc or Bz protection was carried out after reduction for facile isolation and characterization. [c] Determined by HPLC analysis. [d] Yield of isolated product. [e] Specific catalyst loadings and reaction times are given. [f] PG=protecting group.

Various *para*‐substituted *N*‐methyl imines stemming from acetophenone derivatives were efficiently reduced with very high enantiomeric excesses of up to 99 % under standard conditions (**2 a**–**f**). However, imines bearing electron‐donating *para*‐substituents required 3 mol % catalyst loading and extended reaction times as observed for the *para*‐methoxy derivative **2 f**. Furthermore, the hydroboration of imines with *meta*‐substituents proceeded without any interference (imines **2 g**–**i**). Regarding electronic effects, the *meta*‐CF_3_ group of imine **2 i** enabled full conversion within 14 h at catalyst loadings as low as 0.5 mol % and the corresponding α‐chiral amine was obtained with an excellent enantioselectivity of 99 % *ee*. In contrast, additional groups in the *ortho*‐position strongly hampered an efficient conversion as well as enantiodiscrimination (**2 j**). The comparatively electron‐rich heterocyclic furyl (**2 k**) or thienyl (**2 l**) derivatives were tolerated in the iron‐catalyzed hydroboration protocol, but displayed slow reaction rates and poor selectivity.

Remarkably, the observed enantioselectivities for the reduction of *N*‐methyl imines match or even exceed the results of iridium‐based^18^ systems or organocatalysts^9^ presented to date. The high selectivities and activities obtained for this iron‐catalyzed transformation encouraged us to investigate the asymmetric hydroboration of more complex substrates (Scheme 3). The mildly electron‐deficient polycyclic naphthalene derivative **2 m** was converted to amine **3 m** with low activity, but high enantioselectivity. As apparent from the reaction of imine **2 n**, which is derived from butyrophenone, α‐substitution is generally tolerated in the iron‐catalyzed hydroboration protocol. In contrast, the substitution pattern of the exocyclic derivative **2 o** appeared to be detrimental to an efficient stereodiscrimination. Conversely, endocyclic imines, commonly regarded as privileged substrates due to their isomerically pure C=N bond configuration, turned out to be Janus‐faced in the given context. On the one hand, imine **2 p** exhibiting a *Z*‐configured imine bond was hydroborated without any stereoinduction. The iron‐catalyzed reduction of endocyclic imine **2 q**, on the other hand, readily produced aryl pyrrolidine **3 q**, an essential structural fragment of the antiarrhythmic drug BMS‐394136,^1^ in 92 % *ee*. In addition, *N*‐alkyl imines with longer alkyl chains such as propyl (**2 r**) or hexyl (**2 s**) were reduced with only marginal decreases in yield and selectivity. Finally, the *N*‐benzyl‐substituted imine **2 t** as well as the thienyl analogue **2 u** were smoothly converted to the corresponding α‐chiral amines with 98 % *ee* and >99 % *ee*, respectively.

**Scheme 3:**
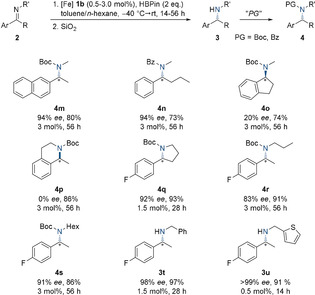
Substrate scope of the iron‐catalyzed reduction of *N*‐alkyl imines. [a] Reactions were performed at 0.3 mmol scale in toluene/*n*‐hexane solvent mixtures (*v*:*v*=1:5). [b] Boc or Bz protection was carried out after reduction for facile isolation and characterization. [c] Determined by HPLC analysis. [d] Yield of isolated product. [e] Specific catalyst loadings and reaction times are given. [f] PG=protecting group.

In order to probe the synthetic applicability of our catalytic system, we applied the iron‐based hydroboration protocol to the synthesis of two bioactive compounds (Scheme 4). In a first approach, we investigated the gram‐scale synthesis of (*R*)‐Fendiline, a calcium antagonist used in the treatment of coronary heart disease.^29^ In practical terms, precatalyst **1 b** was in situ generated from equimolar amounts of the iron dialkyl precursor (tmeda)Fe(CH_2_SiMe_3_)_2_ and the protioligand ^Me,Ph^boxmi‐H prior to the hydroboration step. Employing a catalyst loading of 0.5 mol %, 1.0 g of readily accessible imine **5** was reduced within 14 h, yielding the desired pharmaceutical in 98 % yield and 98 % *ee*.

**Scheme 4:**
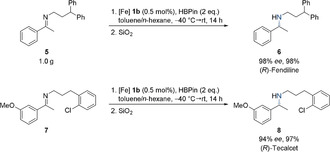
Synthesis of (*R*)‐Fendiline and (*R*)‐Tecalcet via iron‐catalyzed hydroboration.

In a similar way, imine **7** was enantioselectively hydroborated in the synthesis of Tecalcet, a calcimimetic compound for the treatment of hyperparathyroidism.^30^ Applying the same reaction conditions, α‐chiral secondary amine **8** was isolated in 97 % yield and with an enantiomeric excess of 94 % *ee*.

In conclusion, we have developed an iron‐based catalyst for the asymmetric reduction of *N*‐alkyl imines, overcoming the prevalent limitation to “activated” and *N*‐aryl imines in transition metal reductive catalysis. The earth‐abundant catalytic system matches current state‐of‐the‐art iridium hydrogenation catalysts and organocatalysts in terms of activity and selectivity. Importantly, the facile and in situ accessibility of the precatalyst employed highlights the general applicability of this hydroboration methodology for organic synthesis.

## Conflict of interest

The authors declare no conflict of interest.

## Supporting information

As a service to our authors and readers, this journal provides supporting information supplied by the authors. Such materials are peer reviewed and may be re‐organized for online delivery, but are not copy‐edited or typeset. Technical support issues arising from supporting information (other than missing files) should be addressed to the authors.

Supplementary
