# Supplementary material for: Tackling N‐Alkyl Imines with 3d Metal Catalysis: Highly Enantioselective Iron‐Catalyzed Synthesis of α‐Chiral Amines
Source: Angew Chem Int Ed Engl. 2020 Jun 25;59(37):15974–7. doi: 10.1002/anie.202006557 (PMC7539954; doi:10.1002/anie.202006557)
Supplement: Supplementary file 1 — Supplementary [file ANIE-59-15974-s001.pdf]

Angewandte Chemie
Eine Zeitschrift der Gesellschaft Deutscher Chemiker

Supporting Information

Tackling *N*-Alkyl Imines with 3d Metal Catalysis: Highly Enantioselective Iron-Catalyzed Synthesis of α-Chiral Amines

*Clemens K. Blasius, Niklas F. Heinrich, Vladislav Vasilenko, and Lutz H. Gade**

anie_202006557_sm_miscellaneous_information.pdf

Contents

1 General Informations **2**

2 Substrate Synthesis **3**

2.1 Synthetic Procedures 3

2.2 Analytical Data 4

3 Enantioselective Hydroboration of *N*-Alkyl-Imines **6**

3.1 Screening of Reaction Conditions 6

3.2 Synthetic Methods 7

3.3 Analytical Data of Isolated Products 9

3.4 Synthesis of Pharmaceuticals 18

4 Chromatographic Data **20**

5 NMR Data **43**

References **83**

1 General Informations

All manipulations, except when indicated otherwise, were carried out under exclusion of air and moisture using standard Schlenk and glovebox techniques. As inert gas, Argon 5.0, purchased from Messer Group GmbH, was used after drying over Granusic phosphorus pentoxide granulate. Solvents were dried over activated alumina columns using a solvent purification system (M. Braun SPS 800) or according to standard literature-known methods and stored in glass ampules under an argon atmosphere.[1] Toluene was distilled from sodium, *n*-pentane from sodium/potassium alloy, and tetrahydrofuran, benzene and *n*-hexane from potassium. The same procedures were used to dry the deuterated solvents. Degassed solvents and liquid substrates were obtained by three successive freeze-pump-thaw-cycles. NMR spectra were recorded on Bruker Avance (400 MHz, 600 MHz) instruments. Chemical shifts (δ) are reported in parts per million (ppm) and are referenced to residual proton solvent signals or carbon resonances.[2,3] CCl_3F (^{19}F) was used as external standard. Mass spectra were acquired on a Bruker ApexQe hybrid 9.4 T FT-ICR (ESI) or a JEOL AccuTOF GCx time-of-flight (EI) spectrometer at the mass spectrometry facility of the Institute of Organic Chemistry at the University of Heidelberg or on a Bruker micrOTOF II: ESI Mass Spectrometer at the Institute of Inorganic Chemistry. HPLC analyses were carried out on an Agilent 1200 Series chromatograph using chiral Daicel columns (AD-H, OD-H). Optical rotations were measured on a PerkinElmer Model 341 polarimeter and were exploited for an assignment of the absolute configuration by comparison with literature data. The catalysts $^{R,R'}$boxmiFeCH_2SiMe_3 (**1**) were synthesized according to literature procedures.[4] (*S*,*S*)-enantiomers of boxmi ligands were employed in all experiments. All substrates and other reagents were obtained from commercial suppliers and were used without further purification. All iron salts were purchased with a trace metal purity of 99.99 % or higher.

2 Substrate Synthesis

2.1 Synthetic Procedures

N-methyl imines **2a–m**,**o** were synthesised according to the following general procedure **GP1** adapted from literature:[5,6]

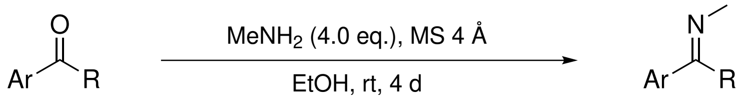

2

GP1. To an oven dried 100 ml Schlenk flask sealed with septum under argon and charged with 4.2 g of 4 Å molecular sieves 8.3 ml (66.6 mmol, 4.0 eq.) of a 33 w% solution of $MeNH_2$ in EtOH and the respective ketone (16.6 mmol, 1.0 eq.) were sequentially added. The reaction mixture was then stirred for at least 4 days at room temperature. In some cases higher temperatures, longer reaction times and/or a larger excess of $MeNH_2$ were required to reach full consumption of the ketone. After full conversion was indicated by NMR spectroscopy, the reaction mixture was filtered over Celite® and eluted with DCM. The crude product was freed from any volatiles under reduced pressure and purified by Kugelrohr distillation.

Imines **2n**,**r–u** were synthesised according to **GP2** adapted from literature:[7]

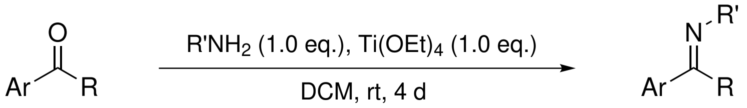

2

GP2. To a mixture of the respective ketone (16.6 mmol, 1.0 eq.) and amine (16.6 mmol, 1.0 eq.) in 60 mL DCM was added $Ti(OEt)_4$ (16.6 mmol, 1.0 eq.). The reaction mixture was then stirred at room temperature for at least 4 days. In some cases, higher temperatures or longer reaction times were required for sufficient conversion of the ketone. The reaction was quenched by adding aqueous KOH (15%) and the resulting suspension was filtered onto $NaSO_4$. The organic product was separated by filtration and extraction with DCM. Removal of the solvent under reduced pressure yielded the crude imine, which was purified by distillation or Kugelrohr distillation.

Imine **2q** was prepared according to literature procedures.[8] The synthesis of imines **2a–d**,**f**,**g**,**i**,**j**,**o**,[5] **2e**,**m**,[9] **2k**,[10] **2l**,[11] and **2t**[12] was reported previously.

2.2 Analytical Data

1-(3-Fluorophenyl)-*N*-methylethan-1-imine (2h)

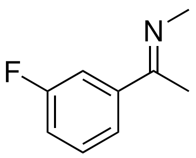

2h

Imine **2h** was synthesized according to **GP1** (4 d, room temperature). The crude product was purified by Kugelrohr distillation, affording the product as light yellow liquid (41%, 21:1 mixture of *E*/*Z* isomers). **^{1}H NMR (600.13 MHz, $CDCl_3$, 295 K):** major isomer: δ [ppm] = 7.52–7.51 (m, 1H), 7.48 (td, J = 10.4 Hz, J = 4.2 Hz, 1H), 7.33 (dt, J = 8.0 Hz, J = 5.9 Hz, 1H), 7.07 (dt, J = 8.3 Hz, J = 2.6 Hz, 1H), 3.35 (s, 3H), 2.22 (s, 3H). **^{13}C NMR (150.90 MHz, $CDCl_3$, 295 K):** major isomer: δ [ppm] = 165.9 (d, J = 2.7 Hz), 163.8 (d, J = 245.4 Hz), 143.6 (d, J = 7.0 Hz), 129.8 (d, J = 8.1 Hz), 122.2 (d, J = 2.8 Hz), 116.4 (d, J = 21.4 Hz), 113.5 (d, J = 22.6 Hz), 39.7 (s), 15.2 (s). **^{19}F NMR (376.23 MHz, $CDCl_3$, 295 K):** major isomer: δ [ppm] = −113.4. **HR-MS (EI$^+$):** $[M]^+$ = $C_9H_{10}NF^+$, calcd.: 151.07918, found: 151.07942.

N-Methyl-1-phenylbutan-1-imine (2n)

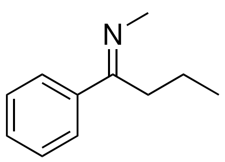

2n

Imine **2n** was synthesized according to **GP2** (4 d, room temperature). The crude product was purified by Kugelrohr distillation, affording the product as colorless liquid (22%, 2.5:1 mixture of *E*/*Z* isomers). **^{1}H NMR (600.13 MHz, $CDCl_3$, 295 K):** major isomer: δ [ppm] = 7.72–7.71 (m, 2H), 7.38–7.35 (m, 3H), 3.39 (s, 3H), 2.72–2.69 (m, 2H), 1.57–1.50 (m, 2H), 0.97 (t, J = 7.3 Hz). **^{13}C NMR (150.90 MHz, $CDCl_3$, 295 K):** major isomer: δ [ppm] = 170.9, 140.5, 129.4, 128.4, 126.8, 39.2, 30.3, 20.3, 14.4. **HR-MS (EI$^+$):** $[M]^+$ = $C_{11}H_{14}N^+$, calcd.: 160.11208, found: 160.11285.

1-(4-Fluorophenyl)-*N*-propylethan-1-imine (2r)

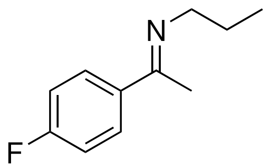

2r

Imine **2r** was synthesized according to **GP2** (1 w, room temperature). The crude product was purified by distillation, affording the product as colorless liquid (26%, 19:1 mixture of *E*/*Z* isomers). **^{1}H NMR (600.13 MHz, $CDCl_3$, 295 K):** major isomer: δ [ppm] = 7.78–7.75 (m, 2H), 7.05–7.02 (m, 2H), 3.42 (t, J = 7.1 Hz, 2H), 2.20 (m, 3H), 1.79–1.73 (m, 2H), 1.01 (t, J = 1.0 Hz, 3H). **^{13}C NMR (150.90 MHz, $CDCl_3$, 295 K):** major isomer: δ [ppm] = 163.7 (d, J = 248.5 Hz), 163.6 (s), 137.7 (d, J = 3.1 Hz), 128.5 (d, J = 8.4 Hz), 115.1 (d, J = 21.5 Hz), 54.0 (s), 24.3 (s), 15.4 (s), 12.3 (s) **^{19}F NMR (376.23 MHz, $CDCl_3$, 295 K):** major isomer: δ [ppm] = −112.6. **HR-MS (EI$^+$):** $[M]^+$ = $C_{11}H_{14}NF^+$, calcd.: 179.11048, found: 179.11110.

1-(4-Fluorophenyl)-*N*-hexylethan-1-imine (2s)

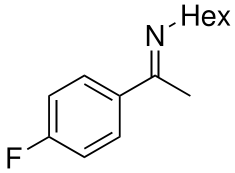

2s

Imine **2s** was synthesized according to **GP2** (4 d, room temperature). The crude product was purified by distillation, affording the product as colorless liquid (35%, 19:1 mixture of *E*/*Z* isomers). **^{1}H NMR (600.13 MHz, $CDCl_3$, 295 K):** major isomer: δ [ppm] = 7.77–7.75 (m, 2H), 7.05–7.02 (m, 2H), 3.44 (t, *J* = 7.2 Hz, 2H), 2.21 (s, 3H), 1.75–1.70 (m, 2H), 1.43–1.40 (m, 2H), 1.35–1.34 (m, 4H), 0.91–0.89 (m, 3H). **^{13}C NMR (150.90 MHz, $CDCl_3$, 295 K):** major isomer: δ [ppm] = 163.7 (d, *J* = 248.5 Hz), 163.7 (s), 137.7 (d, *J* = 3.1 Hz), 128.6 (d, *J* = 8.4 Hz), 115.1 (d, *J* = 21.5 Hz), 52.4 (s), 31.9 (s), 31.1 (s), 27.5 (s), 22.8 (s), 15.5 (s), 14.2 (s). **^{19}F NMR (376.23 MHz, $CDCl_3$, 295 K):** major isomer: δ [ppm] = −112.6. **HR-MS (EI$^+$):** $[M\text{-}H]^+$ = $C_{14}H_{19}NF^+$, calcd.: 220.14960, found: 220.15067.

(1-(4-fluorophenyl)-*N*-(thiophen-2-ylmethyl)ethan-1-imine (2u)

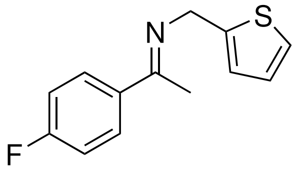

2u

Imine **2u** was synthesized according to **GP2** (1 w, room temperature). The crude product was purified by Kugelrohr distillation, affording the product as yellow solid (28%, 24:1 mixture of *E*/*Z* isomers). **^{1}H NMR (600.13 MHz, $CDCl_3$, 295 K):** major isomer: δ [ppm] = 7.88–7.86 (m, 2H), 7.24–7.22 (m, 1H), 7.08–7.05 (m, 2H), 7.00–6.99 (m, 1H), 4.88 (s, 2H), 2.33–2.32 (m, 3H). **^{13}C NMR (150.90 MHz, $CDCl_3$, 295 K):** major isomer: δ [ppm] = 165.2 (s), 164.1 (d, *J* = 249.4 Hz), 144.4 (s), 136.9 (d, *J* = 3.1 Hz), 128.9 (d, *J* = 8.5 Hz), 126.8 (s), 124.2 (s), 123.7 (s), 115.3 (d, *J* = 21.5 Hz), 51.2 (s), 15.9 (s). **^{19}F NMR (376.23 MHz, $CDCl_3$, 295 K):** major isomer: δ [ppm] = −111.7. **HR-MS (EI$^+$):** $[M]^+$ = $C_{13}H_{12}NFS^+$, calcd.: 233.06690, found: 233.06766.

3 Enantioselective Hydroboration of *N*-Alkyl-Imines

3.1 Screening of Reaction Conditions

Table 1: Screening of reaction conditions for the iron-catalyzed hydroboration of *N*-alkyl imines.

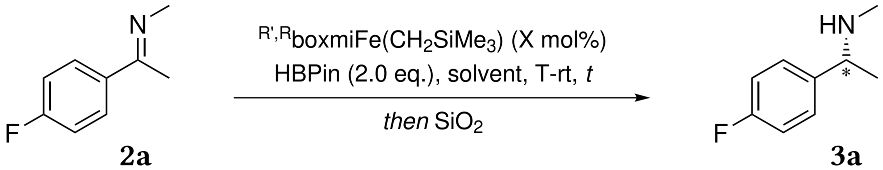

#[a]	R	R'	solvent[b]	mol%	T [°C]	t_1 [h]	conv.$_1$ [%][c]	t_2 [h]	conv.$_2$ [%][c]	*ee* [%][d]
1[e]	H	Ph	toluene	2.5	26	4.7	85	25	98	95
2[e]	Me	Ph	toluene	2.5	26	4.7	96	25	>99	95
3[e]	Ph	Ph	toluene	2.5	26	4.7	91	25	99	95
4[e]	H	*i*Pr	toluene	2.5	26	4.7	24	25	71	n.d.
5[e]	H	*t*Bu	toluene	2.5	26	4.7	2	25	4	n.d.
6[e]	Me	Bn	toluene	2.5	26	4.7	42	25	75	n.d.
7	Me	Ph	MeCN	2.5	26	5	0	16.7	0	n.d.
8	Me	Ph	CH_2Cl_2	2.5	26	2.5	0	16.7	0	n.d.
9	Me	Ph	thf	2.5	26	2.5	71	17.8	96	92
10	Me	Ph	Et_2O	2.5	26	2.5	91	16.7	95	89
11	Me	Ph	toluene	2.5	26	2.5	93	16.7	98	94
12[f]	Me	Ph	*n*-hexane	2.5	26	2.5	94	16.7	95	92
13	Me	Ph	t/h 1:11	2.5	26	0.6	91	4.3	98	94
14	Me	Ph	t/h 1:5	2.5	26	1.3	95	4.3	98	94
15	Me	Ph	t/h 1:1	2.5	26	1.3	91	4.3	95	94
16	Me	Ph	t/h 1:5	2.5	−40	n.d.	n.d.	14	99	96
17	Me	Ph	t/h 1:5	1.5	−40	n.d.	n.d.	14	99	96
18	Me	Ph	t/h 1:5	1.0	−40	n.d.	n.d.	14	97	76

[a] Reactions were performed at 0.1 mmol scale; [b] t/h indicates solvent mixtures of toluene and *n*-hexane (*v*:*v* = x:x); [c] Determined by ^{19}F NMR spectroscopy; [d] Determined by chiral HPLC analysis after derivatization into **3a-Bz** following **GP6**; [e] Precatalyst was synthesized *in situ* according to literature procedures; [f] Precatalyst not completely dissolved;

Note: The influence of the hydroboration reagent employed was investigated in a previous study on the stereoselective hydroboration of functionalized ketones.[4]

3.2 Synthetic Methods

Synthesis of Racemic Mixtures

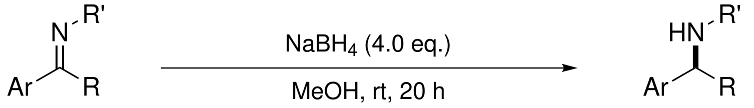

2 **3**

GP3. Under ambient conditions, imine **2** (130 µmol, 1.0 eq.) was dissolved in 1.0 ml MeOH and sodium borohydride (518 µmol, 4.0 eq.) was added in portions. After stirring the mixture for 12 h at room temperature, the reaction was quenched by adding aqueous saturated sodium chloride solution and the product was extracted with DCM. The combined organic phases were dried over sodium sulfate. Solvents were removed under reduced pressure to give the racemic amine, which was protected according to general procedure **GP5** or **GP6**, respectively.

Synthesis of α-Chiral Amines

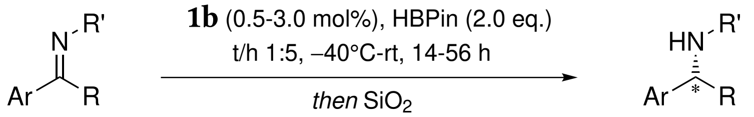

2 **3**

GP4. In a vial imine **2** (300 µmol, 1.0 eq.) and precatalyst **1b** (0.5, 1.5 or 3.0 mol %, respectively) were dissolved in 1.0 ml *n*-hexane and 200 µl toluene. The mixture was cooled to −40 °C and neat pinacolborane (76.8 mg, 600 µmol, 87.1 µl, 2.0 eq.) was added. After the respective reaction time (14 h, 28 h or 56 h), the reaction mixture was stirred for 1 h with an excess of silica, filtered using a DCM/NEt_3 mixture as eluent and concentrated under reduced pressure.

Boc Protection of α-Chiral Amines

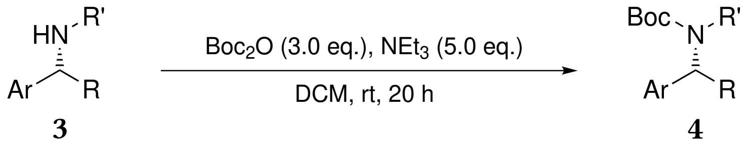

GP5. To a solution of crude amine **3** (1.0 eq.) in 2.0 ml DCM were added di-*tert*-butyl dicarbonate (3.0 eq.) and NEt_3 (5.0 eq.). After stirring the mixture for 12 h at room temperature, the reaction was quenched by the addition of aqueous saturated sodium bicarbonate solution and the product was extracted with DCM. The combined organic phases were dried over sodium sulfate and all volatiles were removed under reduced pressure. The crude product was purified by column chromatography using *n*-hexane/DCM as eluent to give the Boc-protected amine.

Bz Protection of α-Chiral Amines

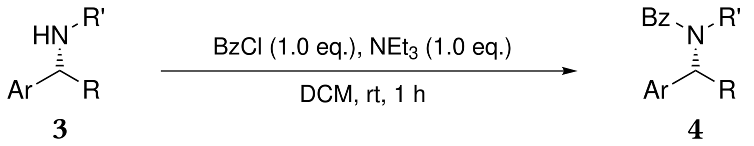

GP6. To a solution of amine **3** (1.0 eq.) in 2.0 ml DCM were added benzoyl chloride (1.0 eq.) and NEt_3 (1.0 eq.). After stirring the mixture for 1 h at room temperature, the reaction was treated with aqueous saturated sodium bicarbonate solution and the product was extracted with DCM. The combined organic phases were dried over sodium sulfate and all volatiles were removed under reduced pressure. The crude product was purified by column chromatography using *n*-hexane/DCM as eluent to give the benzoyl amide.

3.3 Analytical Data of Isolated Products

(*R*)-*N*-(1-(4-Fluorophenyl)-ethyl)-*N*-methylbenzamide (4a)

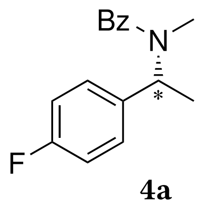

4a

Reduction of **2a** was carried out according to **GP4** (1.5 mol% **1b**, 14 h). Bz protection following **GP6** yielded **4a** as colorless solid in 96 % yield. **^{1}H NMR (600.13 MHz, CD_3CN, 333 K):** δ [ppm] = 7.45–7.36 (m, 7H), 7.12–7.10 (m, 2H), 5.58 (*br* s, 1H), 2.66 (s, 3H), 1.58 (d, *J* = 7.0 Hz, 3H). **^{13}C NMR (150.90 MHz, CD_3CN, 333 K):** δ [ppm] = 172.5 (s), 163.2 (d, *J* = 243.6 Hz), 138.8 (s), 138.2 (s), 130.4 (s), 130.1 (d, *J* = 8.5 Hz), 129.7 (s), 127.7 (s), 116.3 (d, *J* = 21.5 Hz), 17.1 (s). **^{19}F NMR (376.23 MHz, CD_3CN, 295 K):** δ [ppm] = −117.2, −117.4. **HR-MS (ESI$^+$):** $[M\text{-}H\text{+}Na]^+$ = $C_{16}H_{15}NFONa^+$, calcd.: 280.11081, found: 280.1116. **$[\alpha]_D^{20}$** = +141.2° (*c* 1.00, $CHCl_3$, 91 %*ee*, *R*). **HPLC analysis:** 91 %*ee*; Daicel Chiralcel OD-H column, 95/5 *n*-hexane/isopropanol, flow rate = 0.7 ml/min, UV = 210 nm, *T* = 20 °C, t_R(major) = 16.1 min, t_R(minor) = 17.9 min.

(*R*)-*tert*-Butyl methyl(1-phenylethyl)carbamate (4b)

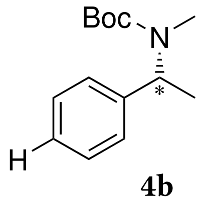

4b

Known Compound.[5] Reduction of **2b** was carried out according to **GP4** (1.5 mol% **1b**, 14 h). Boc protection following **GP5** yielded **4b** as colorless oil in 91 % yield. **^{1}H NMR (600.13 MHz, CD_3CN, 333 K):** δ [ppm] = 7.37–7.25 (m, 5H), 5.39 (q, *J* = 6.8 Hz, 1H), 2.60 (s, 3H), 1.49 (d, *J* = 7.2 Hz, 3H), 1.46 (s, 9H). **^{13}C NMR (150.90 MHz, CD_3CN, 333 K):** δ [ppm] = 156.9, 143.4, 129.5, 128.1, 128.0, 80.2, 54.3, 29.3, 28.9, 17.3. **HR-MS (EI$^+$):** $[M]^+$ = $C_{14}H_{21}NO_2^+$, calcd.: 253.15668, found: 253.15705. **$[\alpha]_D^{20}$** = +98.2° (*c* 1.00, $CHCl_3$, 94 %*ee*, *R*); Lit:[5] **$[\alpha]_D^{25}$** = +93.8° (*c* 0.65, $CHCl_3$, 91 %*ee*, *R*). **HPLC analysis:** 94 %*ee*; Daicel Chiralpak AD-H column, 99.5/0.5 *n*-hexane/isopropanol, flow rate = 1.0 ml/min, UV = 210 nm, *T* = 20 °C, t_R(major) = 10.6 min, t_R(minor) = 12.4 min. The absolute configuration was determined by comparison of the chromatogram obtained for the experimental sample and the one derived from commercially available, enantiomerically pure (*S*)-*N*-methyl-1-phenylethylamine.

(*R*)-*tert*-Butyl (1-(4-chlorophenyl)ethyl)(methyl)carbamate (4c)

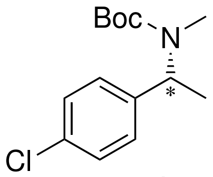

4c

Known Compound.[5] Reduction of **2c** was carried out according to **GP4** (1.5 mol% **1b**, 14 h). Boc protection following **GP5** yielded **4c** as colorless oil in 93 % yield. **^{1}H NMR (600.13 MHz, CD_3CN, 333 K):** δ [ppm] = 7.36–7.35 (m, 2H), 7.29–7.27 (m, 2H), 5.35 (q, *J* = 6.9 Hz, 1H), 2.61 (s, 3H), 1.48 (d, *J* = 7.1 Hz, 3H), 1.45 (s, 9H). **^{13}C NMR (150.90 MHz, CD_3CN, 333 K):** δ [ppm] = 156.8, 142.0, 133.5, 129.7, 129.5, 80.4, 53.9, 29.3, 28.9, 17.3. **HR-MS (EI$^+$):** $[M]^+$ = $C_{14}H_{20}NClO_2^+$, calcd.: 269.11771, found: 269.11730. **$[\alpha]_D^{20}$** = +109.0° (*c* 1.00, $CHCl_3$, 99 %*ee*, *R*); Lit:[5] **$[\alpha]_D^{25}$** = +121° (*c* 1.57, $CHCl_3$, 91 %*ee*, *R*). **HPLC analysis:** 99 %*ee*; Daicel Chiralpak AD-H column, 99/1 *n*-hexane/isopropanol, flow rate = 1.0 ml/min, UV = 210 nm, *T* = 20 °C, t_R(major) = 7.0 min, t_R(minor) = 8.4 min.

(*R*)-*tert*-Butyl methyl(1-(p-tolyl)ethyl)carbamate (4d)

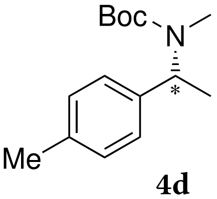

4d

Known Compound.[5] Reduction of **2d** was carried out according to **GP4** (1.5 mol% **1b**, 28 h). Boc protection following **GP5** yielded **4d** as light yellow oil in >99 % yield. **^{1}H NMR (600.13 MHz, CD_3CN, 333 K):** δ [ppm] = 7.19–7.16 (m, 4H), 5.36 (q, *J* = 6.8 Hz, 1H), 2.58 (s, 3H), 2.32 (s, 3H), 1.47–1.46 (m, 12H). **^{13}C NMR (150.90 MHz, CD_3CN, 333 K):** δ [ppm] = 156.9, 140.2, 137.8, 130.1, 128.0, 80.1, 54.0, 29.2, 29.0, 21.2, 17.3. **HR-MS (EI$^+$):** $[M]^+$ = $C_{15}H_{23}NO_2^+$, calcd.: 249.17233, found: 249.17274. **$[\alpha]_D^{20}$** = +84.7° (*c* 1.00, $CHCl_3$, 92 %*ee*, *R*); Lit:[5] **$[\alpha]_D^{25}$** = +105.7° (*c* 1.91, $CHCl_3$, 90 %*ee*, *R*). **HPLC analysis:** 92 %*ee*; Daicel Chiralpak AD-H column, 98/2 *n*-hexane/isopropanol, flow rate = 1.0 ml/min, UV = 230 nm, *T* = 20 °C, t_R(major) = 6.2 min, t_R(minor) = 7.7 min.

(*R*)-*tert*-Butyl (1-([1,1'-biphenyl]-4-yl)ethyl)(methyl)carbamate (4e)

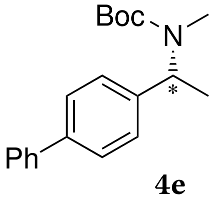

4e

Reduction of **2e** was carried out according to **GP4** (1.5 mol% **1b**, 14 h). Boc protection following **GP5** yielded **4e** as colorless solid in 99 % yield. **^{1}H NMR (600.13 MHz, CD_3CN, 333 K):** δ [ppm] = 7.65–7.62 (m, 4H), 7.47–7.45 (m, 2H), 7.39–7.35 (m, 3H), 5.44 (q, *J* = 6.8 Hz, 1H), 2.65 (s, 3H), 1.53 (d, *J* = 7.0 Hz, 3H), 1.48 (s, 9H). **^{13}C NMR (150.90 MHz, CD_3CN, 333 K):** δ [ppm] = 156.9, 142.6, 141.8, 140.9, 130.1, 128.6, 128.6, 128.1, 128.0, 80.3, 54.1, 29.4, 29.0, 17.3. **HR-MS (EI$^+$):** $[M]^+$ = $C_{20}H_{25}NO_2^+$, calcd.: 311.18798, found: 311.18782. **$[\alpha]_D^{20}$** = +126.8° (*c* 1.02,

$CHCl_3$, 97 %*ee*, *R*). **HPLC analysis:** 97 %*ee*; Daicel Chiralpak AD-H column, 98/2 *n*-hexane/isopropanol, flow rate = 1.0 ml/min, UV = 210 nm, T = 20 °C, t_R(major) = 7.2 min, t_R(minor) = 8.1 min.

(*R*)-*tert*-Butyl (1-(4-methoxyphenyl)ethyl)(methyl)carbamate (4f)

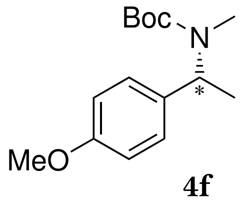

4f

Known Compound.[5] Reduction of **2f** was carried out according to **GP4** (3.0 mol% **1b**, 56 h). Boc protection following **GP5** yielded **4f** as yellow oil in 83 % yield. **^{1}H NMR (600.13 MHz, CD_3CN, 333 K):** δ [ppm] = 7.23–7.21 (m, 2H), 6.91–6.89 (m, 2H), 5.35 (q, *J* = 6.9 Hz, 1H), 3.79 (s, 3H), 2.56 (s, 3H), 1.46–1.45 (m, 12H). **^{13}C NMR (150.90 MHz, CD_3CN, 333 K):** δ [ppm] = 160.1, 156.8, 135.3, 129.3, 115.0, 80.1, 56.2, 53.7, 29.1, 29.0, 17.3. **HR-MS (ESI$^+$):** $[M+Na]^+$ = $C_{15}H_{23}NO_3^+$, calcd.: 288.1573, found: 288.157. **$[\alpha]_D^{20}$** = +101.6° (*c* 1.00, $CHCl_3$, 90 %*ee*, *R*); Lit:[5] **$[\alpha]_D^{25}$** = +110° (*c* 1.4, $CHCl_3$, 92 %*ee*, *R*). **HPLC analysis:** 90 %*ee*; Daicel Chiralpak AD-H column, 98/2 *n*-hexane/isopropanol, flow rate = 1.0 ml/min, UV = 210 nm, T = 20 °C, t_R(major) = 8.1 min, t_R(minor) = 10.0 min.

(*R*)-*tert*-Butyl methyl(1-(m-tolyl)ethyl)carbamate (4g)

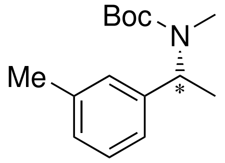

4g

Known Compound.[5] Reduction of **2g** was carried out according to **GP4** (1.5 mol% **1b**, 14 h). Boc protection following **GP5** yielded **4g** as colorless oil in 93 % yield. **^{1}H NMR (600.13 MHz, CD_3CN, 333 K):** δ [ppm] = 7.23 (t, *J* = 7.6 Hz, 1H), 7.12 (s, 1H), 7.09–7.08 (m, 2H), 5.35 (q, *J* = 6.7 Hz, 1H), 2.59 (s, 3H), 2.34 (s, 3H), 1.47 (d, *J* = 7.0 Hz, 3H), 1.46 (s, 9H). **^{13}C NMR (150.90 MHz, CD_3CN, 333 K):** δ [ppm] = 156.9, 143.4, 139.2, 129.4, 128.8, 128.7, 125.0, 80.2, 54.2, 29.3, 28.9, 21.7, 17.3. **HR-MS (EI$^+$):** $[M]^+$ = $C_{15}H_{23}NO_2^+$, calcd.: 249.17233, found: 249.17288. **$[\alpha]_D^{20}$** = +92.8° (*c* 1.01, $CHCl_3$, 91 %*ee*, *R*); Lit:[5] **$[\alpha]_D^{25}$** = +99° (*c* 1.21, $CHCl_3$, 90 %*ee*, *R*). **HPLC analysis:** 91 %*ee*; Daicel Chiralpak AD-H column, 99.5/0.5 *n*-hexane/isopropanol, flow rate = 0.8 ml/min, UV = 210 nm, T = 20 °C, t_R(major) = 12.0 min, t_R(minor) = 13.4 min.

(*R*)-*N*-(1-(3-Fluorophenyl)ethyl)-*N*-methylbenzamide (4h)

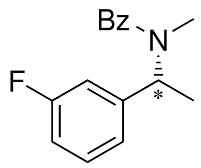

4h

Reduction of **2h** was carried out according to **GP4** (1.5 mol% **1b**, 14 h). Bz protection following **GP6** yielded **4h** as light yellow oil in 88 % yield. **^{1}H NMR (600.13 MHz, CD_3CN, 333 K):** δ [ppm] = 7.45–7.38 (m, 6H), 7.18–7.02 (m, 3H), 5.62 (*br* s, 1H), 2.69 (s, 3H), 1.59 (d, *J* = 7.0 Hz, 3H). **^{13}C NMR (150.90 MHz, CD_3CN, 333 K):** δ [ppm] = 172.5 (s), 164.3 (d, *J* = 243.8 Hz), 145.3 (d, *J* = 5.9 Hz), 138.6 (s), 131.5 (d, *J* = 8.2 Hz)), 130.4 (s), 129.7 (s), 127.7 (s), 124.1 (s), 115.1 (d, *J* = 21.2 Hz), 115.0 (d, *J* = 22.3 Hz), 17.0. **^{19}F NMR (376.23 MHz, CD_3CN, 295 K):** δ [ppm] = −114.4, −114.8. **HR-MS (ESI$^+$):** $[M-H+Na]^+$ = $C_{16}H_{15}NFONa^+$, calcd.: 279.10354, found: 279.1047. **$[\alpha]_D^{20}$** = +152.1° (*c* 0.79, $CHCl_3$, 99 %*ee*, *R*). **HPLC analysis:** 99 %*ee*; Daicel Chiralcel OD-H column, 95/5 *n*-hexane/isopropanol, flow rate = 0.7 ml/min, UV = 210 nm, *T* = 30 °C, t_R(major) = 16.5 min, t_R(minor) = 18.9 min.

(*R*)-*N*-Methyl-*N*-(1-(3-(trifluoromethyl)phenyl)ethyl)benzamide (4i)

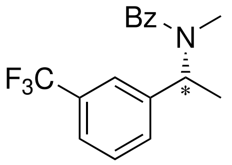

4i

Reduction of **2i** was carried out according to **GP4** (0.5 mol% **1b**, 14 h). Bz protection following **GP6** yielded **4i** as yellow oil in 91 % yield. **^{1}H NMR (600.13 MHz, CD_3CN, 333 K):** δ [ppm] = 7.63–7.57 (m, 4H), 7.45–7.42 (m, 5H), 5.67 (*br* s, 1H), 2.70 (s, 3H), 1.64 (d, *J* = 7.0 Hz, 3H). **^{13}C NMR (150.90 MHz, CD_3CN, 333 K):** δ [ppm] = 172.7 (s), 143.8 (s), 138.5 (s), 132.1 (s), 131.5 (q, *J* = 32.0 Hz), 130.6 (s), 130.5 (s), 129.7 (s), 127.7 (s), 126.6 (s), 125.7 (q, *J* = 271.5 Hz), 125.2 (q, *J* = 3.9 Hz), 124.8 (m), 16.9 (s). **^{19}F NMR (376.23 MHz, CD_3CN, 295 K):** δ [ppm] = −63.0. **HR-MS (ESI$^+$):** $[M-H+Na]^+$ = $C_{17}H_{15}NF_3ONa^+$, calcd.: 329.10034, found: 329.1005. **$[\alpha]_D^{20}$** = +130.4° (*c* 1.00, $CHCl_3$, 99 %*ee*, *R*). **HPLC analysis:** 99 %*ee*; Daicel Chiralcel OD-H column, 95/5 *n*-hexane/isopropanol, flow rate = 0.7 ml/min, UV = 210 nm, *T* = 30 °C, t_R(major) = 14.3 min, t_R(minor) = 17.0 min.

(*R*)-*tert*-Butyl methyl(1-(o-tolyl)ethyl)carbamate (4j)

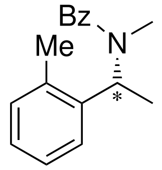

4j

Reduction of **2j** was carried out according to **GP4** (3.0 mol% **1b**, 56 h). Bz protection following **GP6** yielded **4j** as yellow oil in 31 % yield. **^{1}H NMR (600.13 MHz, CD_3CN, 333 K):** δ [ppm] = 7.42–7.36 (m, 6H), 7.26–7.23 (m, 3H), 5.87 (*br* s, 1H), 2.53 (s, 3H), 2.28 (s, 3H), 1.59 (d, *J* = 6.9 Hz, 3H). **^{13}C NMR (150.90 MHz, CD_3CN, 333 K):** δ [ppm] = 171.7, 139.8, 138.9, 138.7, 131.8, 130.3, 129.5, 128.7,

128.2, 127.7, 127.0, 51.1, 31.9, 19.5, 16.6. **HR-MS (ESI$^+$):** $[M+Na]^+$ = $C_{17}H_{19}NONa^+$, calcd.: 275.12661, found: 275.1280. **HPLC analysis:** 19 %*ee*; Daicel Chiralcel OD-H column, 98/2 *n*-hexane/isopropanol, flow rate = 0.7 ml/min, UV = 210 nm, *T* = 30 °C, t_R(major) = 23.2 min, t_R(minor) = 25.9 min.

(*R*)-*N*-(1-(Furan-2-yl)ethyl)-*N*-methylbenzamide (4k)

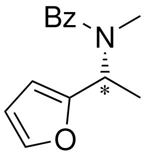

4k

Reduction of **2k** was carried out according to **GP4** (3.0 mol% **1b**, 56 h). Bz protection following **GP6** yielded **4k** as apricot oil in 61 % yield. **^{1}H NMR (600.13 MHz, CD_3CN, 333 K):** δ [ppm] = 7.47–7.45 (m, 6H), 6.40–6.39 (m, 2H), 5.89–5.03 (*br* m, 1H), 2.70 (s, 3H), 1.51 (d, *J* = 6.9 Hz, 3H). **^{13}C NMR (150.90 MHz, CD_3CN, 333 K):** δ [ppm] = 172.3, 155.6, 143.7, 138.5, 130l4, 129.6, 127.8, 111.4, 108.7, 16.3. **HR-MS (ESI$^+$):** $[M-H+Na]^+$ = $C_{14}H_{14}NO_2Na^+$, calcd.: 251.09222, found: 251.0942. **$[\alpha]_D^{20}$** = +61.7° (*c* 0.60, $CHCl_3$, 32 %*ee*, *R*). **HPLC analysis:** 32 %*ee*; Daicel Chiralcel OD-H column, 95/5 *n*-hexane/isopropanol, flow rate = 0.7 ml/min, UV = 210 nm, *T* = 30 °C, t_R(major) = 16.1 min, t_R(minor) = 18.0 min.

(*R*)-*tert*-Butyl methyl(1-(thiophen-2-yl)ethyl)carbamate (4l)

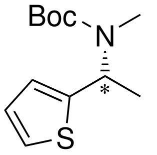

4l

Reduction of **2l** was carried out according to **GP4** (3.0 mol% **1b**, 56 h). Boc protection following **GP5** yielded **4l** as brown oil in 71 % yield. **^{1}H NMR (600.13 MHz, CD_3CN, 333 K):** δ [ppm] = 7.30 (dd, *J* = 5.1 Hz, *J* = 1.0 Hz), 1H), 6.98–6.95 (m, 2H), 5.58 (q, *J* = 6.5 Hz, 1H), 2.63 (s, 3H), 1.54 (d, *J* = 7.0 Hz, 3H), 1.47 (s, 3H). **^{13}C NMR (150.90 MHz, CD_3CN, 333 K):** δ [ppm] = 156.5, 147.6, 127.8, 125.8, 125.6, 80.5, 51.0, 28.9, 18.7. **HR-MS (ESI$^+$):** $[M+Na]^+$ = $C_{12}H_{19}NO_2S^+$, calcd.: 264.1029, found: 264.1029. **$[\alpha]_D^{20}$** = +47.9° (*c* 1.00, $CHCl_3$, 50 %*ee*, *R*). **HPLC analysis:** 50 %*ee*; Daicel Chiralpak AD-H column, 99.5/0.5 *n*-hexane/isopropanol, flow rate = 1.0 ml/min, UV = 210 nm, *T* = 20 °C, t_R(major) = 9.0 min, t_R(minor) = 11.5 min.

(*R*)-*tert*-Butyl methyl(1-(naphthalen-2-yl)ethyl)carbamate (4m)

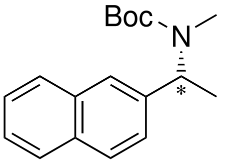

4m

Reduction of **2m** was carried out according to **GP4** (3.0 mol% **1b**, 56 h). Boc protection following **GP5** yielded **4m** as off-white solid in 80 % yield. **^{1}H NMR (600.13 MHz, CD_3CN, 333 K):** δ [ppm] = 7.89–7.85 (m, 3H), 7.78 (s, 1H), 7.52–7.48 (m, 2H), 7.43 (dd, *J* = 8.6 Hz, *J* = 1.7 Hz, 1H), 5.55–5.54 (m, 1H), 2.63 (s, 3H), 1.61 (d, *J* = 7.1 Hz, 3H), 1.48 (s, 9H). **^{13}C NMR (150.90 MHz, CD_3CN,**

333 K): δ [ppm] = 157.0, 141.0, 134.6, 133.8, 129.1, 129.1, 128.6, 127.4, 127.1, 126.9, 126.2, 80.3, 54.5, 29.4, 29.4, 29.0, 17.2. **HR-MS (ESI$^+$):** $[M+Na]^+$ = $C_{18}H_{23}NO_2^+$, calcd.: 308.1621, found: 308.1623. **$[\alpha]_D^{20}$** = +158.9° (*c* 1.00, $CHCl_3$, 94 %*ee*, *R*). **HPLC analysis:** 94 %*ee*; Daicel Chiralpak AD-H column, 98/2 *n*-hexane/isopropanol, flow rate = 1.0 ml/min, UV = 210 nm, *T* = 20 °C, t_R(major) = 6.6 min, t_R(minor) = 7.4 min.

(*R*)-*N*-(1-(4-Fluorophenyl)butyl)-*N*-methylbenzamide (4n)

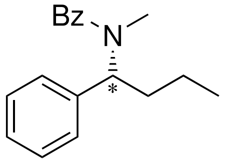

4n

Reduction of **2n** was carried out according to **GP4** (3.0 mol% **1b**, 56 h). Bz protection following **GP6** yielded **4n** as yellow oil in 73 % yield. **^{1}H NMR (600.13 MHz, CD_3CN, 333 K):** δ [ppm] = 7.44–7.29 (m, 10H), 5.84–4.98 (*br* m, 1H), 2.68 (*br* s, 3H), 2.07–1.96 (*br* m, 2H), 1.41 (*br* s, 2H), 0.99 (*br* s, 3H). **^{13}C NMR (150.90 MHz, CD_3CN, 333 K):** δ [ppm] = 172.9, 141.6, 139.0, 130.3, 129.7, 129.6, 128.7, 128.5, 127.7, 33.2 (*br*), 20.7, 14.3. **HR-MS (ESI$^+$):** $[M-H+Na]^+$ = $C_{18}H_{20}NONa^+$, calcd.: 289.14426, found: 289.1476. **$[\alpha]_D^{20}$** = +151.2° (*c* 1.00, $CHCl_3$, 94 %*ee*, *R*). **HPLC analysis:** 94 %*ee*; Daicel Chiralcel OD-H column, 99.5/0.5 *n*-hexane/isopropanol, flow rate = 1.0 ml/min, UV = 210 nm, *T* = 30 °C, t_R(major) = 48.8 min, t_R(minor) = 45.4 min.

(*S*)-*tert*-Butyl (2,3-dihydro-1*H*-inden-1-yl)(methyl)carbamate (4o)

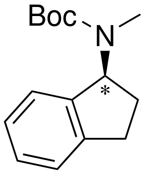

4o

Known Compound.[5] Reduction of **2o** was carried out according to **GP4** (3.0 mol% **1b**, 56 h). Boc protection following **GP5** yielded **4o** as yellow oil in 74 % yield. **^{1}H NMR (600.13 MHz, CD_3CN, 333 K):** δ [ppm] = 7.26–7.20 (m, 3H), 7.13–7.12 (m, 1H), 5.71 (*br* s, 1H), 3.02–2.97 (m, 1H), 2.88–2.83 (m, 1H), 2.56 (s, 3H), 2.36–2.31 (m, 1H), 2.01–1.96 (m, 1H), 1.48 (s, 9H). **^{13}C NMR (150.90 MHz, CD_3CN, 333 K):** δ [ppm] = 157.2, 144.8, 143.2, 128.8, 127.6, 126.0, 125.1, 80.2, 62.3, 31.1, 29.9, 29.9, 28.9. **HR-MS (ESI$^+$):** $[M-H+Na]^+$ = $C_{15}H_{20}NO_2Na^+$, calcd.: 269.13917, found: 269.1398. **$[\alpha]_D^{20}$** = −10.5° (*c* 1.01, $CHCl_3$, 20 %*ee*, *R*); Lit:[5] **$[\alpha]_D^{25}$** = +47.7° (*c* 1.45, $CHCl_3$, 81 %*ee*, *R*). **HPLC analysis:** 20 %*ee*; Daicel Chiralpak AD-H column, 99/1 *n*-hexane/isopropanol, flow rate = 1.0 ml/min, UV = 210 nm, *T* = 20 °C, t_R(major) = 7.5 min, t_R(minor) = 9.3 min.

tert-Butyl 1-methyl-3,4-dihydroisoquinoline-2(1_H_)-carboxylate (4p)

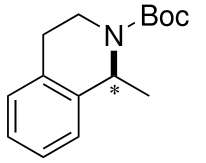

4p

Known Compound.[13] Reduction of **2p** was carried out according to **GP4** (3.0 mol% **1b**, 56 h). Boc protection following **GP5** yielded **4p** as yellow oil in 86 % yield. **^{1}H NMR (600.13 MHz, CD_3CN, 333 K):** δ [ppm] = 7.20–7.13 (m, 4H), 5.16 (q, *J* = 6.6 Hz, 1H), 4.03–4.01 (m, 1H), 3.28–3.20 (m, 1H), 2.87–2.82 (m, 1H), 2.78–2.74 (m, 1H), 1.48 (s, 9H), 1.42 (d, *J* = 6.8 Hz, 3H). **^{13}C NMR (150.90 MHz, CD_3CN, 333 K):** δ [ppm] = 155.6, 140.2, 135.5, 130.0, 128.1, 127.5, 127.3, 80.3, 51.4, 38.6, 29.8, 28.9, 22.6. **HR-MS (ESI$^+$):** $[M\text{-}H_2\text{+}Na]^+$ = $C_{15}H_{19}NO_2Na^+$, calcd.: 269.13863, found: 269.1392. **HPLC analysis:** 0 %*ee*; Daicel Chiralpak AD-H column, 98/2 *n*-hexane/isopropanol, flow rate = 1.0 ml/min, UV = 210 nm, *T* = 20 °C, t_R(major) = 7.5 min, t_R(minor) = 8.5 min.

(_R_)-_tert_-Butyl 2-(4-fluorophenyl)pyrrolidine-1-carboxylate (4q)

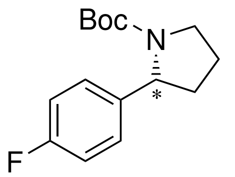

4q

Known Compound.[14] Reduction of **2q** was carried out according to **GP4** (1.5 mol% **1b**, 28 h). Boc protection following **GP5** yielded **4q** as colorless solid in 93 % yield. **^{1}H NMR (600.13 MHz, CD_3CN, 333 K):** δ [ppm] = 7.23–7.19 (m, 2H), 7.06–7.02 (m, 2H), 4.79 (*br* s, 1H), 3.57–3.51 (m, 2H), 2.35–2.29 (m, 1H), 1.90–1.78 (m, 2H), 1.78–1.73 (m, 1H), 1.29 (*br* s, 9H). **^{13}C NMR (150.90 MHz, CD_3CN, 333 K):** δ [ppm] = 162.8 (d, *J* = 241.8 Hz), 155.4 (s), 142.7 (*br* s), 128.6 (d, *J* = 8.0 Hz), 115.8 (d, *J* = 21.8 Hz), 79.8 (s), 61.7 (s), 48.3 (s), 36.6 (s), 28.8 (s), 24.3 (s). **^{19}F NMR (376.23 MHz, CD_3CN, 295 K):** δ [ppm] = −118.7, −118.8. **HR-MS (EI$^+$):** $[M]^+$ = $C_{15}H_{20}FNO_2^+$, calcd.: 265.14726, found: 265.14598. $[\alpha]_D^{25}$ = +60.5° (*c* 1.02, $CHCl_3$, 92 %*ee*, *R*); Lit:[14] $[\alpha]_D^{22}$ = +76.3° (*c* 1.0, CH_2Cl_2, 94 %*ee*, *R*). **HPLC analysis:** 92 %*ee*; Daicel Chiralpak AD-H column, 98/2 *n*-hexane/isopropanol, flow rate = 1.0 ml/min, UV = 210 nm, *T* = 20 °C, t_R(major) = 7.3 min, t_R(minor) = 8.4 min.

(_R_)-_tert_-Butyl (1-(4-fluorophenyl)ethyl)(propyl)carbamate (4r)

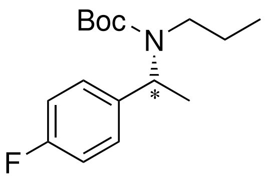

4r

Reduction of **2r** was carried out according to **GP4** (3.0 mol% **1b**, 56 h). Boc protection following **GP5** yielded **4r** as colorless oil in 91 % yield. **^{1}H NMR (600.13 MHz, CD_3CN, 333 K):** δ [ppm] = 7.35–7.32 (m, 2H), 7.09–7.04 (m, 2H), 5.24–5.23 (m, 1H), 3.04–2.99 (m, 1H), 2.94–2.90 (m, 1H), 1.52 (d, *J* = 7.1 Hz, 3H), 1.47–1.35 (m, 11H), 0.77 (t, *J* = 7.4 Hz, 3H). **^{13}C NMR (150.90 MHz,**

CD_3CN, 333 K): δ [ppm] = 163.0 (d, *J* = 242.9 Hz), 156.7, 140.2 (d, *J* = 3.1 Hz), 130.0 (d, *J* = 8.1 Hz), 115.9 (d, *J* = 21.4 Hz), 80.1 (s), 54.6 (s), 47.1 (s), 28.9 (s), 24.2 (s), 18.5 (s), 11.8 (s). **^{19}F NMR (376.23 MHz, CD_3CN, 295 K):** δ [ppm] = −117.9. **HR-MS (ESI$^+$):** $[M\text{-}H\text{+}Na]^+$ = $C_{16}H_{23}NFO_2^+$, calcd.: 303.16105, found: 303.1643. **$[\alpha]_D^{20}$** = +68.4° (*c* 1.01, $CHCl_3$, 83 %*ee*, *R*). **HPLC analysis:** 83 %*ee*; Daicel Chiralpak AD-H column, 98/2 *n*-hexane/isopropanol, flow rate = 0.5 ml/min, UV = 230 nm, *T* = 20 °C, t_R(major) = 10.0 min, t_R(minor) = 10.8 min.

(*R*)-*tert*-Butyl (1-(4-fluorophenyl)ethyl)(hexyl)carbamate (4s)

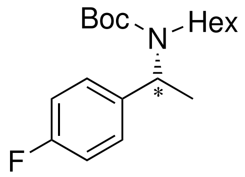

4s

Reduction of **2s** was carried out according to **GP4** (3.0 mol% **1b**, 56 h). Boc protection following **GP5** yielded **4s** as light yellow oil in 86 % yield. **^{1}H NMR (600.13 MHz, CD_3CN, 333 K):** δ [ppm] = 7.35–7.32 (m, 2H), 7.08–7.04 (m, 2H), 5.24–5.23 (m, 1H), 3.06–3.01 (m, 1H), 2.98–2.93 (m, 1H), 1.52–1.51 (m, 4H), 1.43 (s, 9H), 1.37–1.14 (m, 7H), 0.87 (t, *J* = 7.1 Hz, 3H). **^{13}C NMR (150.90 MHz, CD_3CN, 333 K):** δ [ppm] = 163.8 (d, *J* = 243.0 Hz), 156.6 (s), 140.2 (d, *J* = 3.2 Hz), 130.1 (d, *J* = 8.1 Hz), 115.9 (d, *J* = 21.4 Hz), 80.1 (s), 54.5 (s), 45.4 (s), 32.4 (s), 30.9 (s), 28.9 (s), 27.6 (s), 23.4 (s), 18.5 (s), 14.3 (s). **^{19}F NMR (376.23 MHz, CD_3CN, 295 K):** δ [ppm] = −117.8. **HR-MS (ESI$^+$):** $[M\text{-}H\text{+}Na]^+$ = $C_{19}H_{29}NFO_2Na^+$, calcd.: 345.20800, found: 345.2083. **$[\alpha]_D^{20}$** = +61.1° (*c* 1.01, $CHCl_3$, 91 %*ee*, *R*). **HPLC analysis:** 91 %*ee*; Daicel Chiralpak AD-H column, 99.5/0.5 *n*-hexane/isopropanol, flow rate = 1.0 ml/min, UV = 210 nm, *T* = 20 °C, t_R(major) = 9.2 min, t_R(minor) = 10.2 min.

(*R*)-*N*-Benzyl-1-(4-fluorophenyl)ethan-1-amine (3t)

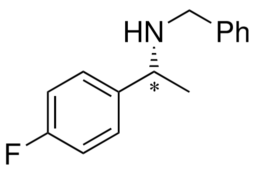

3t

Known Compound.[15] Reduction of **2t** was carried out according to **GP4** (1.5 mol% **1b**, 28 h). Amine **3t** was obtained after column chromatography (*n*-hexane/DCM) as yellow oil in 97 % yield. **^{1}H NMR (600.13 MHz, $CDCl_3$, 295 K):** δ [ppm] = 7.32–7.29 (m, 4H), 7.26–7.22 (m, 3H), 7.03–6.99 (m, 2H), 3.79 (q, *J* = 6.5 Hz, 1H), 3.62 (d, *J* = 13.1 Hz, 1H), 3.56 (d, *J* = 13.2 Hz, 1H), 1.33 (d, *J* = 6.6 Hz, 3H). **^{13}C NMR (150.90 MHz, $CDCl_3$, 295 K):** δ [ppm] = 161.9 (d, *J* = 244.2 Hz), 141.3 (d, *J* = 3.1 Hz), 140.6 (s), 128.5 (s), 128.3 (d, *J* = 7.8 Hz), 128.2 (s), 127.1 (s), 115.3 (d, *J* = 21.1 Hz), 56.9 (s), 51.7 (s), 24.8 (s). **^{19}F NMR (376.23 MHz, $CDCl_3$, 295 K):** δ [ppm] = −116.2. **HR-MS (ESI$^+$):** $[M]^+$ = $C_{15}H_{16}NF^+$, calcd.: 229.12668, found: 229.1281. **$[\alpha]_D^{20}$** = +45.5° (*c* 1.00, $CHCl_3$, 98 %*ee*, *R*). *ee* determination after derivatization into **3t-Bz**: **HPLC analysis:** 98 %*ee*; Daicel

Chiralpak AD-H column, 90/10 *n*-hexane/isopropanol, flow rate = 1.0 ml/min, UV = 210 nm, T = 30 °C, t_R(major) = 15.7 min, t_R(minor) = 23.4 min.

(*R*)-1-(4-Fluorophenyl)-*N*-(thiophen-2-ylmethyl)ethan-1-amine (3u)

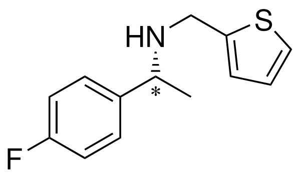

3u

Reduction of **2u** was carried out according to **GP4** (0.5 mol% **1b**, 14 h). Amine **3u** was obtained after column chromatography (*n*-hexane/DCM) as yellow oil in 91 % yield. **^{1}H NMR (399.89 MHz, $CDCl_3$, 295 K):** δ [ppm] = 7.34–7.32 (m, 2H), 7.21 (dd, J = 5.1 Hz, J = 1.2 Hz, 1H), 7.05–7.00 (m, 2H), 6.93 (dd, J = 5.1 Hz, J = 3.4 Hz, 1H), 6.85 (dd, J = 3.4 Hz, J = 0.9 Hz, 1H), 3.85 (q, J = 6.6 Hz, 1H), 3.81–3.80 (m, 2H), 1.34 (d, J = 6.6 Hz, 3H). **^{13}C NMR (100.55 MHz, $CDCl_3$, 295 K):** δ [ppm] = 162.0 (d, J = 244.4 Hz), 144.4 (s), 141.0 (d, J = 3.1 Hz), 128.3 (d, J = 7.9 Hz), 126.7 (s), 124.9 (s), 124.4 (s), 115.5 (d, J = 21.1 Hz), 56.5 (s), 46.1 (s), 24.6 (s). **^{19}F NMR (376.23 MHz, $CDCl_3$, 295 K):** δ [ppm] = −116.1. **HR-MS (EI$^+$):** $[M]^+$ = $C_{13}H_{14}FNS^+$, calcd.: 235.08255, found: 235.08186. **$[\alpha]_D^{20}$** = +56.2° (*c* 1.01, $CHCl_3$, >99 %*ee*, *R*). *ee* determination after derivatization into **3u-Bz**: **HPLC analysis:** >99 %*ee*; Daicel Chiralpak AD-H column, 90/10 *n*-hexane/isopropanol, flow rate = 1.0 ml/min, UV = 210 nm, T = 30 °C, t_R(major) = 23.2 min, t_R(minor) = 28.5 min.

3.4 Synthesis of Pharmaceuticals

Synthesis of (*R*)-Fendilin (6)

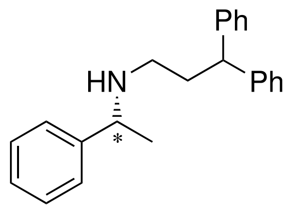

6

Protioligand ^{Me,Ph}boxmi-H (7.36 mg, 15.95 µmol, 0.5 mol%) and (tmeda)Fe(CH_2SiMe_3)$_2$ (5.53 mg, 15.95 µmol, 0.5 mol %) were dissolved in 1.0 ml toluene and stirred at room temperature for 6 h. Imine **5** (1.0 g, 3.19 mmol, 1.0 eq.), 1.0 ml toluene and 10 ml *n*-hexane were then added to the brown solution. The reaction mixture was cooled to −40 °C and neat pinacolborane (817 mg, 6.38 mmol, 926 µl, 2.0 eq.) was added in one portion. After the mixture was warmed to room temperature over a period of 14 h, the reaction was quenched by addition of excess silica in small portions. The suspension was stirred at room temperature for 2 h and filtered. The crude product was eluted using a DCM/NEt_3 mixture, freed from any volatiles under reduced pressure and purified by column chromatography (*n*-hexane/DCM/NEt_3 1:1:0.01). The product was obtained as brown oil in 98% yield. **^{1}H NMR (600.13 MHz, $CDCl_3$, 295 K):** δ [ppm] = 7.34–7.18 (m, 15H), 4.01 (t, *J* = 7.8 Hz, 1H), 3.72 (q, *J* = 6.6 Hz, 1H), 2.54–2.44 (m, 2H), 2.31–2.19 (m, 2H), 1.33 (d, *J* = 6.6 Hz, 3H). **^{13}C NMR (150.90 MHz, $CDCl_3$, 295 K):** δ [ppm] = 145.9, 145.1, 144.8, 128.5, 128.5, 128.0, 127.9, 126.9, 126.6, 126.2, 126.2, 58.3, 49.1, 46.1, 36.2, 24.5. **HR-MS (ESI$^+$):** $[M]^+$ = $C_{23}H_{35}N^+$, calcd.: 315.19870, found: 315.1980. **$[\alpha]_D^{20}$** = +44.5° (*c* 1.00, $CHCl_3$, 98 %*ee*, *R*); Lit:[5] **$[\alpha]_D^{25}$** = +43.46° (*c* 0.98, $CHCl_3$, 95 %*ee*, *R*). *ee* determination after derivatization into **6-Bz**: **HPLC analysis:** 98 %*ee*; Daicel Chiralpak AD-H column, 90/10 *n*-hexane/isopropanol, flow rate = 1.0 ml/min, UV = 210 nm, *T* = 30 °C, t_R(major) = 10.9 min, t_R(minor) = 14.6 min. Spectral properties are in accordance with literature data.[5]

Synthesis of (*R*)-Tecalcet (8)

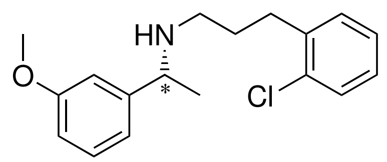

8

Reduction of **7** was carried out following **GP4** (0.5 mol% **1b**, 14 h). Tecalcet **8** was obtained after column chromatography (*n*-hexane/DCM) as brown oil in 97 % yield. **^{1}H NMR (399.89 MHz, $CDCl_3$, 295 K):** δ [ppm] = 7.33–7.31 (m, 1H), 7.26–7.22 (m, 1H), 7.18–7.09 (m, 3H), 6.91–6.89 (m, 2H), 6.80–6.77 (m, 1H), 3.82 (s, 3H), 3.75 (q, *J* = 6.6 Hz, 1H), 2.81–2.66 (m, 2H), 2.61–2.48 (m, 2H), 1.83–1.74 (m, 2H), 1.41 (*br* s, 1H), 1.35 (d, *J* = 6.6 Hz, 3H). **^{13}C NMR (150.90 MHz, $CDCl_3$, 295 K):** δ [ppm] = 159.9, 147.8, 139.9, 134.0, 130.4, 129.5, 129.5, 127.4, 126.8, 119.1, 112.3, 112.2, 58.4, 55.3, 47.4, 31.5, 30.3, 24.5. **HR-MS (ESI$^+$):** $[M]^+$ = $C_{18}H_{22}ClNO^+$, calcd.: 303.13899, found: 303.1407. $[\alpha]_D^{20}$ = +39.9° (*c* 1.00, $CHCl_3$, 94 %*ee*, *R*); Lit:[5] $[\alpha]_D^{25}$ = +36.7° (*c* 1.5, $CHCl_3$, 94 %*ee*, *R*). *ee* determination after derivatization into **8-Bz**: **HPLC analysis:** 94 %*ee*; Daicel Chiralpak AD-H column, 90/10 *n*-hexane/isopropanol, flow rate = 1.0 ml/min, UV = 210 nm, *T* = 30 °C, t_R(major) = 13.6 min, t_R(minor) = 19.2 min. Spectral properties are in accordance with literature data.[16]

4 Chromatographic Data

Compound 4a

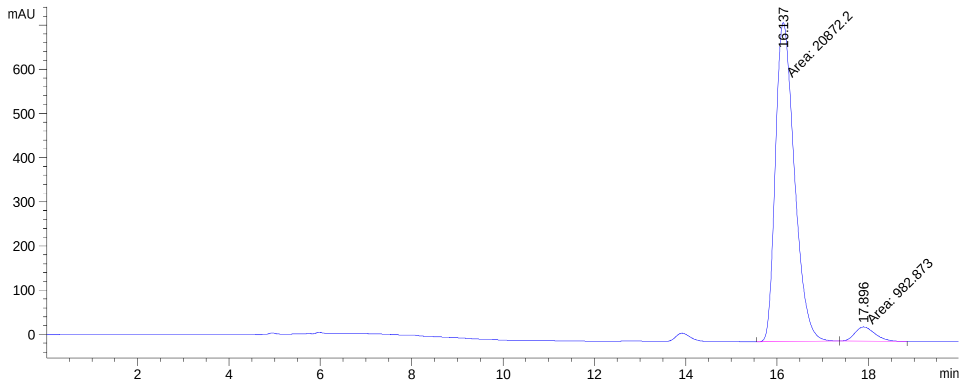

Signal 2: DAD1 C, Sig=210,8 Ref=360,100

Peak #	RetTime [min]	Type	Width [min]	Area [mAU*s]	Height [mAU]	Area %
1	16.137	MM	0.4818	2.08722e4	721.99176	95.5028
2	17.896	MM	0.5093	982.87262	32.16664	4.4972

Racemic Mixture of Compound 4a

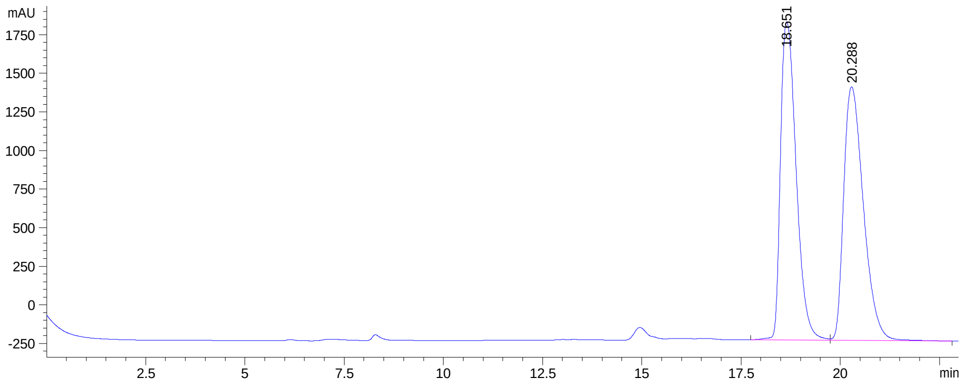

Signal 2: DAD1 C, Sig=210,4 Ref=off

Peak #	RetTime [min]	Type	Width [min]	Area [mAU*s]	Height [mAU]	Area %
1	18.651	BV	0.4303	5.65793e4	2060.92773	49.8527
2	20.288	VB	0.5382	5.69137e4	1642.93250	50.1473

Compound 4b

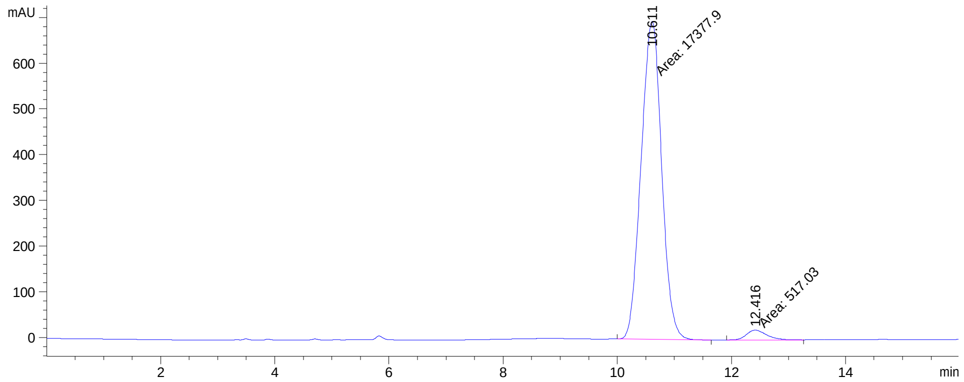

Signal 2: DAD1 C, Sig=210,8 Ref=360,100

Peak #	RetTime [min]	Type	Width [min]	Area [mAU*s]	Height [mAU]	Area %
1	10.611	MM	0.4165	1.73779e4	695.40424	97.1107
2	12.416	MM	0.4052	517.02985	21.26425	2.8893

Totals : 1.78949e4 716.66848

Racemic Mixture of Compound 4b

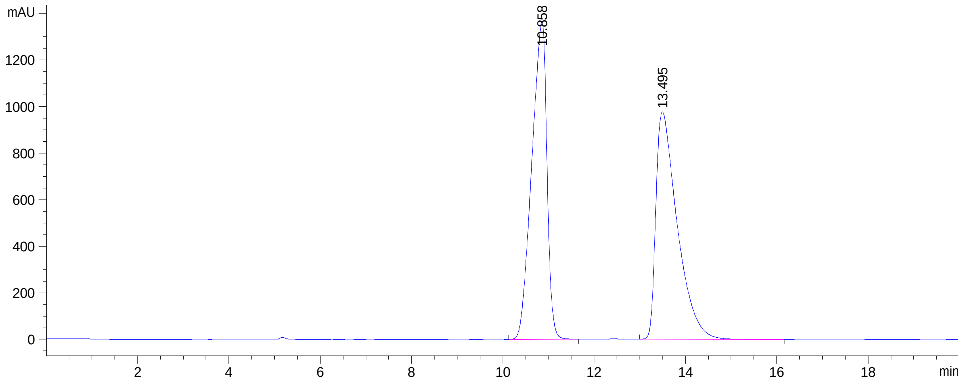

Signal 2: DAD1 C, Sig=210,8 Ref=360,100

Peak #	RetTime [min]	Type	Width [min]	Area [mAU*s]	Height [mAU]	Area %
1	10.858	BB	0.3660	3.14100e4	1367.59558	49.7821
2	13.495	BB	0.4828	3.16849e4	975.77069	50.2179

Compound 4c

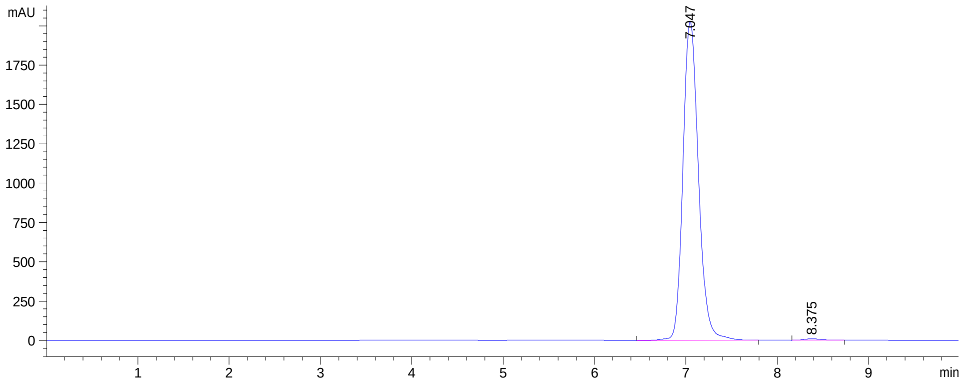

Signal 2: DAD1 C, Sig=210,8 Ref=360,100

Peak #	RetTime [min]	Type	Width [min]	Area [mAU*s]	Height [mAU]	Area %
1	7.047	BB	0.1817	2.39235e4	2027.25916	99.5014
2	8.375	BB	0.1991	119.87887	9.37764	0.4986

Totals : 2.40433e4 2036.63679

Racemic Mixture of Compound 4c

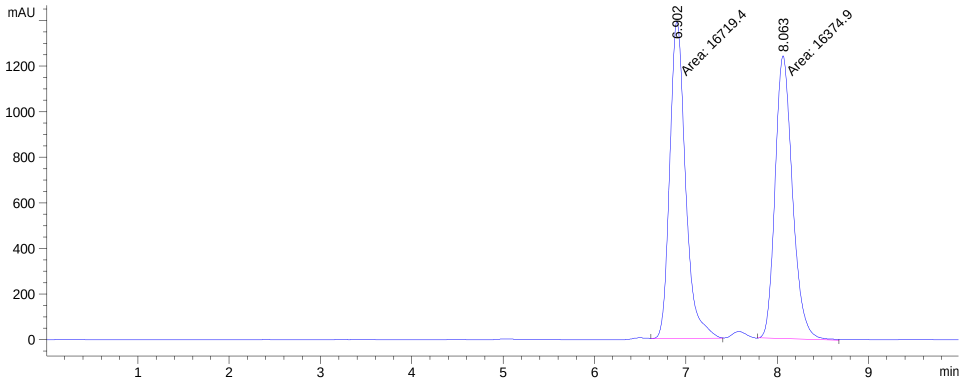

Signal 2: DAD1 C, Sig=210,8 Ref=360,100

Peak #	RetTime [min]	Type	Width [min]	Area [mAU*s]	Height [mAU]	Area %
1	6.902	MM	0.2000	1.67194e4	1393.60181	50.5205
2	8.063	MM	0.2200	1.63749e4	1240.58875	49.4795

Compound 4d

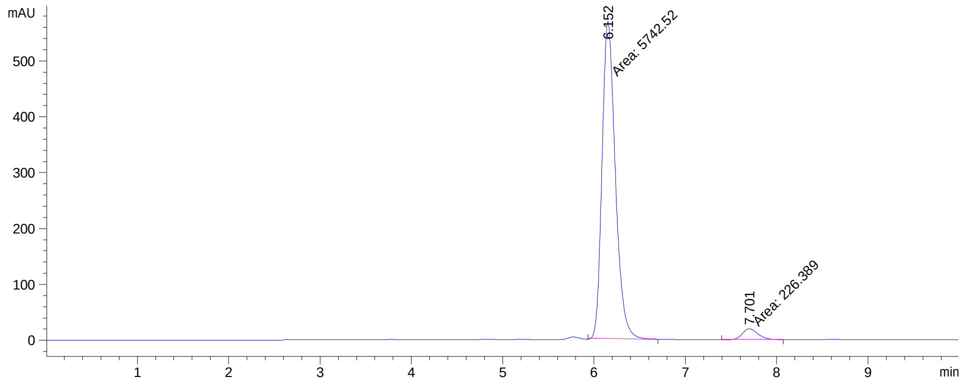

Signal 3: DAD1 D, Sig=230,4 Ref=off

Peak #	RetTime [min]	Type	Width [min]	Area [mAU*s]	Height [mAU]	Area %
1	6.152	MM	0.1685	5742.52100	567.95129	96.2072
2	7.701	MM	0.1942	226.38910	19.42813	3.7928
Totals :				5968.91010	587.37942	

Racemic Mixture of Compound 4d

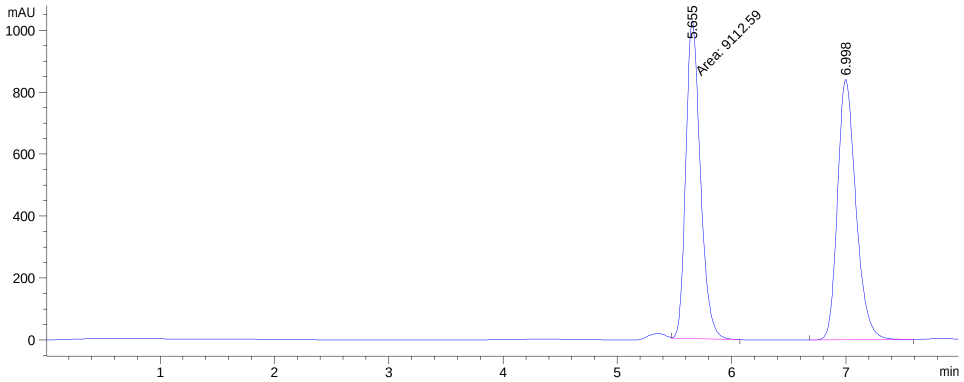

Signal 3: DAD1 D, Sig=215,8 Ref=360,100

Peak #	RetTime [min]	Type	Width [min]	Area [mAU*s]	Height [mAU]	Area %
1	5.655	MM	0.1480	9112.58887	1026.29590	49.2375
2	6.998	BB	0.1705	9394.84277	839.80353	50.7625
Totals :				1.85074e4	1866.09943	

Compound 4e

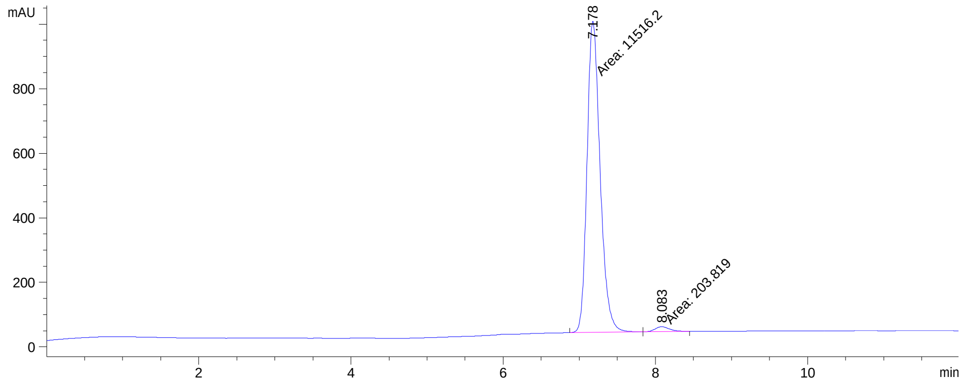

Signal 2: DAD1 C, Sig=210,8 Ref=360,100

Peak #	RetTime [min]	Type	Width [min]	Area [mAU*s]	Height [mAU]	Area %
1	7.178	MM	0.1989	1.15162e4	964.83679	98.2609
2	8.083	MM	0.2207	203.81926	15.39048	1.7391

Racemic Mixture of Compound 4e

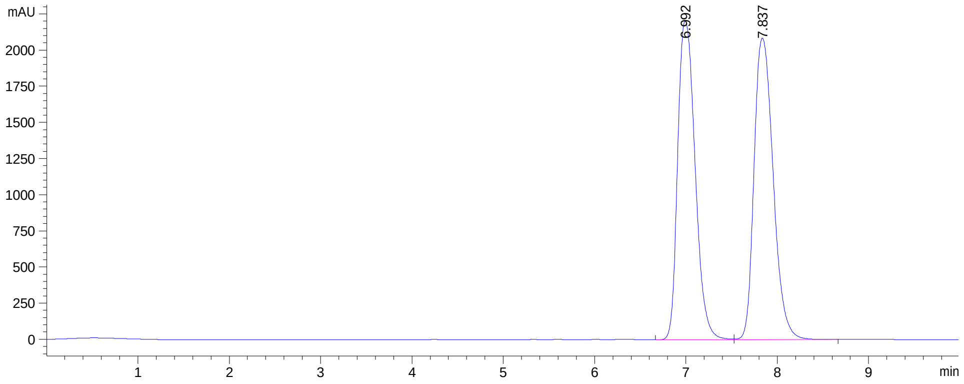

Signal 2: DAD1 C, Sig=210,8 Ref=360,100

Peak #	RetTime [min]	Type	Width [min]	Area [mAU*s]	Height [mAU]	Area %
1	6.992	BV	0.2103	2.92045e4	2207.74902	49.0784
2	7.837	VB	0.2295	3.03013e4	2085.24341	50.9216

Compound 4f

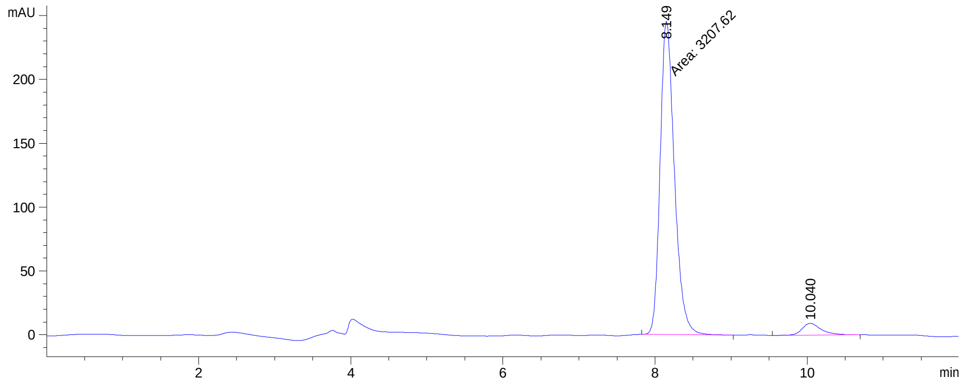

Signal 2: DAD1 C, Sig=210,4 Ref=off

Peak #	RetTime [min]	Type	Width [min]	Area [mAU*s]	Height [mAU]	Area %
1	8.149	MM	0.2178	3207.62305	245.47594	95.1295
2	10.040	BB	0.2632	164.22430	9.33874	4.8705

Racemic Mixture of Compound 4f

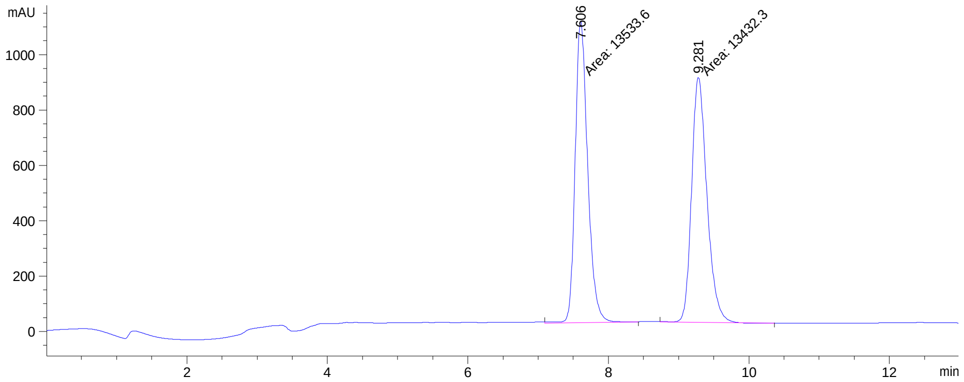

Signal 2: DAD1 C, Sig=210,8 Ref=360,100

Peak #	RetTime [min]	Type	Width [min]	Area [mAU*s]	Height [mAU]	Area %
1	7.606	MM	0.2068	1.35336e4	1090.91626	50.1879
2	9.281	MM	0.2530	1.34323e4	884.86627	49.8121

Compound 4g

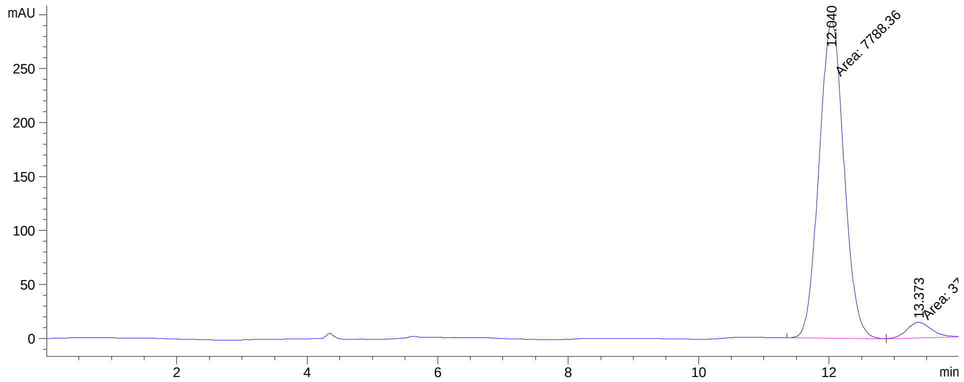

Signal 2: DAD1 C, Sig=210,8 Ref=360,100

Peak #	RetTime [min]	Type	Width [min]	Area [mAU*s]	Height [mAU]	Area %
1	12.040	MM	0.4420	7788.35742	293.66068	95.4123
2	13.373	MM	0.4345	374.48682	14.36588	4.5877

Racemic Mixture of Compound 4g

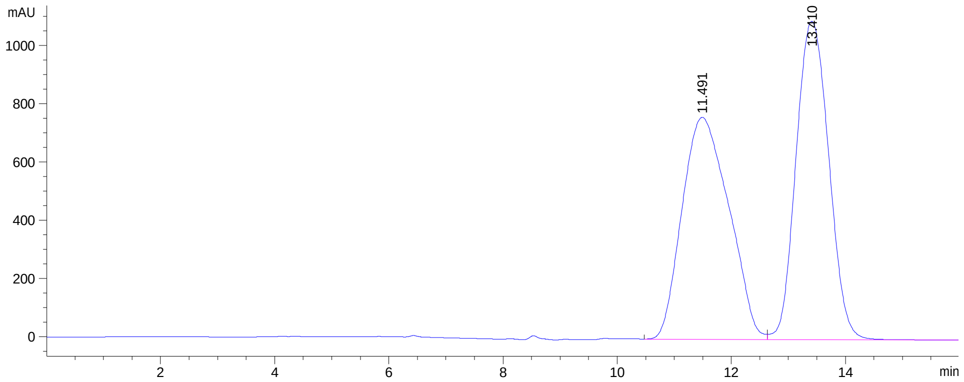

Signal 2: DAD1 C, Sig=210,8 Ref=360,100

Peak #	RetTime [min]	Type	Width [min]	Area [mAU*s]	Height [mAU]	Area %
1	11.491	BV	0.8829	4.34104e4	761.71118	49.8758
2	13.410	VBA	0.6438	4.36266e4	1092.70667	50.1242

Compound 4h

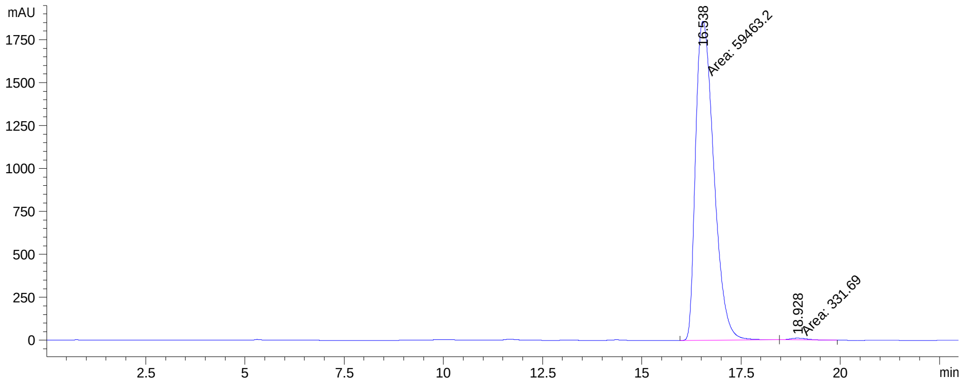

Signal 2: DAD1 C, Sig=210,4 Ref=off

Peak #	RetTime [min]	Type	Width [min]	Area [mAU*s]	Height [mAU]	Area %
1	16.538	MM	0.5334	5.94632e4	1858.05566	99.4453
2	18.928	MM	0.5223	331.68994	10.58490	0.5547

Racemic Mixture of Compound 4h

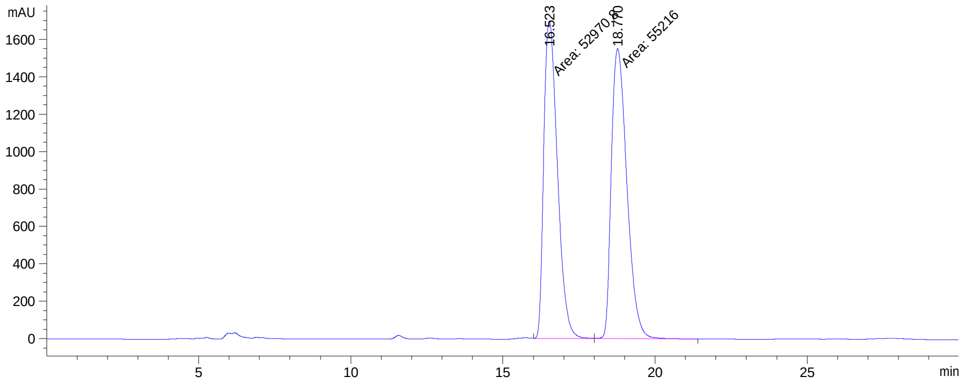

Signal 2: DAD1 C, Sig=210,4 Ref=off

Peak #	RetTime [min]	Type	Width [min]	Area [mAU*s]	Height [mAU]	Area %
1	16.523	MF	0.5201	5.29707e4	1697.43408	48.9623
2	18.770	FM	0.5934	5.52160e4	1550.71252	51.0377

Compound 4i

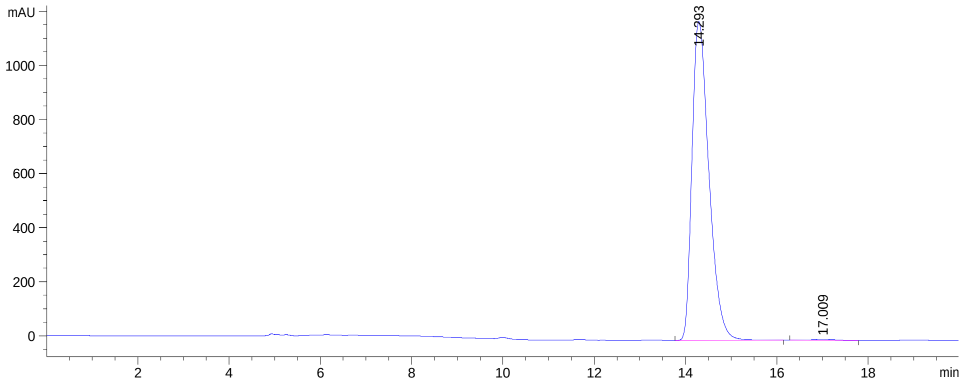

Signal 2: DAD1 C, Sig=210,4 Ref=off

Peak #	RetTime [min]	Type	Width [min]	Area [mAU*s]	Height [mAU]	Area %
1	14.293	BB	0.3988	3.04383e4	1180.39001	99.5751
2	17.009	BB	0.4115	129.88663	4.31160	0.4249

Racemic Mixture of Compound 4i

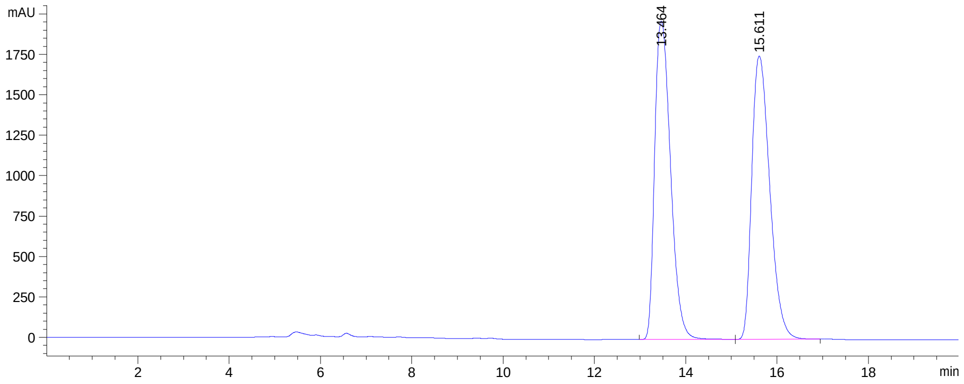

Signal 2: DAD1 C, Sig=210,8 Ref=360,100

Peak #	RetTime [min]	Type	Width [min]	Area [mAU*s]	Height [mAU]	Area %
1	13.464	BB	0.3797	4.75797e4	1970.71460	49.7990
2	15.611	BB	0.4312	4.79637e4	1752.90698	50.2010

Compound 4j

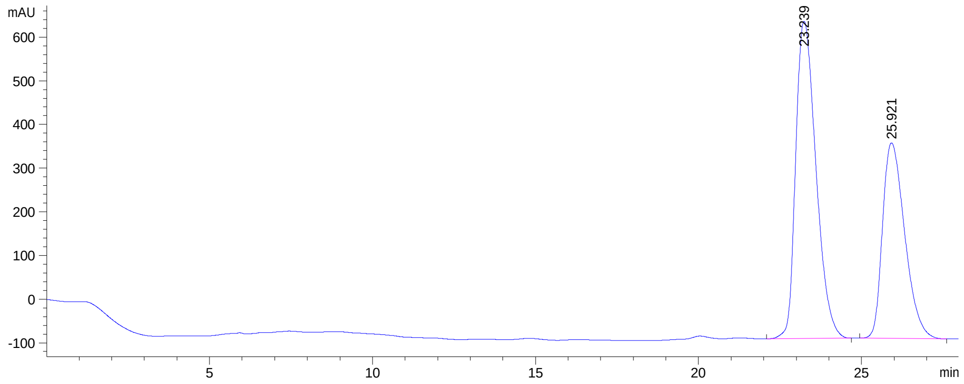

Signal 2: DAD1 C, Sig=210,4 Ref=off

Peak #	RetTime [min]	Type	Width [min]	Area [mAU*s]	Height [mAU]	Area %
1	23.239	BB	0.6808	3.14259e4	726.36035	59.6831
2	25.921	BBA	0.7454	2.12287e4	447.52734	40.3169

Racemic Mixture of Compound 4j

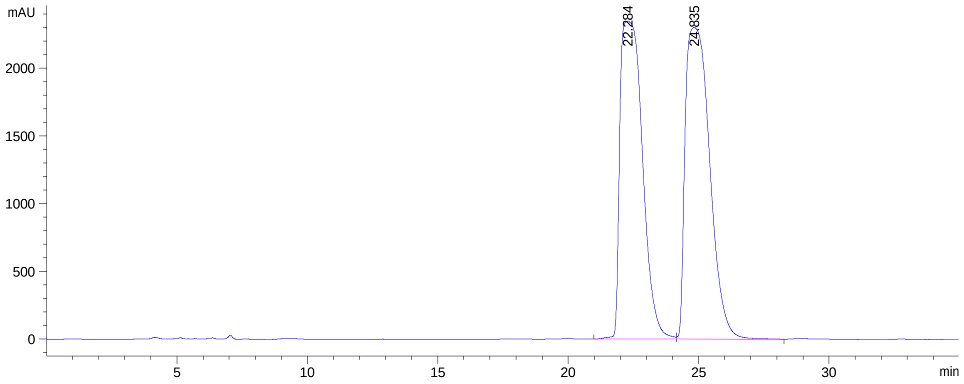

Signal 2: DAD1 C, Sig=210,4 Ref=off

Peak #	RetTime [min]	Type	Width [min]	Area [mAU*s]	Height [mAU]	Area %
1	22.284	BV	0.7066	1.40621e5	2348.28906	48.4212
2	24.835	VB	0.7912	1.49791e5	2301.83057	51.5788

Compound 4k

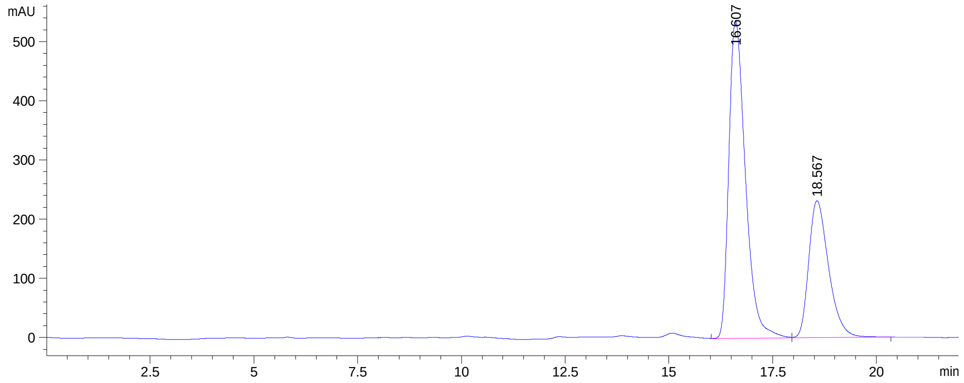


```
Signal 2: DAD1 C, Sig=210,4 Ref=off

Peak RetTime Type  Width     Area      Height     Area
  #   [min]        [min]   [mAU*s]     [mAU]        %
----|-------|----|-------|----------|----------|--------|
   1  16.607 BV    0.4254 1.49888e4  537.32788  66.1111
   2  18.567 VB    0.5029 7683.33398  231.67343  33.8889
```

Racemic Mixture of Compound 4k

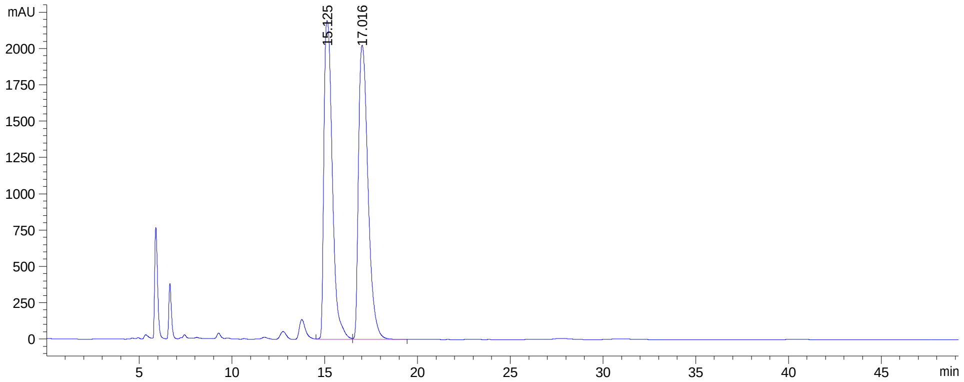


```
Signal 2: DAD1 C, Sig=210,8 Ref=360,100

Peak RetTime Type  Width     Area      Height     Area
  #   [min]        [min]   [mAU*s]     [mAU]        %
----|-------|----|-------|----------|----------|--------|
   1  15.125 VV    0.4966 6.90311e4 2196.82446  48.9150
   2  17.016 VB    0.5629 7.20934e4 2027.36389  51.0850
```

Compound 4l

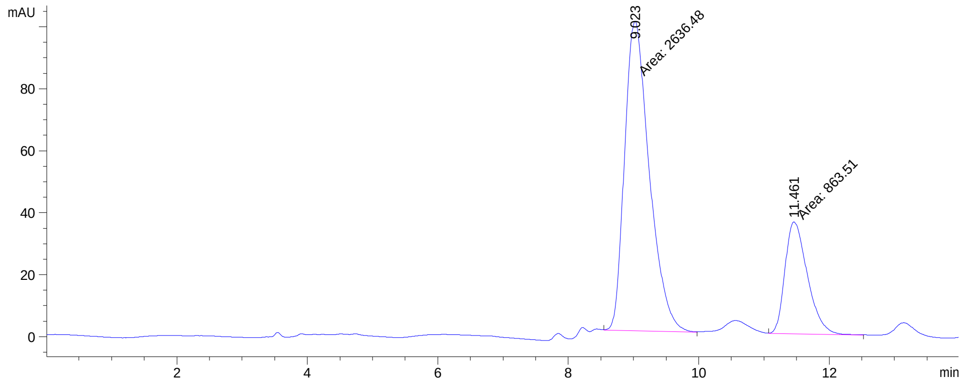

Signal 2: DAD1 C, Sig=210,8 Ref=360,100

Peak #	RetTime [min]	Type	Width [min]	Area [mAU*s]	Height [mAU]	Area %
1	9.023	MM	0.4397	2636.47778	99.93012	75.3282
2	11.461	MM	0.3986	863.51044	36.10436	24.6718

Racemic Mixture of Compound 4l

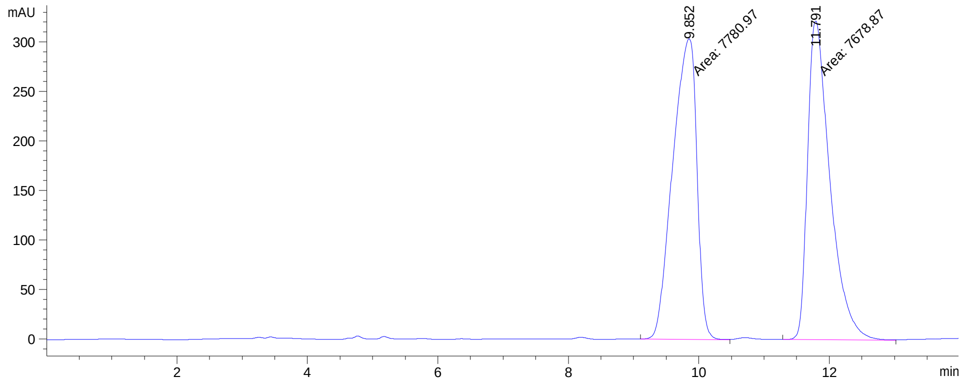

Signal 2: DAD1 C, Sig=210,8 Ref=360,100

Peak #	RetTime [min]	Type	Width [min]	Area [mAU*s]	Height [mAU]	Area %
1	9.852	MM	0.4271	7780.96924	303.62354	50.3302
2	11.791	MM	0.3976	7678.87158	321.88437	49.6698

Compound 4m

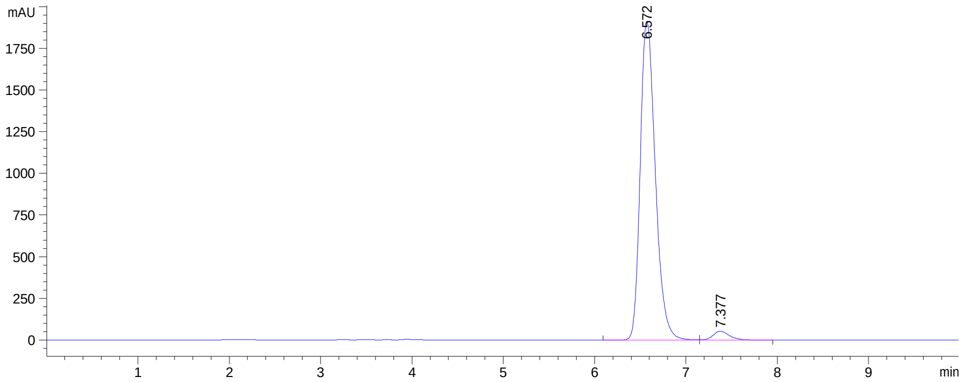

Signal 2: DAD1 C, Sig=210,8 Ref=360,100

Peak #	RetTime [min]	Type	Width [min]	Area [mAU*s]	Height [mAU]	Area %
1	6.572	BV	0.1761	2.19966e4	1914.24597	96.9945
2	7.377	VB	0.1927	681.58624	53.46494	3.0055

Racemic Mixture of Compound 4m

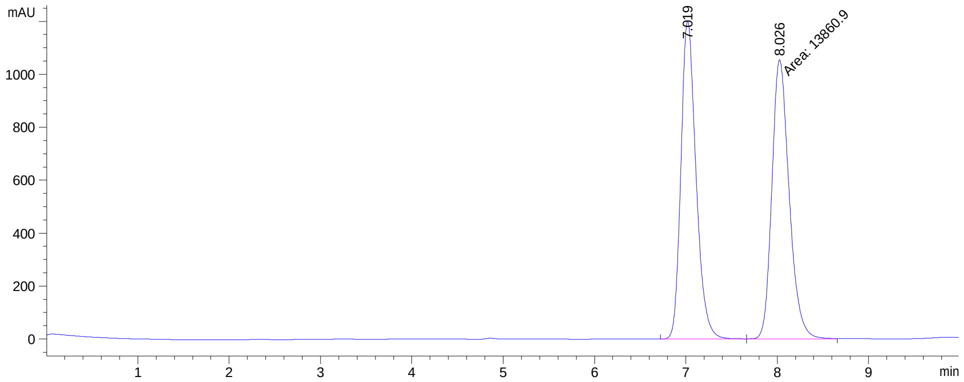

Signal 2: DAD1 C, Sig=210,8 Ref=360,100

Peak #	RetTime [min]	Type	Width [min]	Area [mAU*s]	Height [mAU]	Area %
1	7.019	BB	0.1759	1.37908e4	1201.39502	49.8732
2	8.026	MF	0.2192	1.38609e4	1053.85059	50.1268

Compound 4n

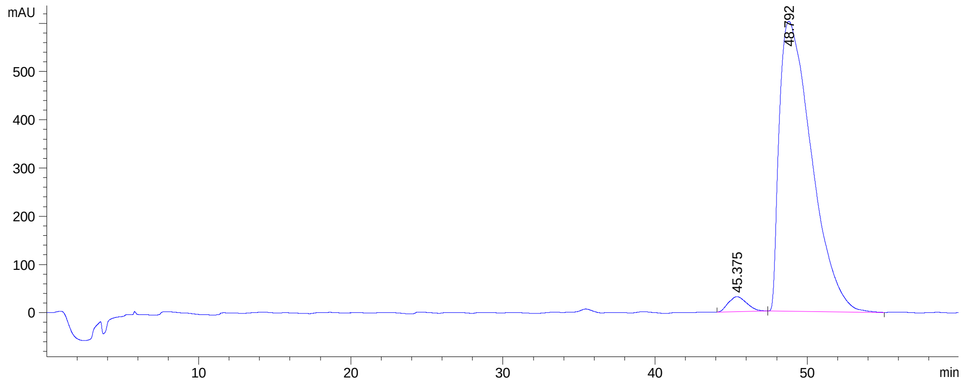

Signal 2: DAD1 C, Sig=210,4 Ref=off

Peak #	RetTime [min]	Type	Width [min]	Area [mAU*s]	Height [mAU]	Area %
1	45.375	BB	1.0949	2619.74829	31.15900	2.8752
2	48.792	BB	2.0607	8.84965e4	601.41272	97.1248

Racemic Mixture of Compound 4n

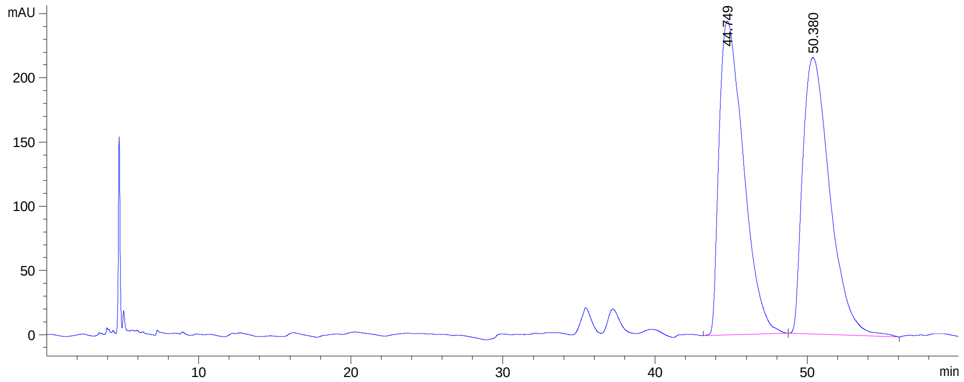

Signal 2: DAD1 C, Sig=210,4 Ref=off

Peak #	RetTime [min]	Type	Width [min]	Area [mAU*s]	Height [mAU]	Area %
1	44.749	BB	1.6115	2.72805e4	244.53568	50.5396
2	50.380	BB	1.8663	2.66980e4	215.36464	49.4604

Compound 4o

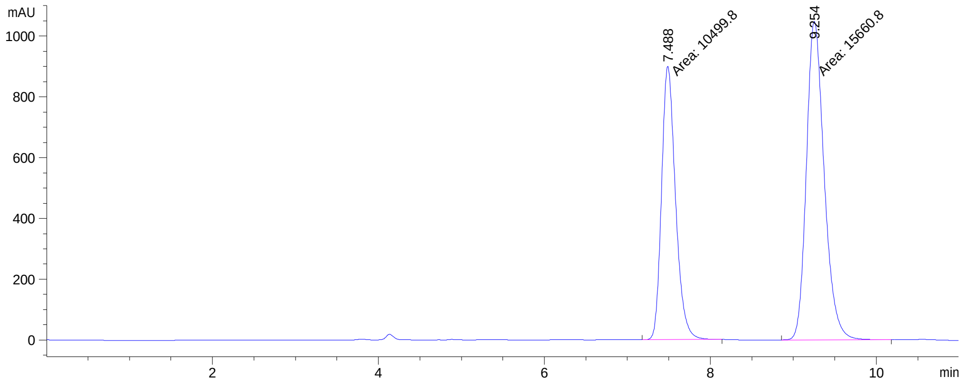

Signal 2: DAD1 C, Sig=210,4 Ref=off

Peak #	RetTime [min]	Type	Width [min]	Area [mAU*s]	Height [mAU]	Area %
1	7.488	MM	0.1945	1.04998e4	899.71893	40.1361
2	9.254	MM	0.2490	1.56608e4	1048.03430	59.8639

Racemic Mixture of Compound 4o

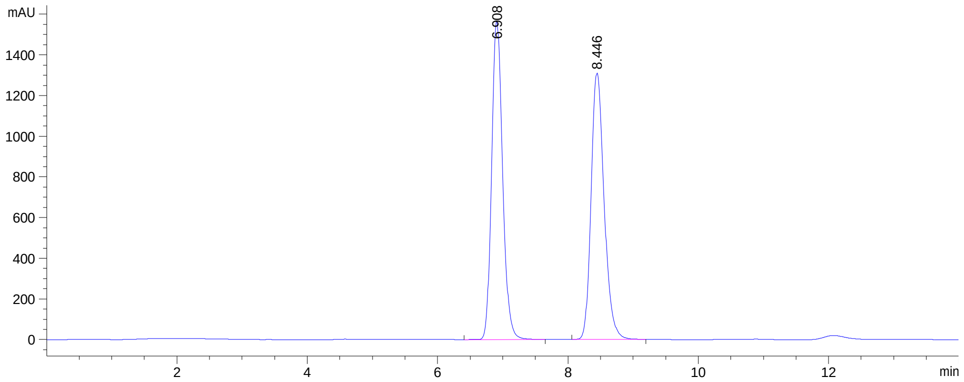

Signal 2: DAD1 C, Sig=210,8 Ref=360,100

Peak #	RetTime [min]	Type	Width [min]	Area [mAU*s]	Height [mAU]	Area %
1	6.908	BB	0.1786	1.83457e4	1566.84119	50.2629
2	8.446	BB	0.2118	1.81538e4	1308.60864	49.7371

Compound 4p

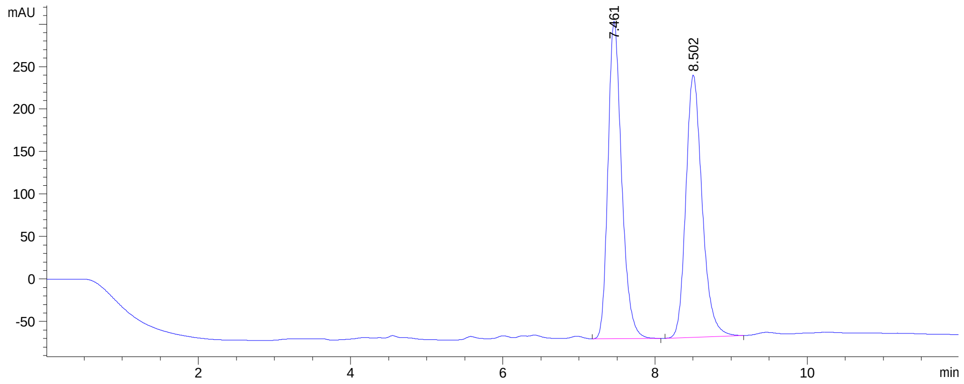

Signal 2: DAD1 C, Sig=210,4 Ref=off

Peak #	RetTime [min]	Type	Width [min]	Area [mAU*s]	Height [mAU]	Area %
1	7.461	BB	0.1897	4668.28418	373.83041	50.4566
2	8.502	BB	0.2253	4583.79053	308.58939	49.5434

Racemic Mixture of Compound 4p

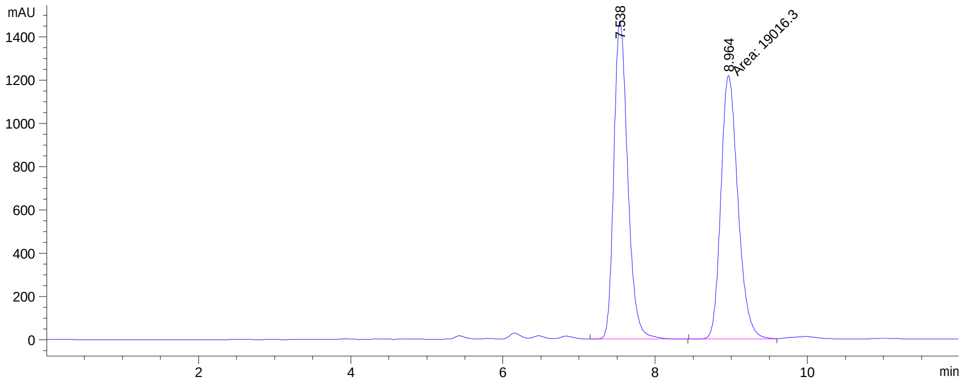

Signal 2: DAD1 C, Sig=210,8 Ref=360,100

Peak #	RetTime [min]	Type	Width [min]	Area [mAU*s]	Height [mAU]	Area %
1	7.538	BB	0.1963	1.86813e4	1469.87732	49.5557
2	8.964	MF	0.2603	1.90163e4	1217.37024	50.4443

Compound 4q

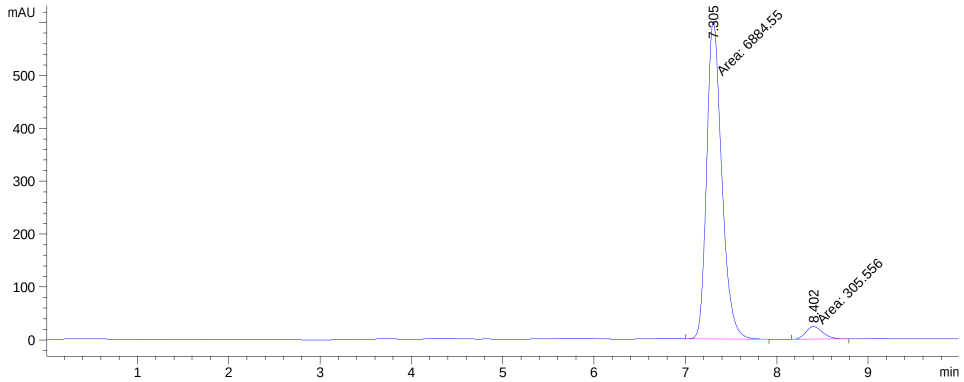


```
Signal 2: DAD1 C, Sig=210,4 Ref=off

Peak RetTime Type  Width     Area      Height     Area
  #   [min]        [min]   [mAU*s]     [mAU]        %
----|-------|----|-------|----------|----------|--------|
   1   7.305 MM    0.1910 6884.54639  600.80090  95.7503
   2   8.402 MM    0.2139  305.55585   23.80785   4.2497
```

Racemic Mixture of Compound 4q

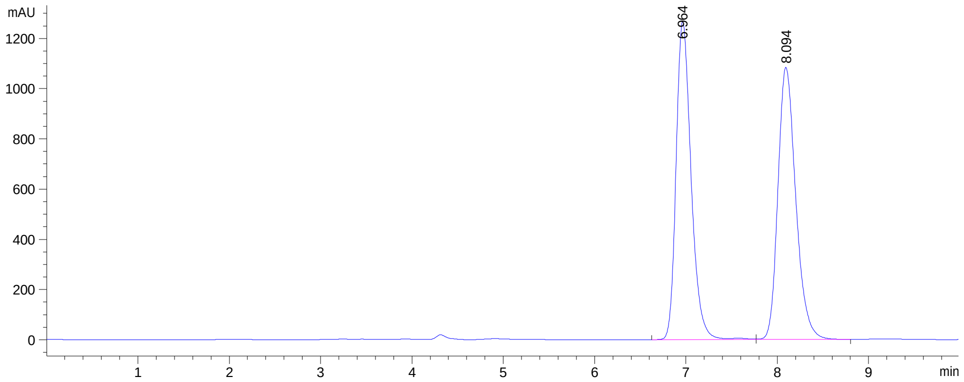


```
Signal 2: DAD1 C, Sig=210,8 Ref=360,100

Peak RetTime Type  Width     Area      Height     Area
  #   [min]        [min]   [mAU*s]     [mAU]        %
----|-------|----|-------|----------|----------|--------|
   1   6.964 BV R  0.1744 1.44881e4 1269.59119  49.6353
   2   8.094 VB    0.2082 1.47010e4 1083.85522  50.3647
```

Compound 4r

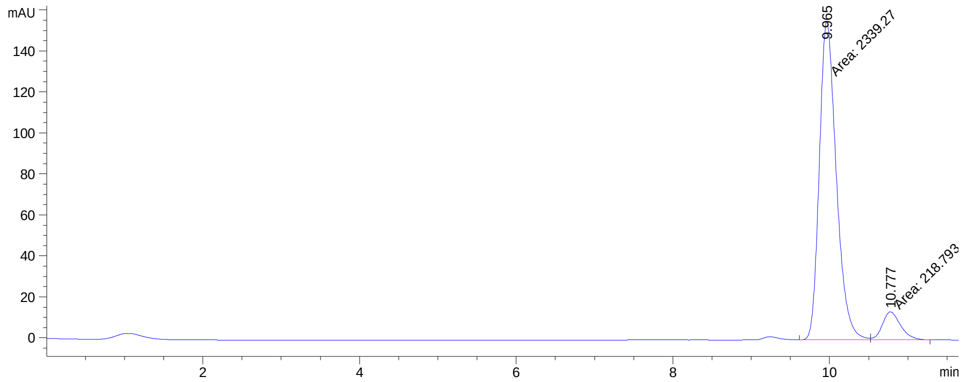

Signal 3: DAD1 D, Sig=230,4 Ref=off

Peak #	RetTime [min]	Type	Width [min]	Area [mAU*s]	Height [mAU]	Area %
1	9.965	MF	0.2508	2339.27319	155.43924	91.4469
2	10.777	FM	0.2674	218.79314	13.63477	8.5531

Totals : 2558.06633 169.07401

Racemic Mixture of Compound 4r

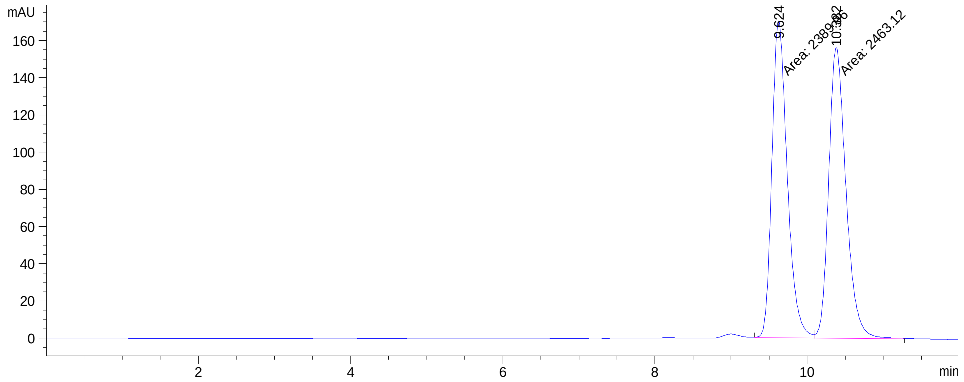

Signal 3: DAD1 D, Sig=230,16 Ref=360,100

Peak #	RetTime [min]	Type	Width [min]	Area [mAU*s]	Height [mAU]	Area %
1	9.624	MF	0.2337	2389.95654	170.45605	49.2462
2	10.382	FM	0.2627	2463.12012	156.27467	50.7538

Totals : 4853.07666 326.73073

Compound 4s

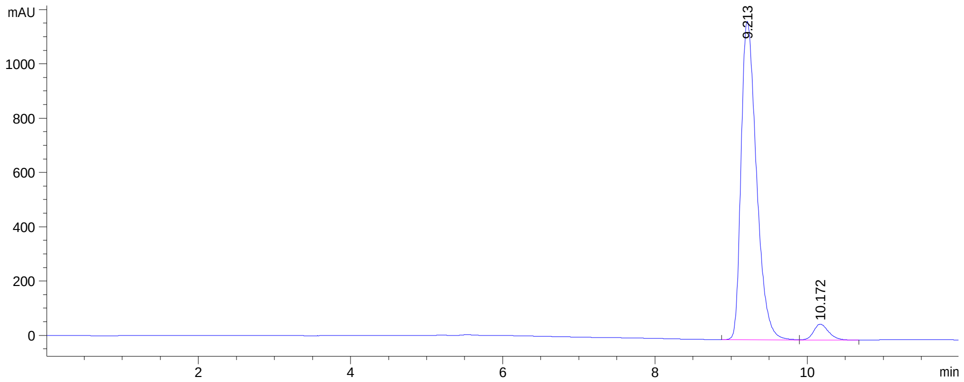

Signal 2: DAD1 C, Sig=210,4 Ref=off

Peak #	RetTime [min]	Type	Width [min]	Area [mAU*s]	Height [mAU]	Area %
1	9.213	BV	0.2176	1.64500e4	1172.83899	95.3531
2	10.172	VB	0.2098	801.66211	58.52592	4.6469

Racemic Mixture of Compound 4s

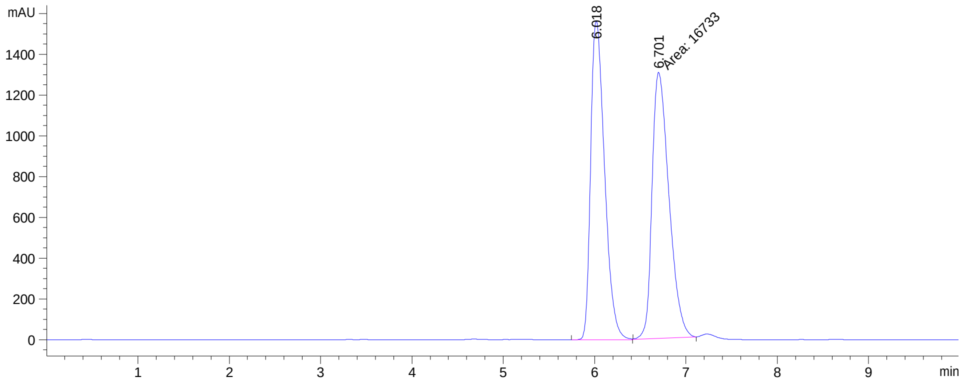

Signal 2: DAD1 C, Sig=210,8 Ref=360,100

Peak #	RetTime [min]	Type	Width [min]	Area [mAU*s]	Height [mAU]	Area %
1	6.018	BV	0.1654	1.65426e4	1564.04810	49.7138
2	6.701	MM	0.2137	1.67330e4	1305.16809	50.2862

Compound 4t

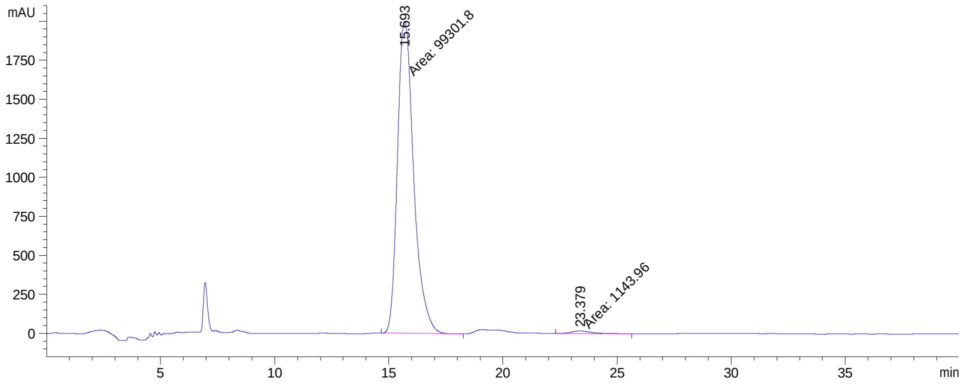

Signal 2: DAD1 C, Sig=210,4 Ref=off

Peak #	RetTime [min]	Type	Width [min]	Area [mAU*s]	Height [mAU]	Area %
1	15.693	MM	0.8274	9.93018e4	2000.24329	98.8611
2	23.379	MM	1.0836	1143.95984	17.59476	1.1389

Racemic Mixture of Compound 4t

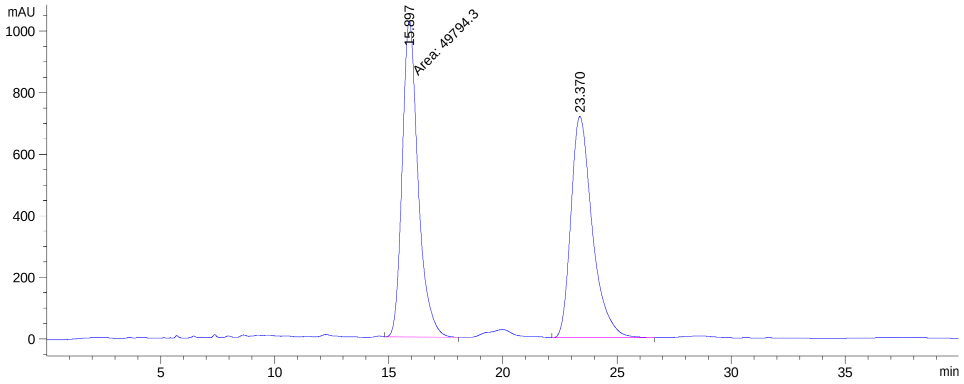

Signal 2: DAD1 C, Sig=210,4 Ref=off

Peak #	RetTime [min]	Type	Width [min]	Area [mAU*s]	Height [mAU]	Area %
1	15.897	MM	0.8071	4.97943e4	1028.22571	51.4291
2	23.370	BB	0.9904	4.70269e4	719.64465	48.5709

Compound 4u

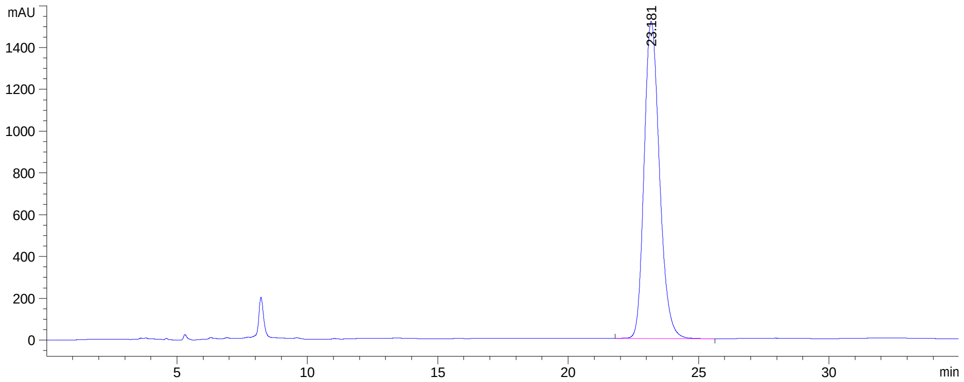

Signal 2: DAD1 C, Sig=210,4 Ref=off

Peak #	RetTime [min]	Type	Width [min]	Area [mAU*s]	Height [mAU]	Area %
1	23.181	BB	0.6346	6.22798e4	1518.22046	100.0000
Totals :				6.22798e4	1518.22046	

Racemic Mixture of Compound 4u

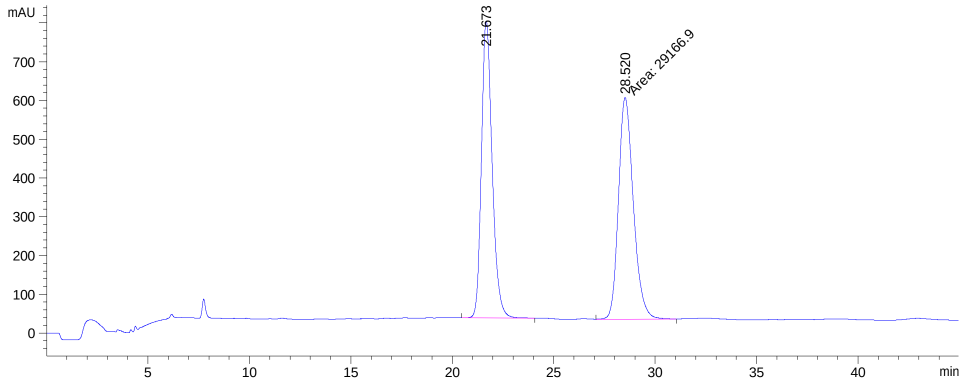

Signal 2: DAD1 C, Sig=210,8 Ref=360,100

Peak #	RetTime [min]	Type	Width [min]	Area [mAU*s]	Height [mAU]	Area %
1	21.673	BB	0.5613	2.81928e4	766.24414	49.1509
2	28.520	FM	0.8499	2.91669e4	571.95380	50.8491

Compound 6

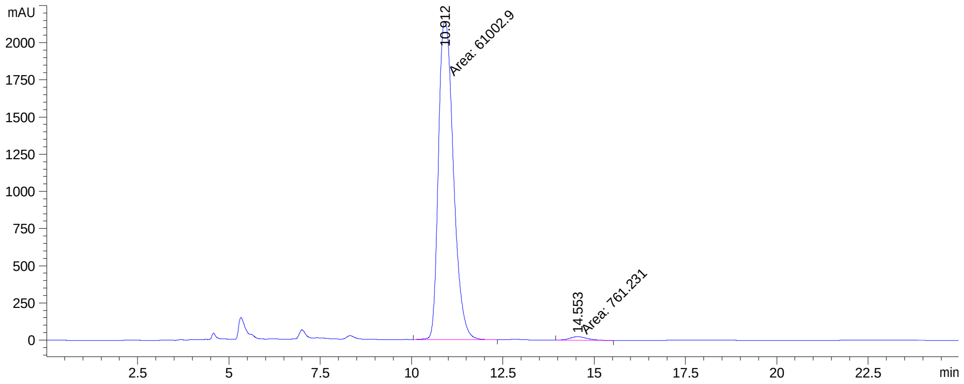

Signal 2: DAD1 C, Sig=210,4 Ref=off

Peak #	RetTime [min]	Type	Width [min]	Area [mAU*s]	Height [mAU]	Area %
1	10.912	MM	0.4749	6.10029e4	2141.04565	98.7675
2	14.553	MM	0.5438	761.23065	23.32891	1.2325

Racemic Mixture of Compound 6

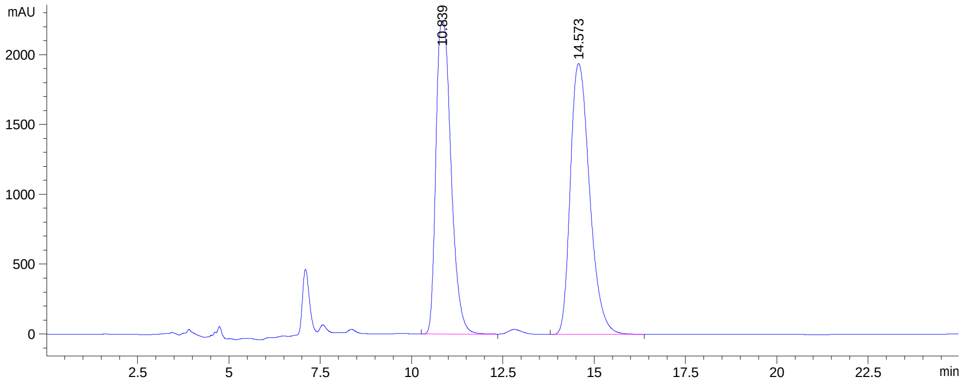

Signal 2: DAD1 C, Sig=210,4 Ref=off

Peak #	RetTime [min]	Type	Width [min]	Area [mAU*s]	Height [mAU]	Area %
1	10.839	BV	0.4492	6.45340e4	2246.09180	47.5638
2	14.573	BB	0.5718	7.11450e4	1940.00146	52.4362

Compound 8

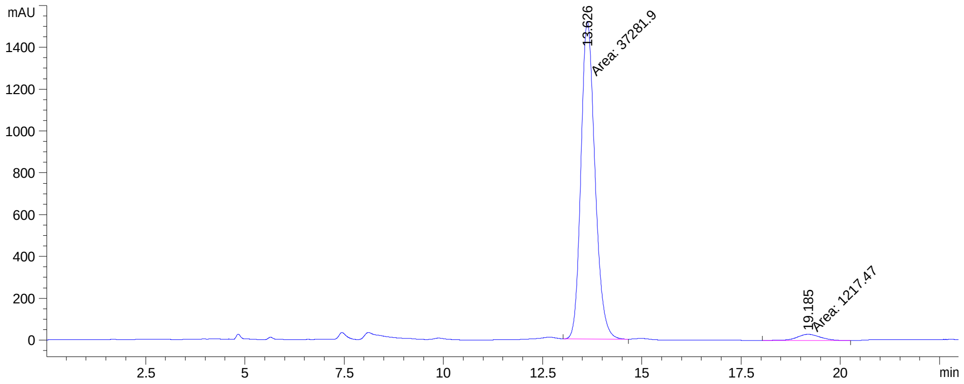

Signal 2: DAD1 C, Sig=210,4 Ref=off

Peak #	RetTime [min]	Type	Width [min]	Area [mAU*s]	Height [mAU]	Area %
1	13.626	MM	0.4090	3.72819e4	1519.33691	96.8377
2	19.185	MM	0.6858	1217.47058	29.58760	3.1623

Racemic Mixture of Compound 8

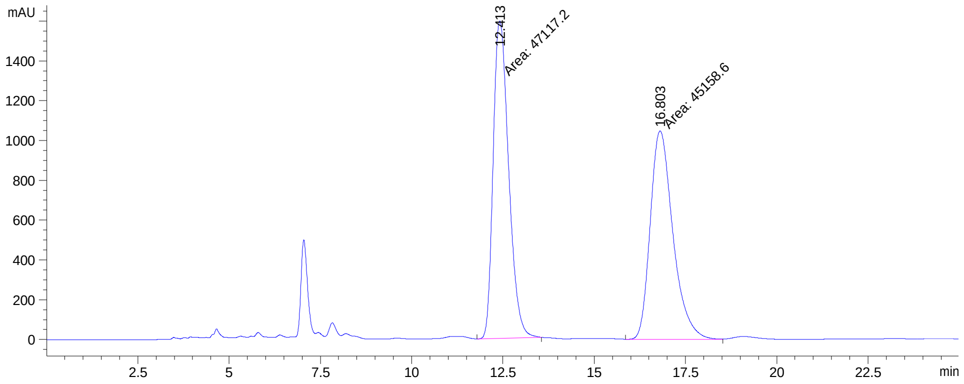

Signal 2: DAD1 C, Sig=210,4 Ref=off

Peak #	RetTime [min]	Type	Width [min]	Area [mAU*s]	Height [mAU]	Area %
1	12.413	MM	0.4924	4.71172e4	1594.83862	51.0613
2	16.803	MM	0.7193	4.51586e4	1046.31934	48.9387

5 NMR Data

Compound 2h

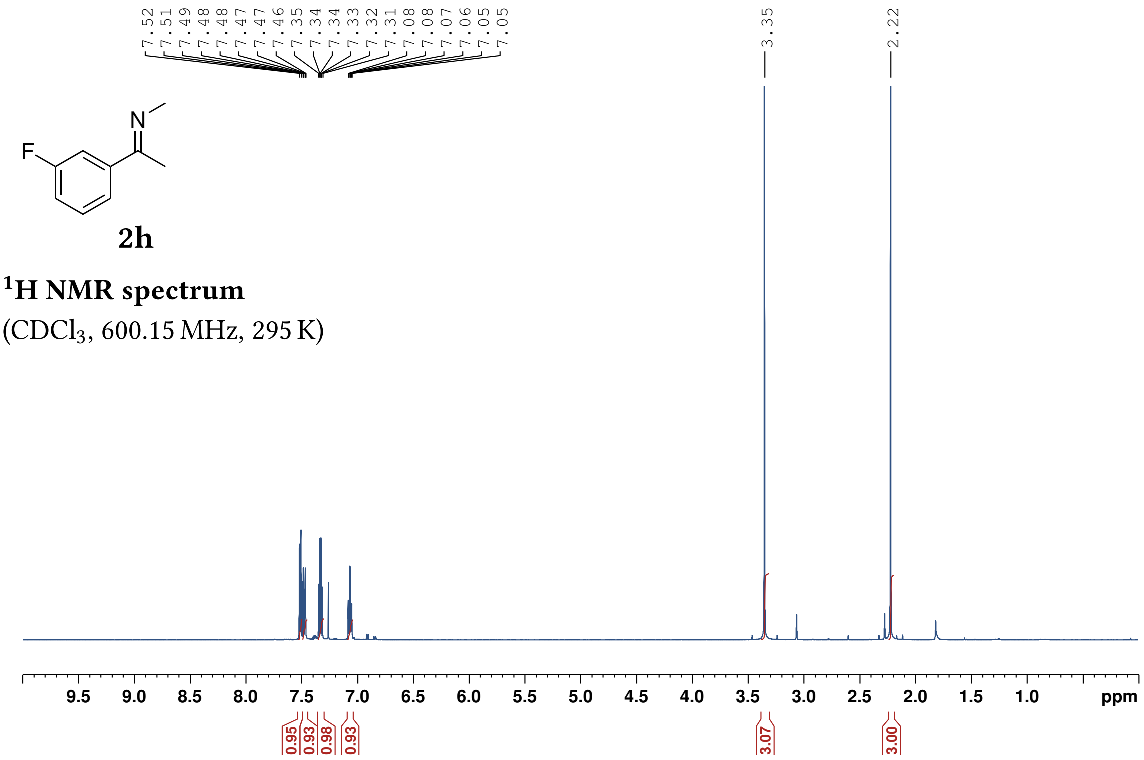

2h

^{1}H NMR spectrum

($CDCl_3$, 600.15 MHz, 295 K)

2h

^{13}C NMR spectrum

($CDCl_3$, 150.90 MHz, 295 K)

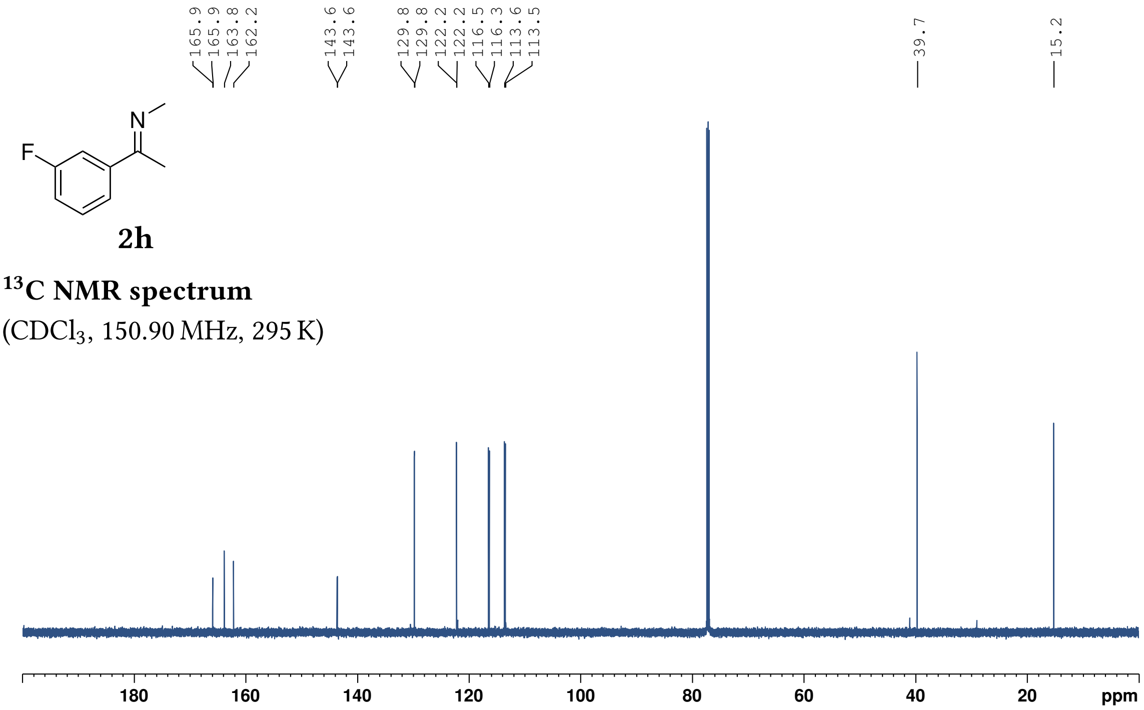

2h

^{19}F NMR spectrum

($CDCl_3$, 376.27 MHz, 295 K)

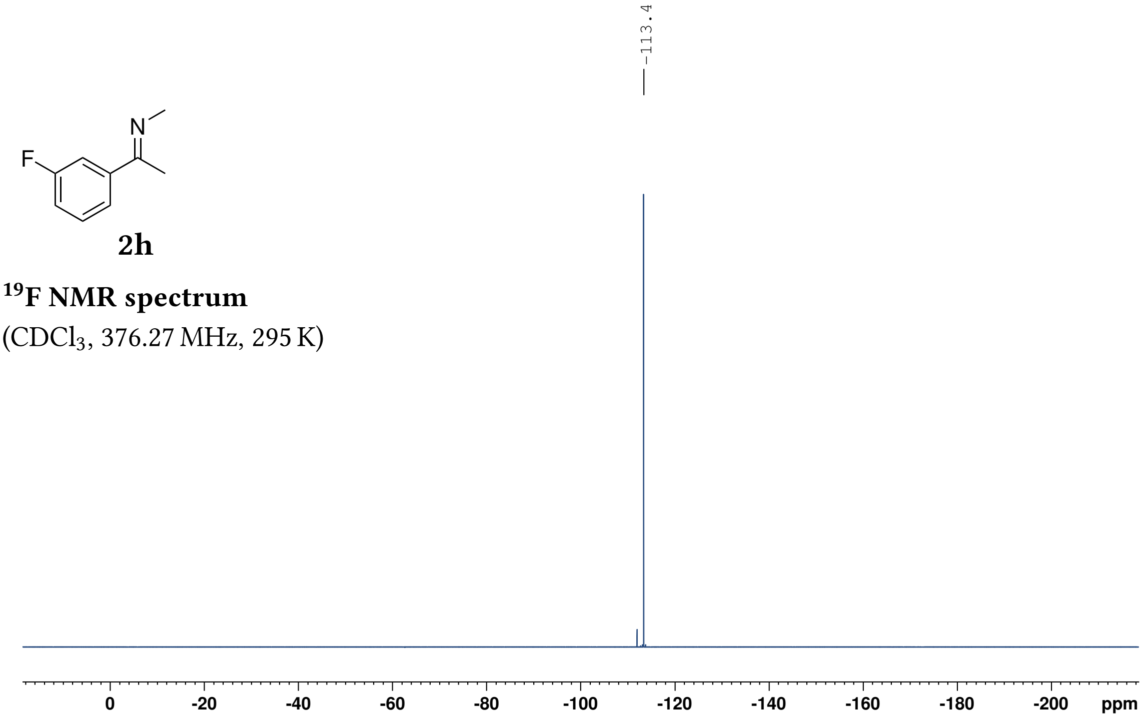

Compound 2n

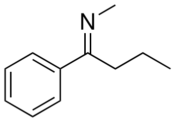

2n

^{1}H NMR spectrum

($CDCl_3$, 600.15 MHz, 295 K)

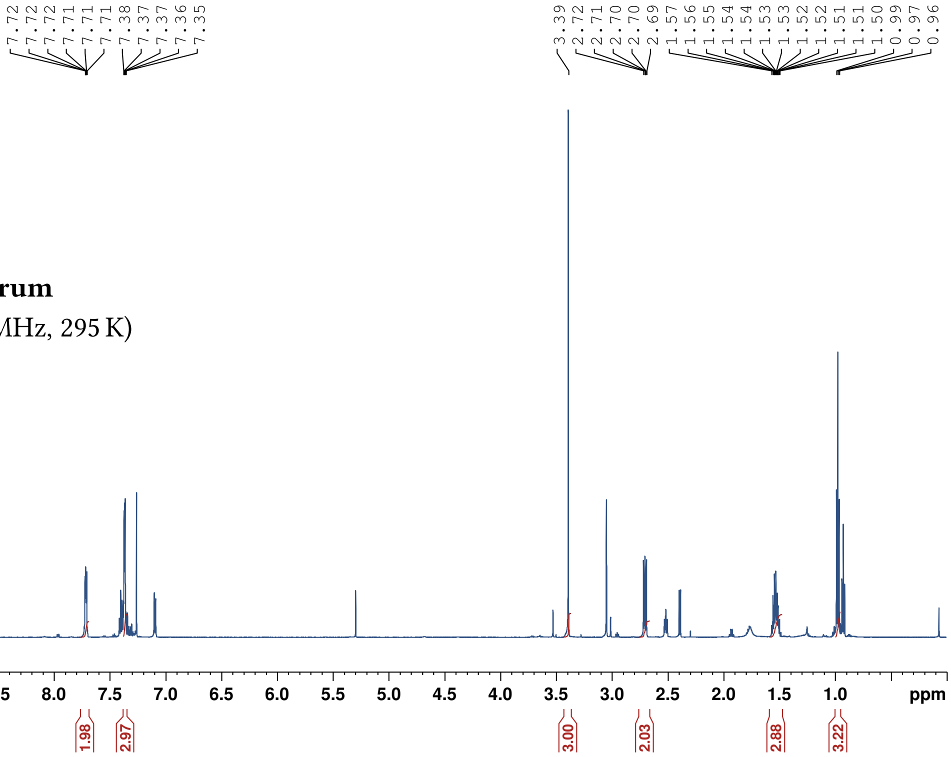

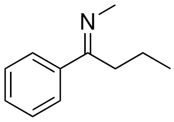

2n

^{13}C NMR spectrum

($CDCl_3$, 150.90 MHz, 295 K)

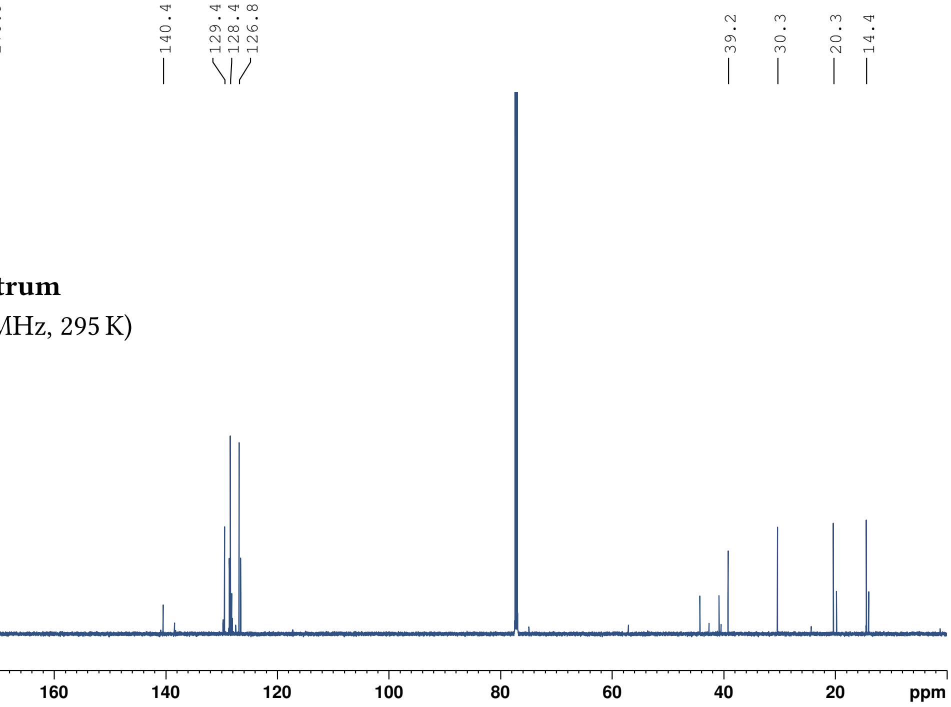

Compound 2r

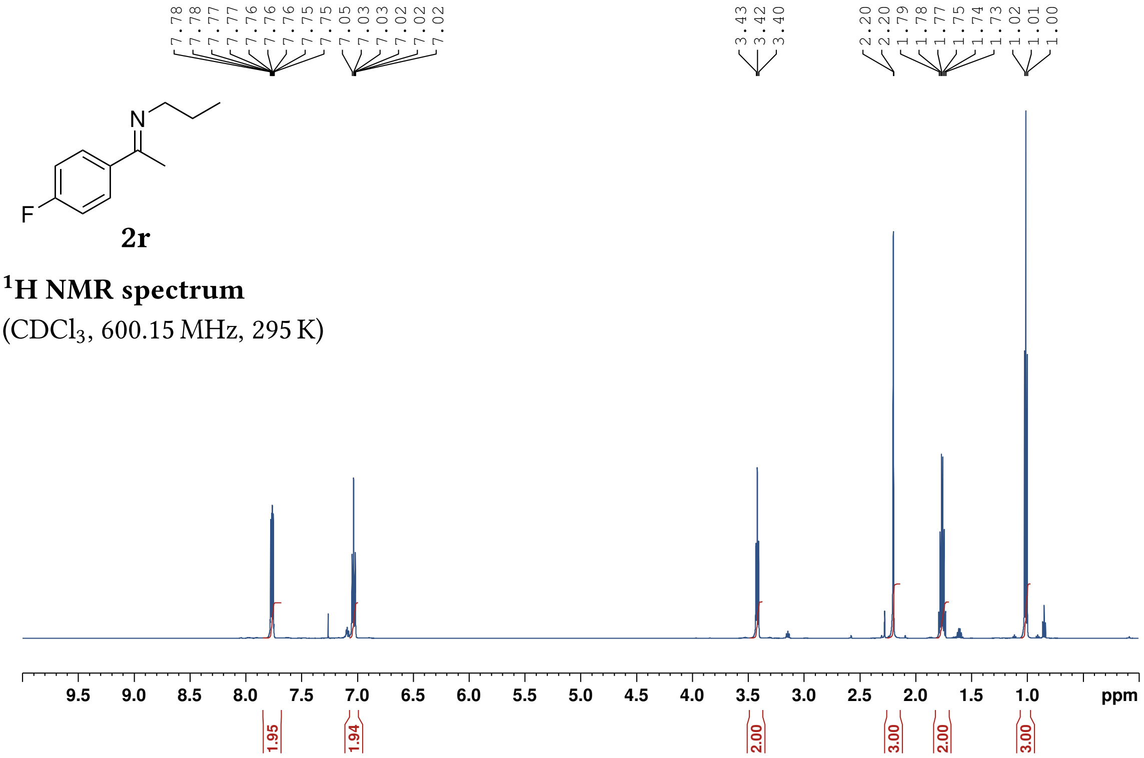

^{1}H NMR spectrum
($CDCl_3$, 600.15 MHz, 295 K)

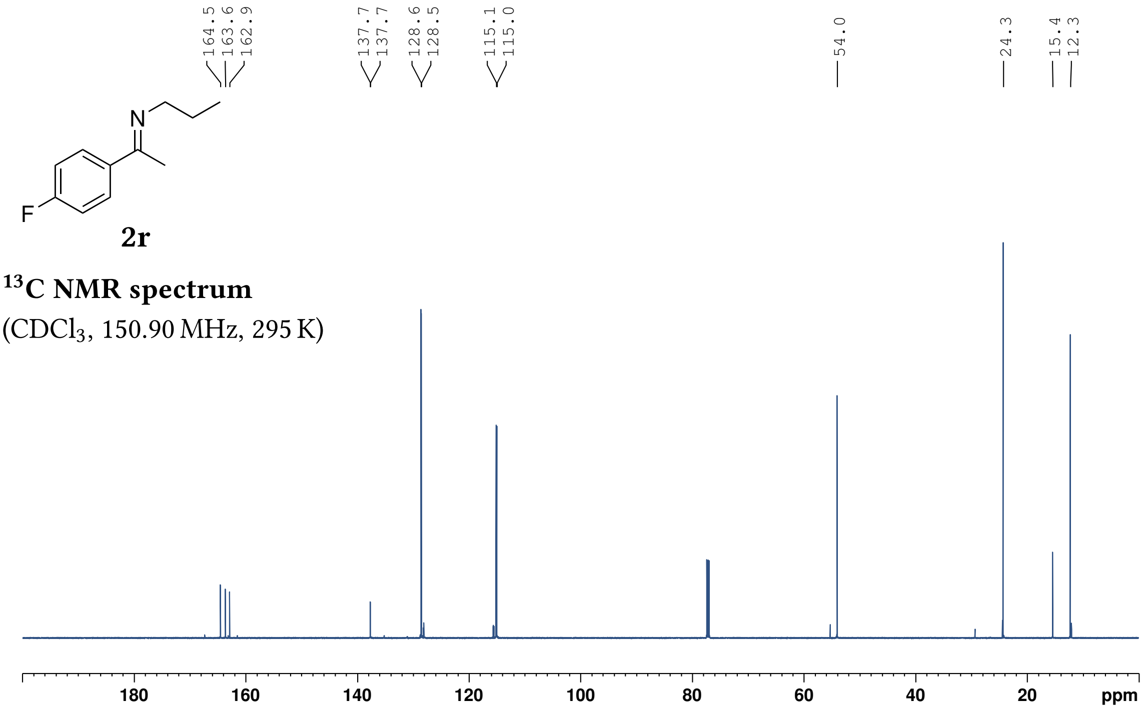

^{13}C NMR spectrum
($CDCl_3$, 150.90 MHz, 295 K)

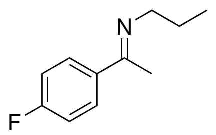

2r

^{19}F NMR spectrum

($CDCl_3$, 376.27 MHz, 295 K)

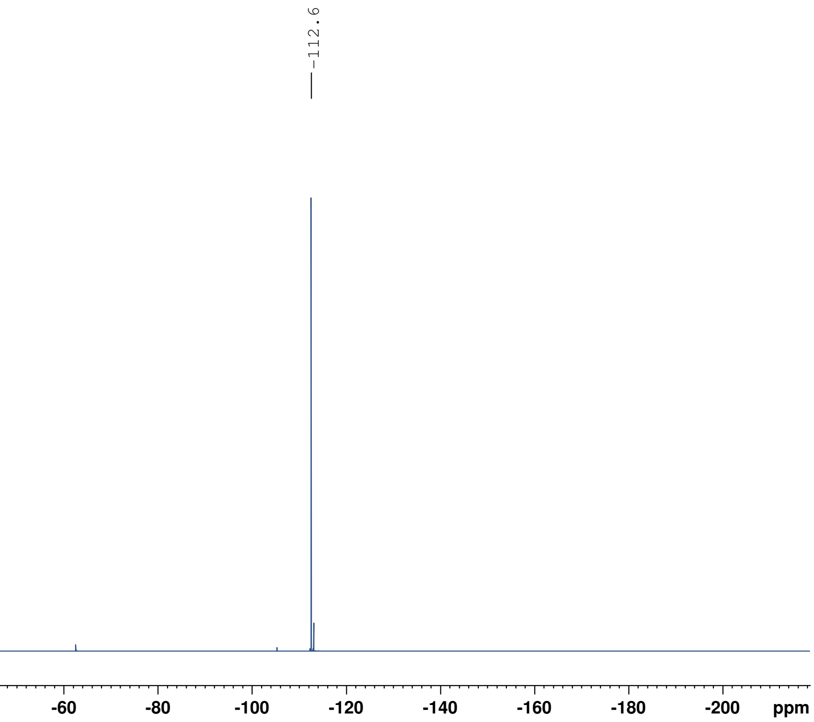

Compound 2s

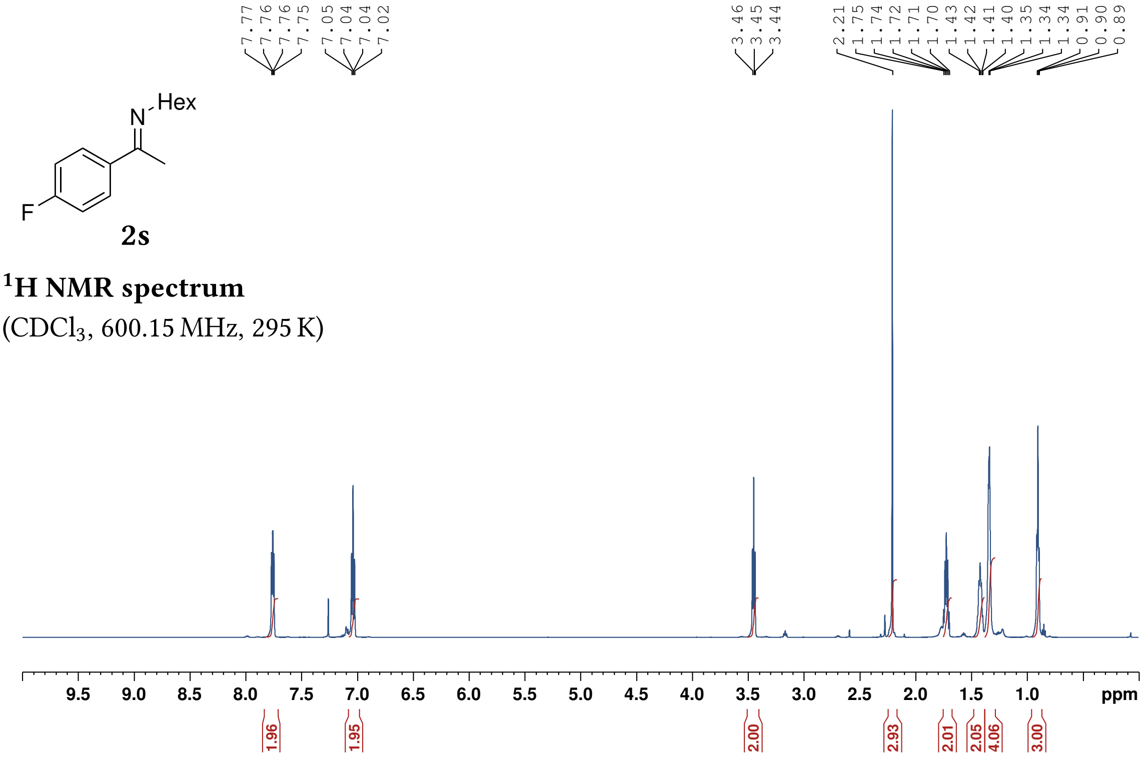

^{1}H NMR spectrum
($CDCl_3$, 600.15 MHz, 295 K)

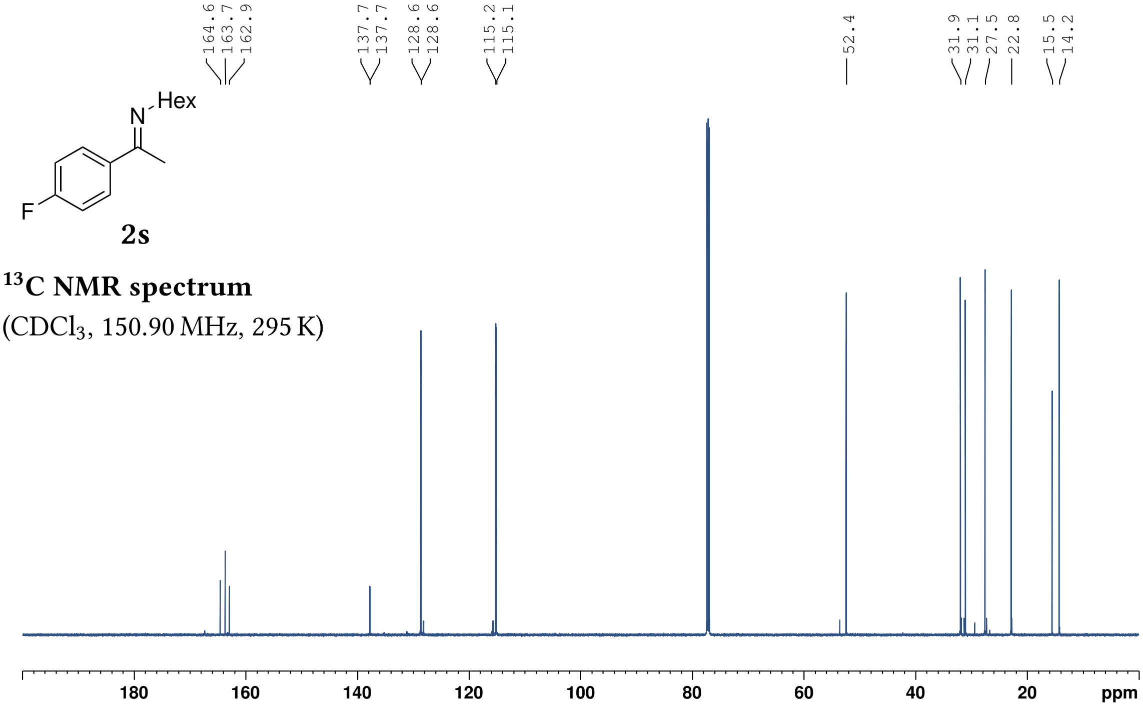

^{13}C NMR spectrum
($CDCl_3$, 150.90 MHz, 295 K)

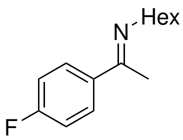

2s

^{19}F NMR spectrum
($CDCl_3$, 376.27 MHz, 295 K)

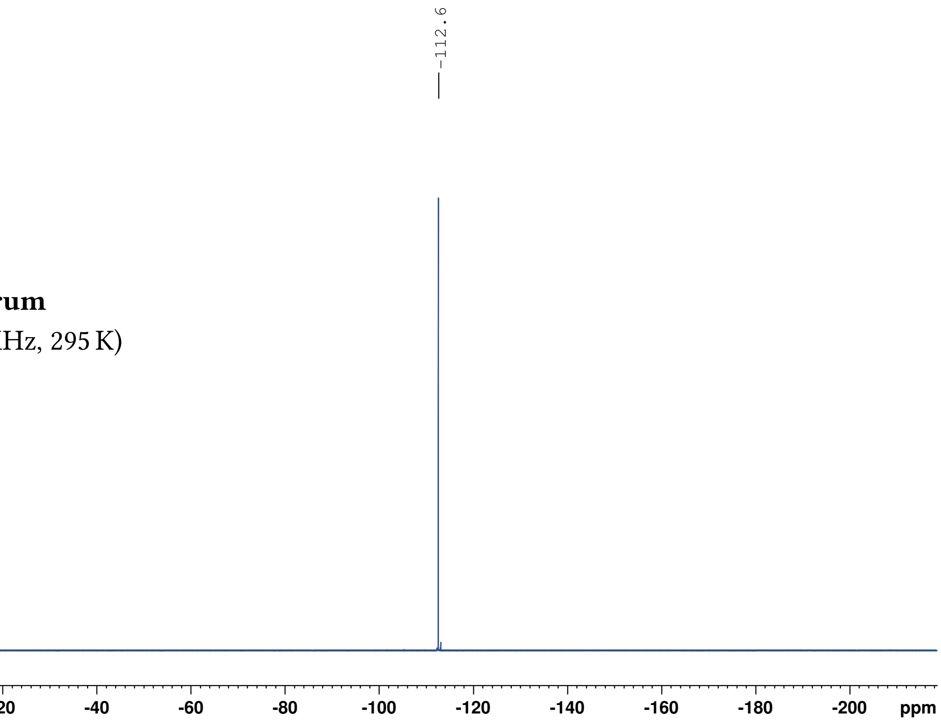

Compound 2u

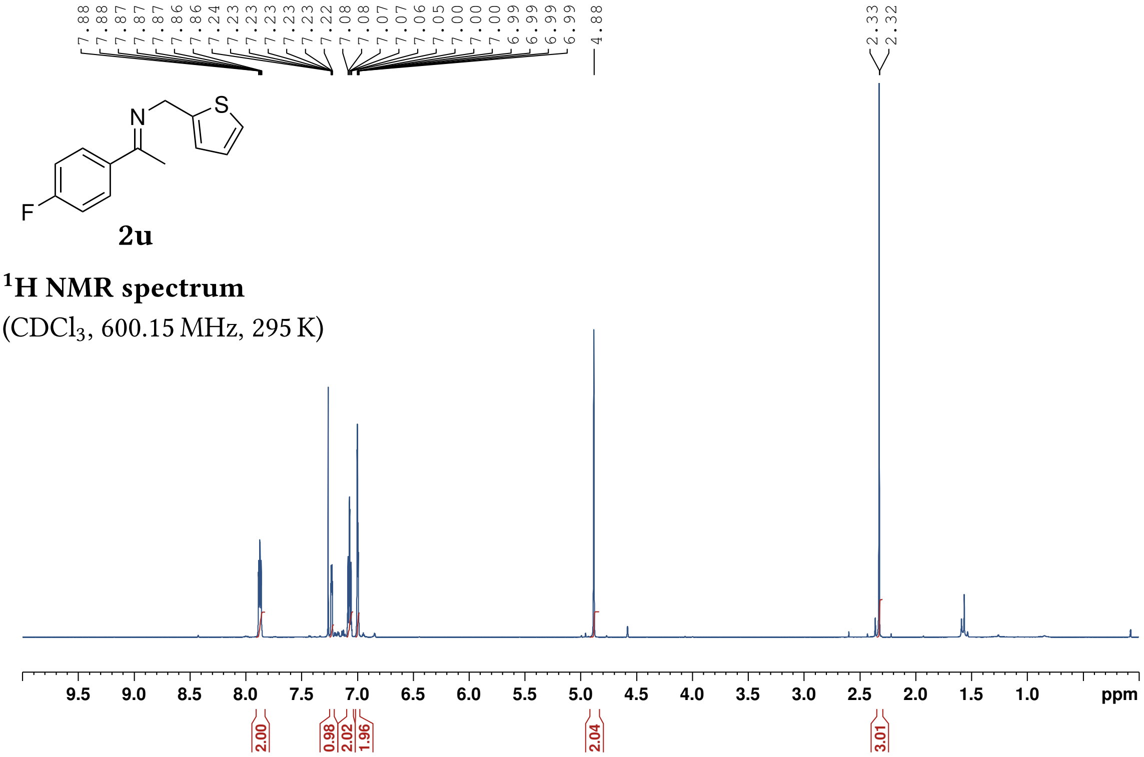

2u

^{1}H NMR spectrum

($CDCl_3$, 600.15 MHz, 295 K)

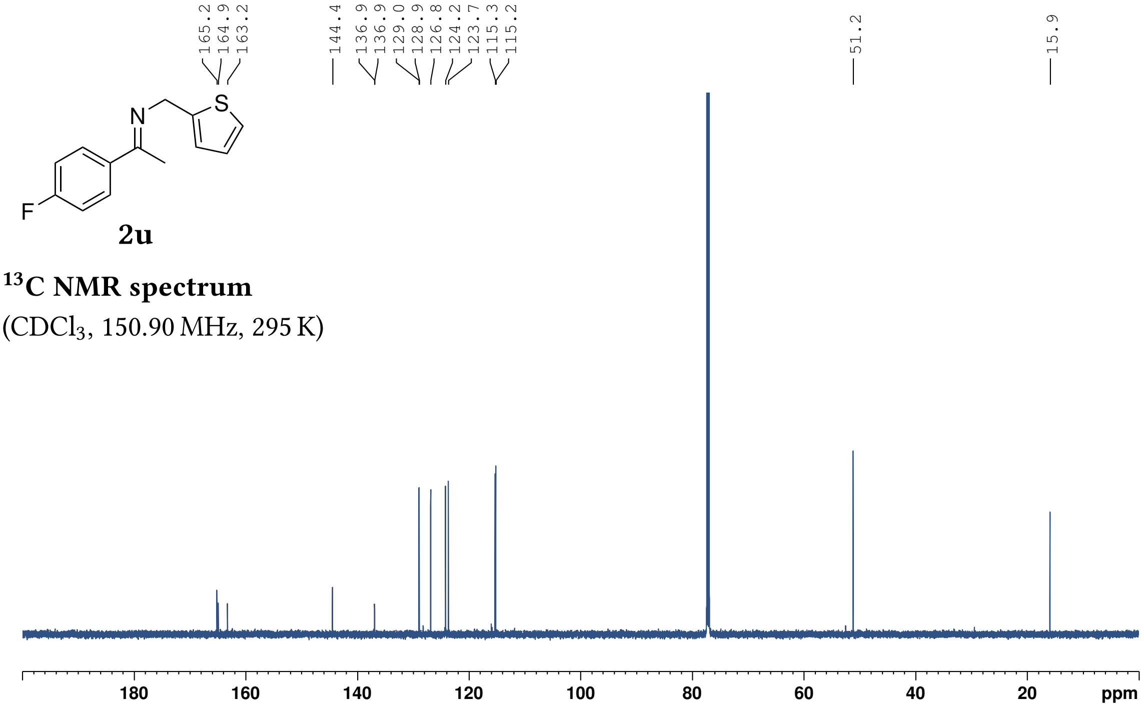

2u

^{13}C NMR spectrum

($CDCl_3$, 150.90 MHz, 295 K)

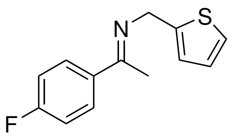

2u

^{19}F NMR spectrum

($CDCl_3$, 376.27 MHz, 295 K)

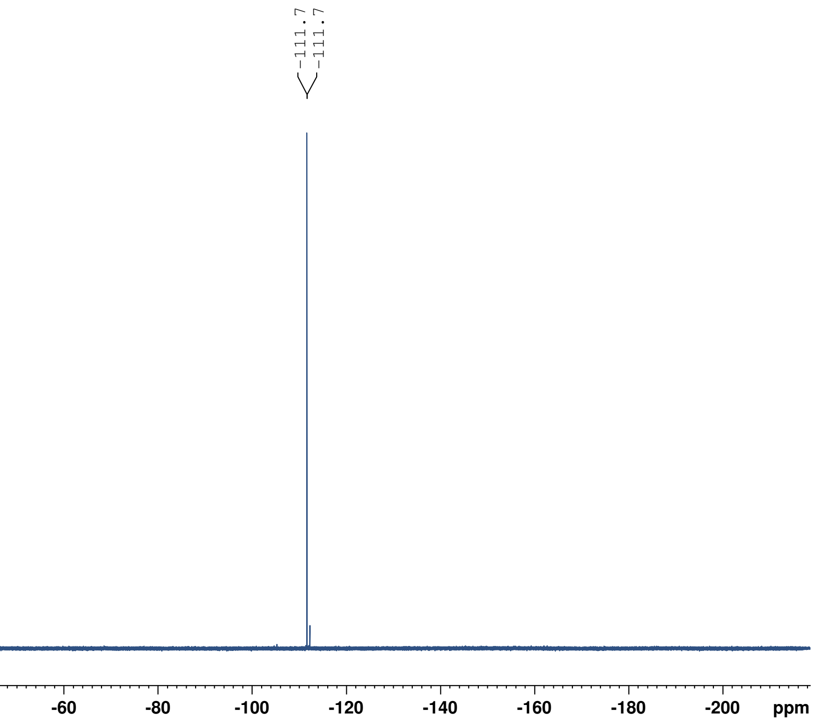

Compound 4a

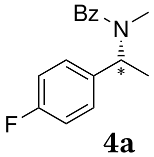

4a

^{1}H NMR spectrum

(CD_3CN, 600.15 MHz, 333 K)

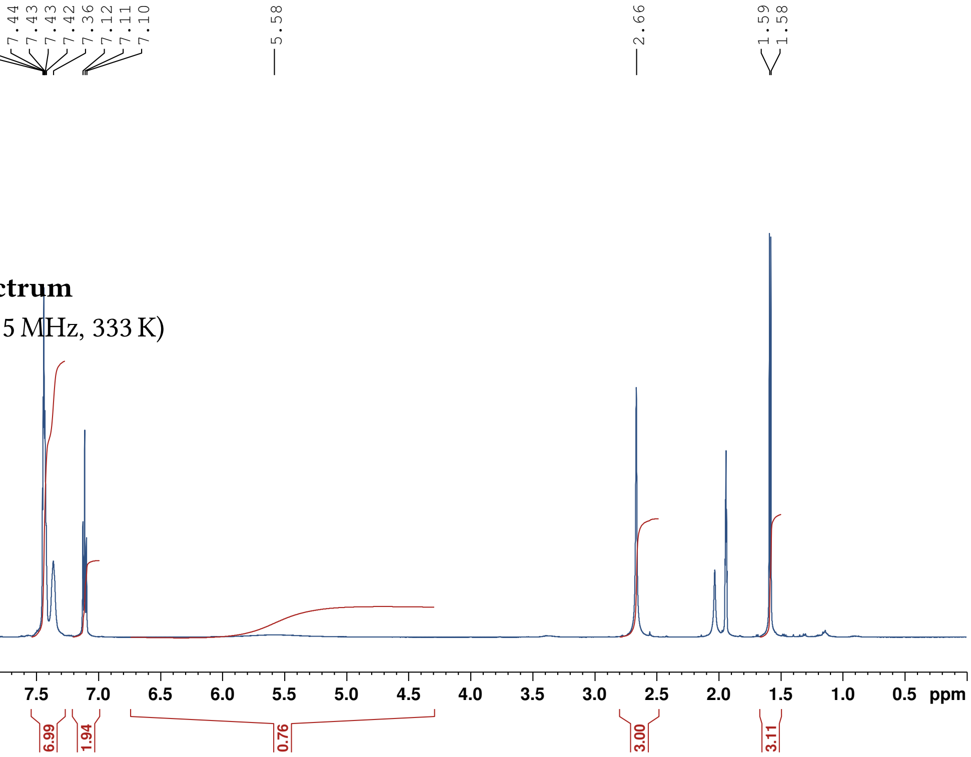

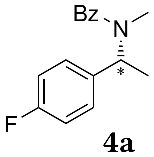

4a

^{13}C NMR spectrum

(CD_3CN, 150.90 MHz, 333 K)

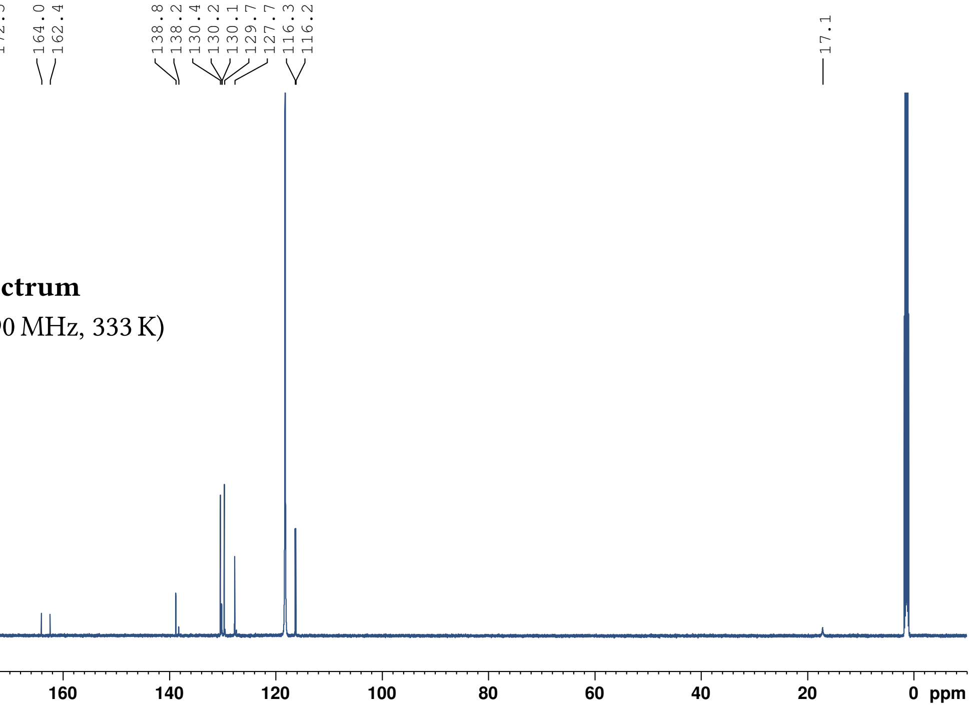

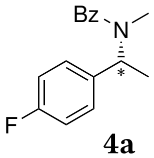

4a

^{19}F NMR spectrum

(CD_3CN, 376.27 MHz, 295 K)

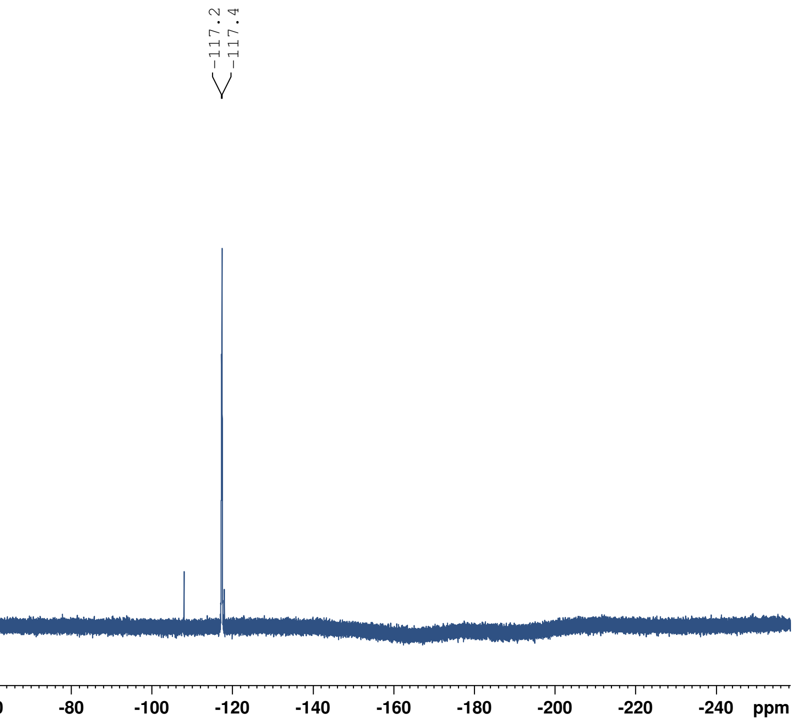

Compound 4b

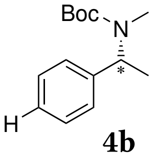

4b

^{1}H NMR spectrum
(CD_3CN, 600.15 MHz, 333 K)

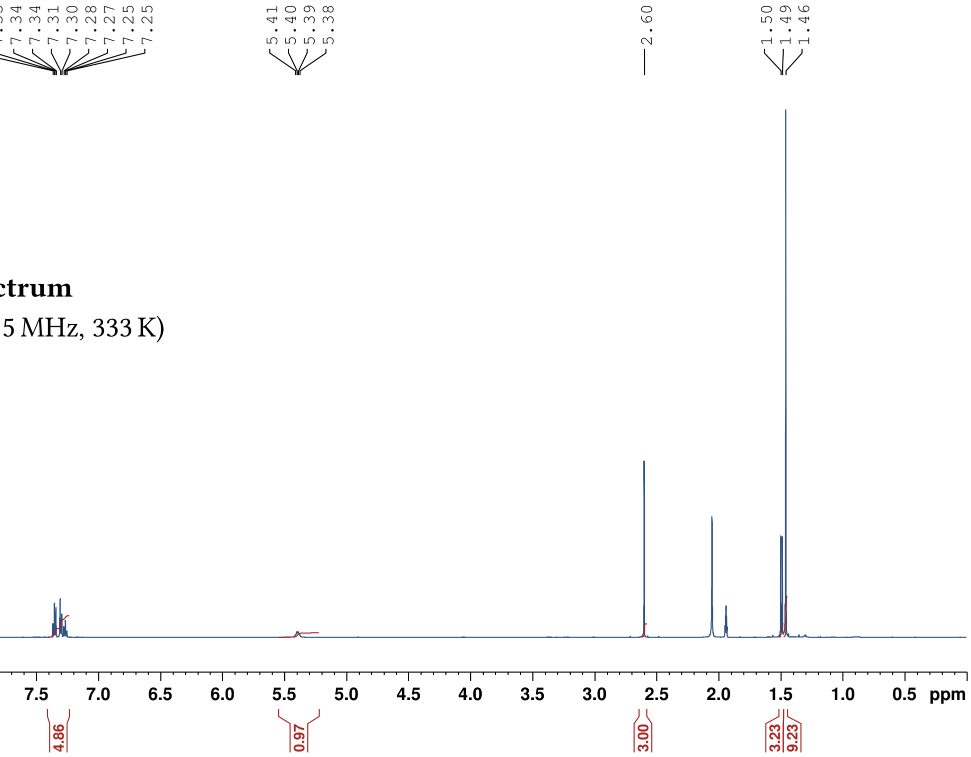

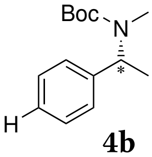

4b

^{13}C NMR spectrum
(CD_3CN, 150.90 MHz, 333 K)

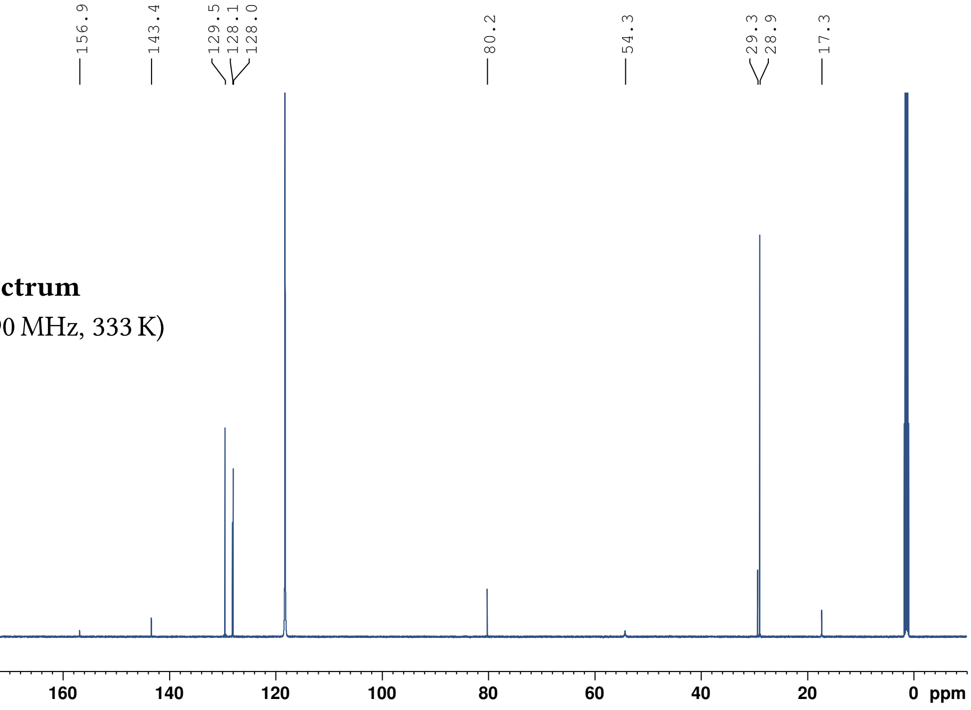

Compound 4c

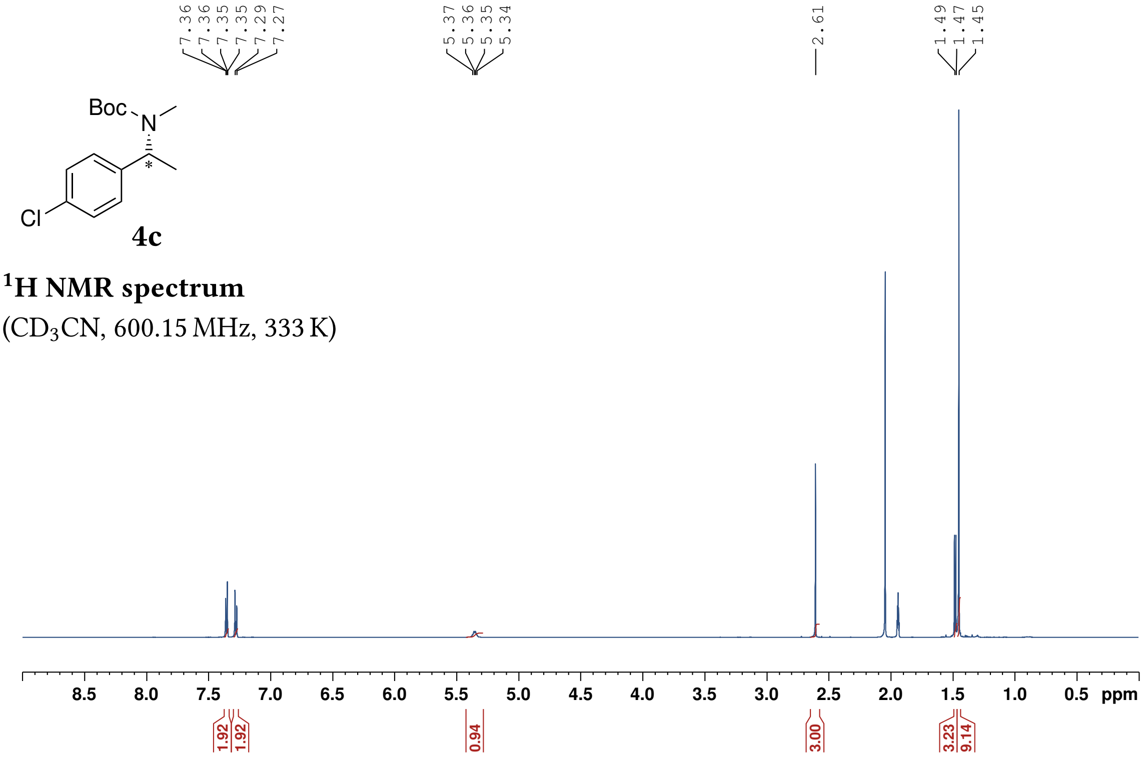

4c

^{1}H NMR spectrum

(CD_3CN, 600.15 MHz, 333 K)

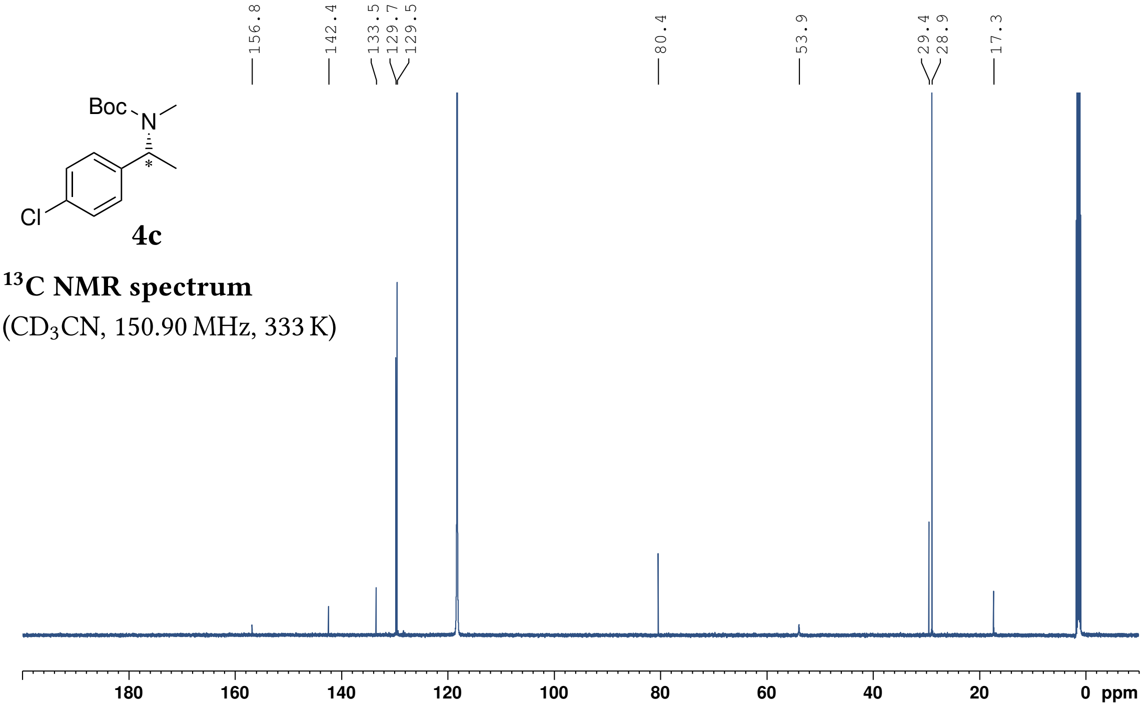

4c

^{13}C NMR spectrum

(CD_3CN, 150.90 MHz, 333 K)

Compound 4d

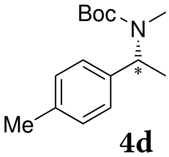

4d

^{1}H NMR spectrum

(CD$_3$CN, 600.15 MHz, 333 K)

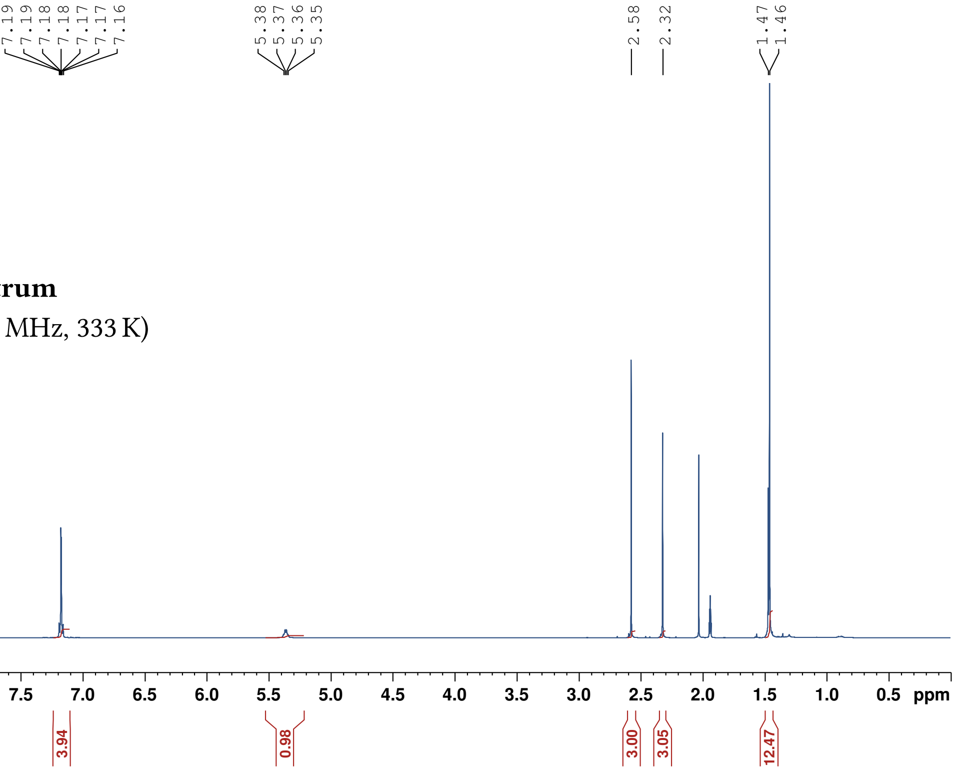

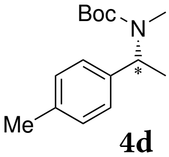

4d

^{13}C NMR spectrum

(CD$_3$CN, 150.90 MHz, 333 K)

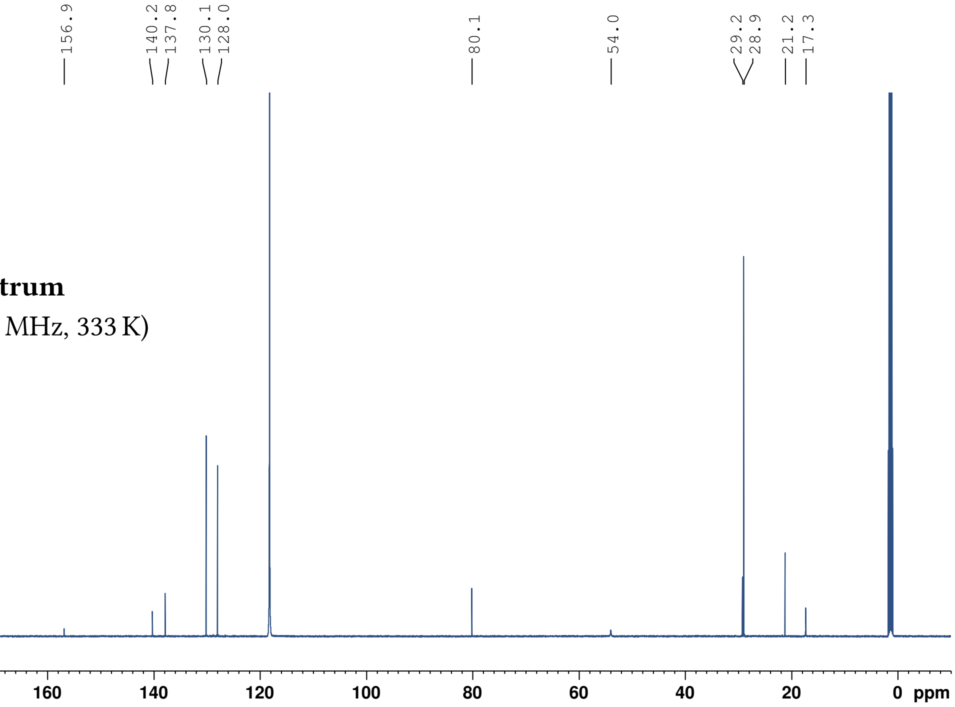

Compound 4e

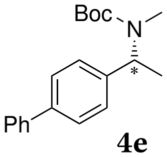

4e

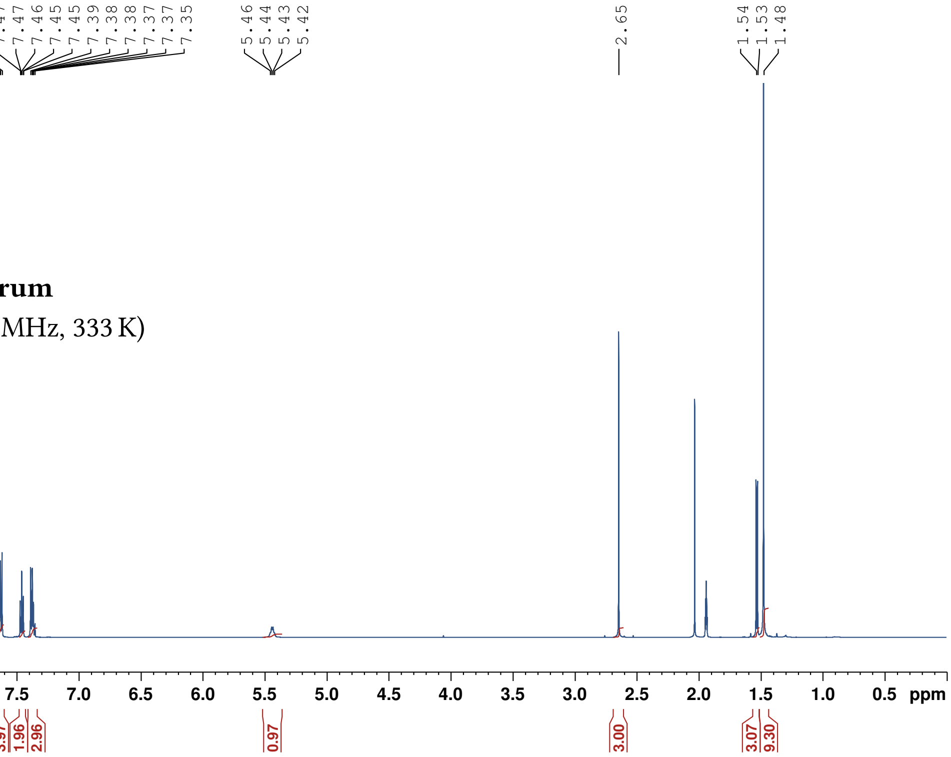

^{1}H NMR spectrum
(CD_3CN, 600.15 MHz, 333 K)

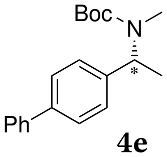

4e

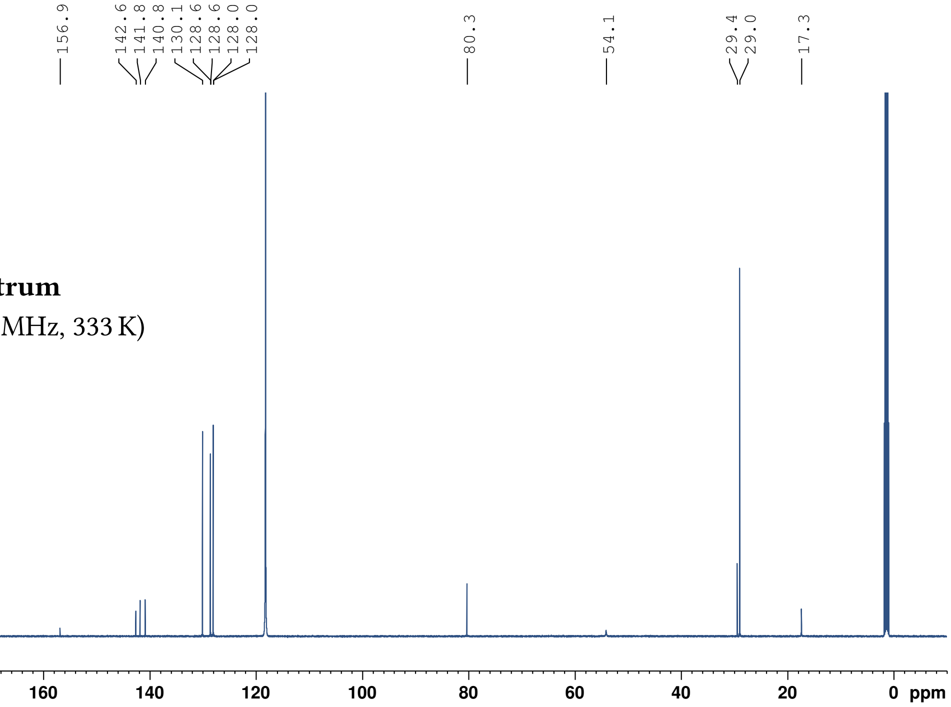

^{13}C NMR spectrum
(CD_3CN, 150.90 MHz, 333 K)

Compound 4f

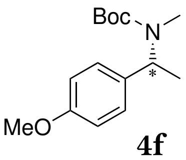

4f

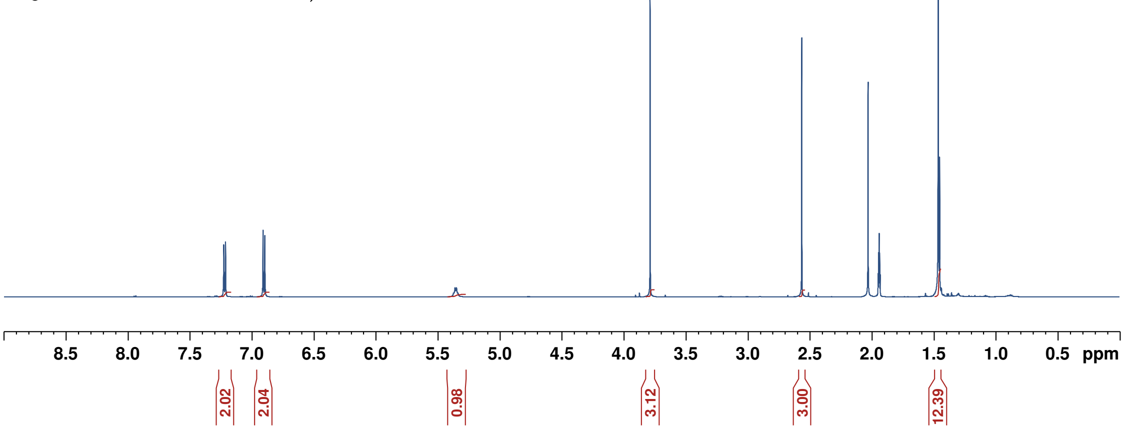

^{1}H NMR spectrum

(CD_3CN, 600.15 MHz, 333 K)

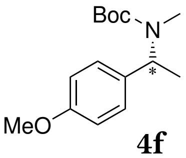

4f

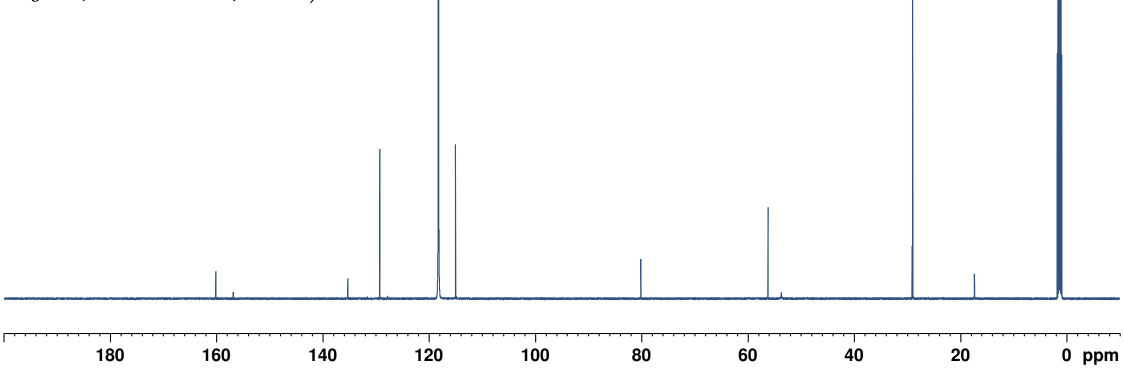

^{13}C NMR spectrum

(CD_3CN, 150.90 MHz, 333 K)

Compound 4g

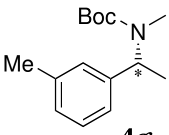

4g

^{1}H NMR spectrum
(CD_3CN, 600.15 MHz, 333 K)

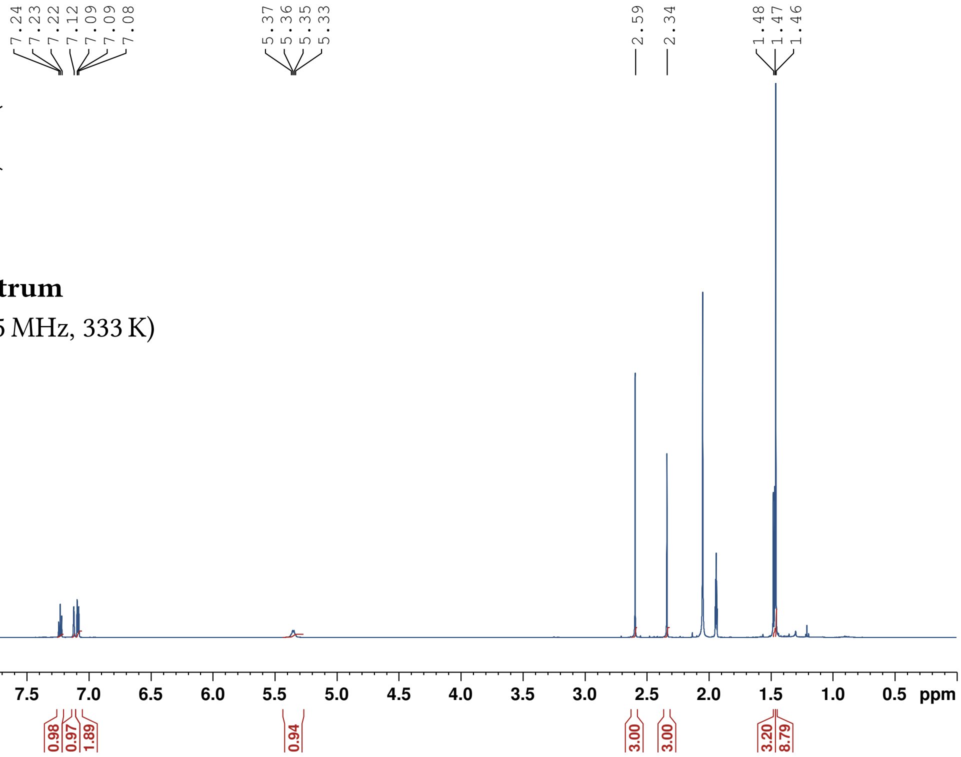

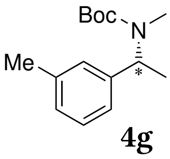

4g

^{13}C NMR spectrum
(CD_3CN, 150.90 MHz, 333 K)

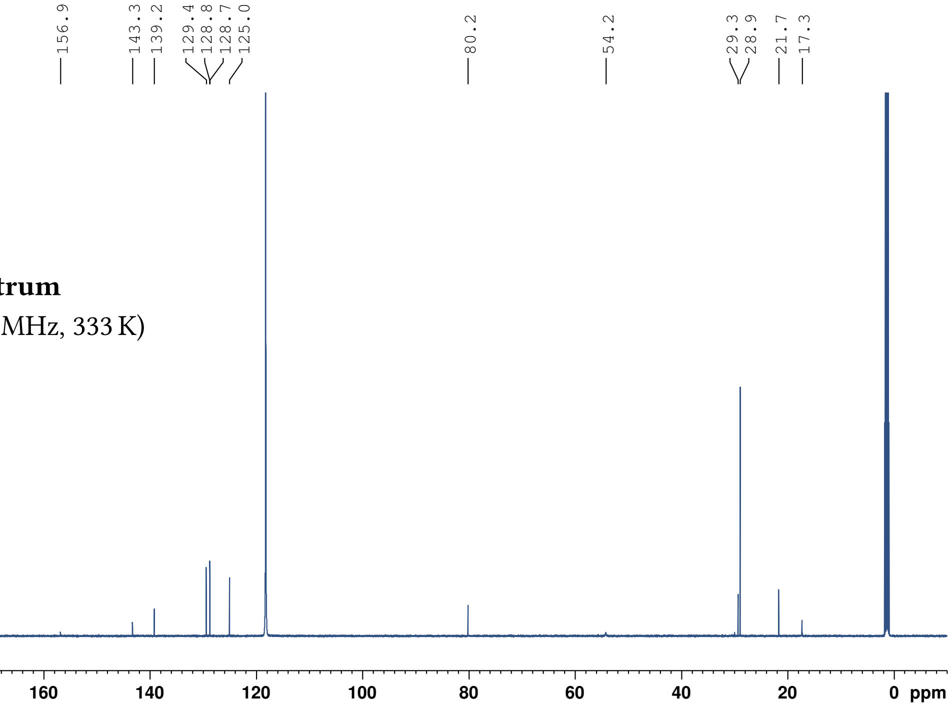

Compound 4h

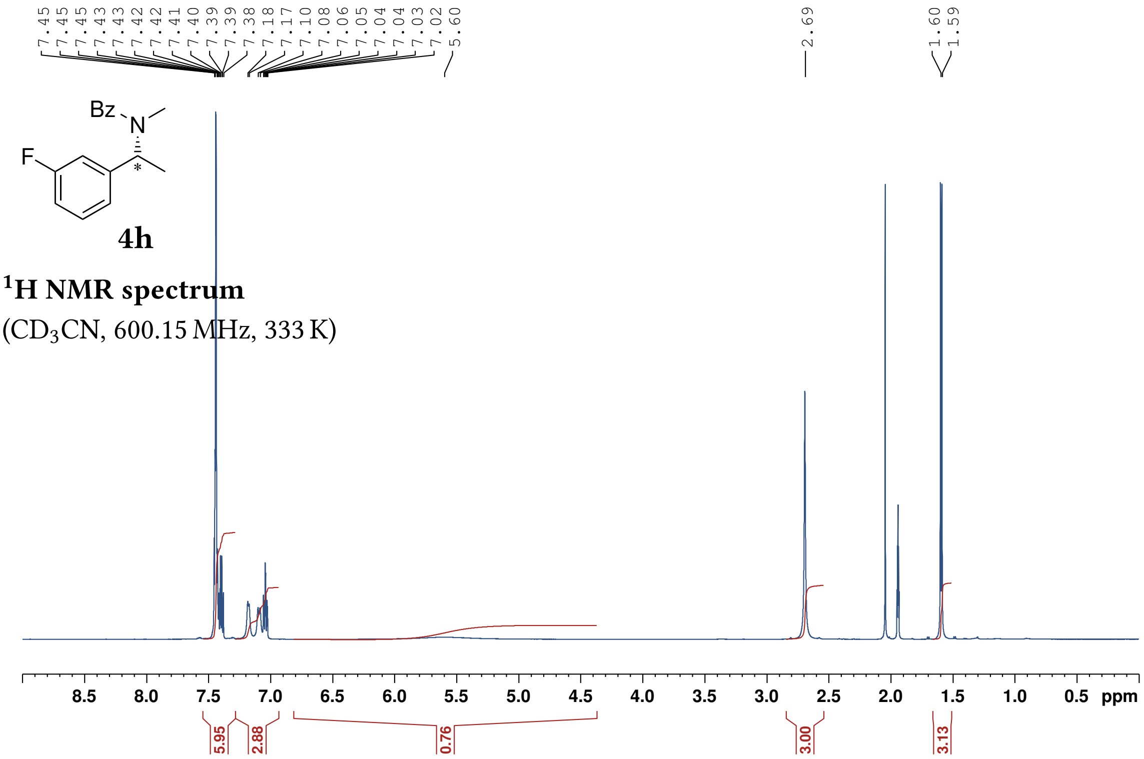

4h

^{1}H NMR spectrum
(CD_3CN, 600.15 MHz, 333 K)

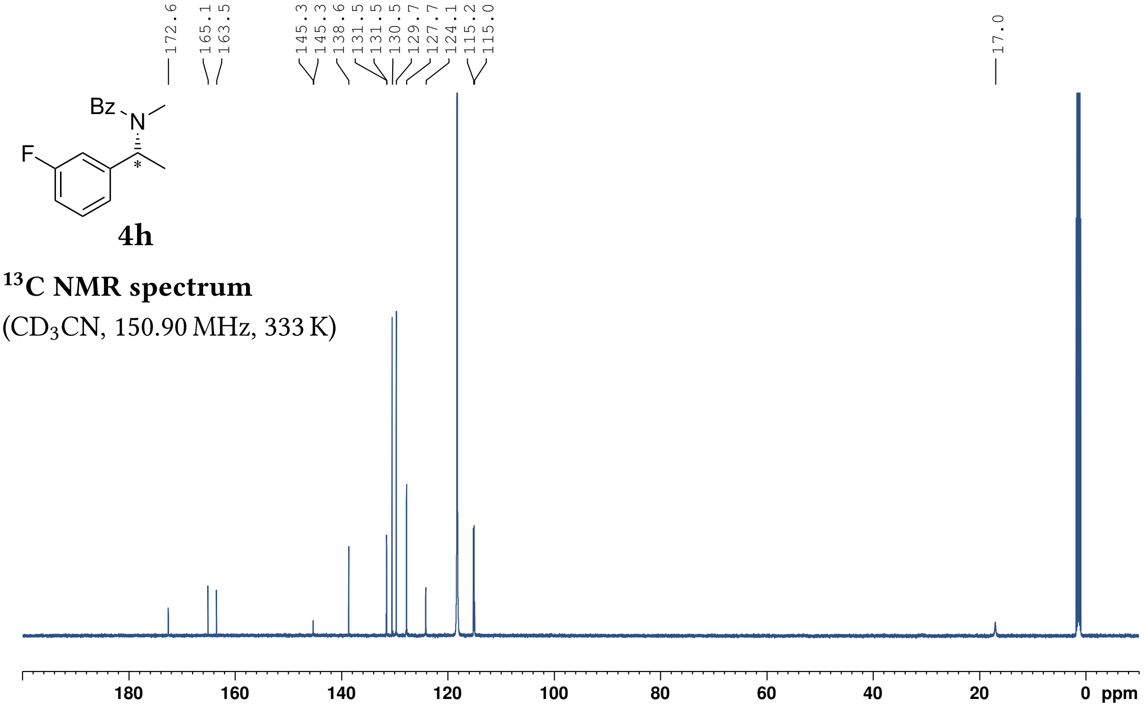

4h

^{13}C NMR spectrum
(CD_3CN, 150.90 MHz, 333 K)

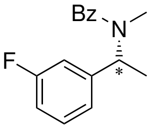

4h

^{19}F NMR spectrum
(CD_3CN, 376.27 MHz, 295 K)

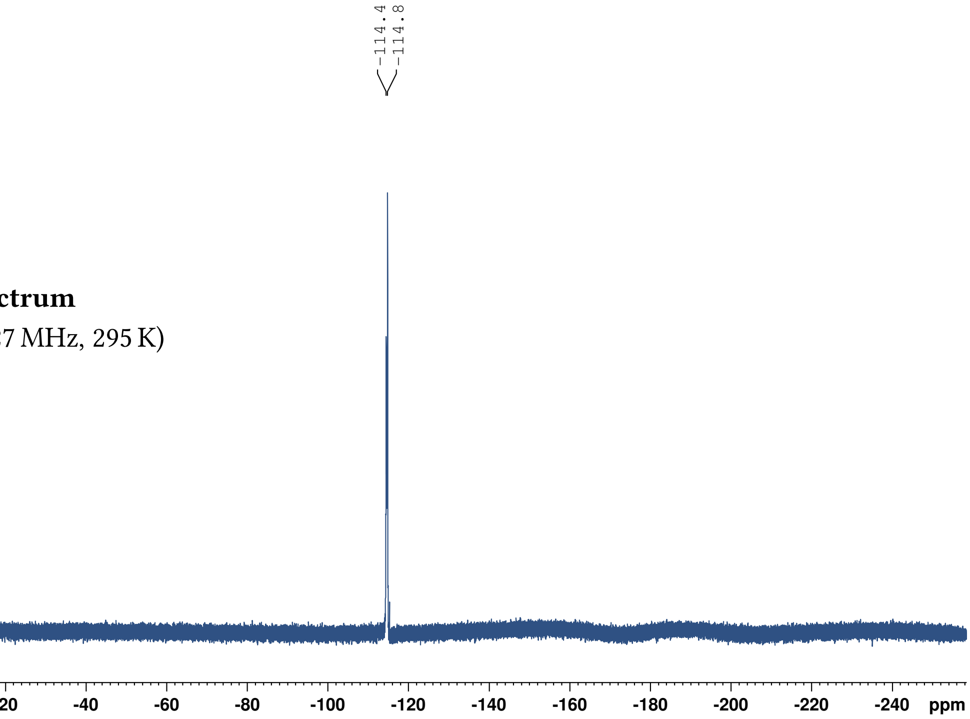

Compound 4i

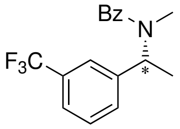

4i

1H NMR spectrum

(CD_3CN, 600.15 MHz, 333 K)

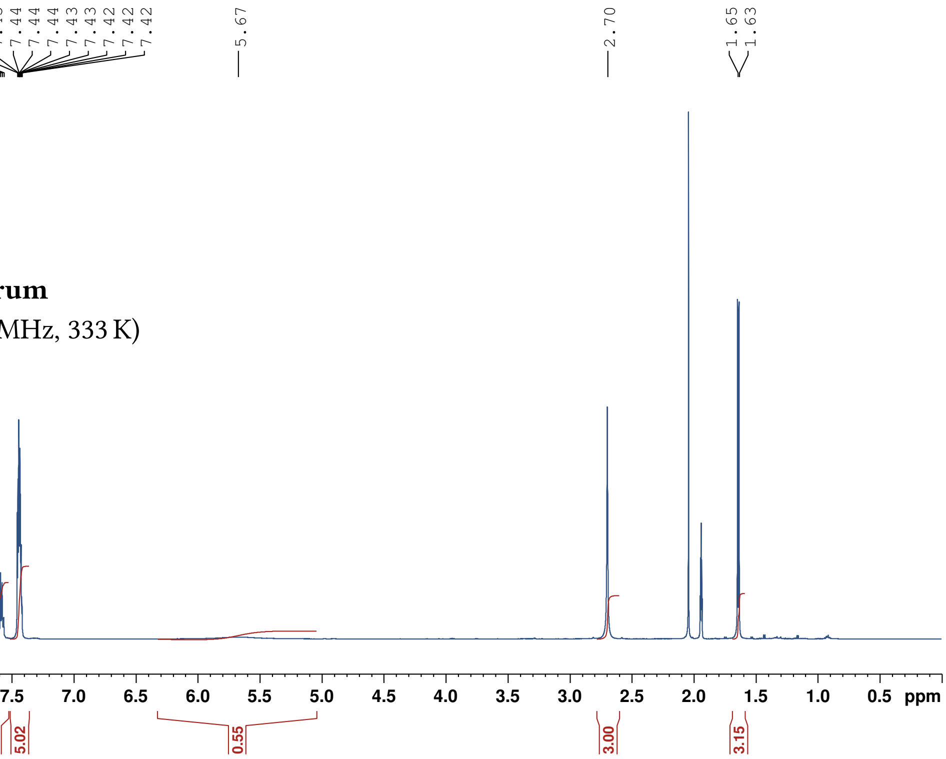

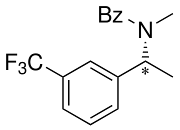

4i

13C NMR spectrum

(CD_3CN, 150.90 MHz, 333 K)

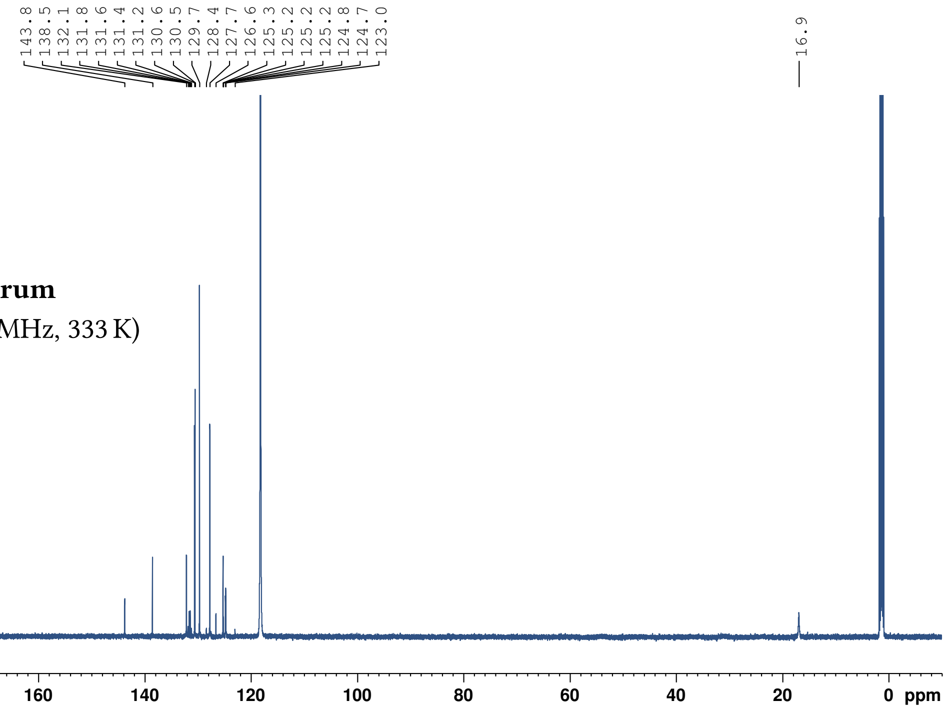

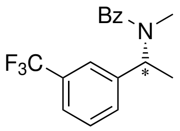

4i

^{19}F NMR spectrum

(CD_3CN, 376.27 MHz, 295 K)

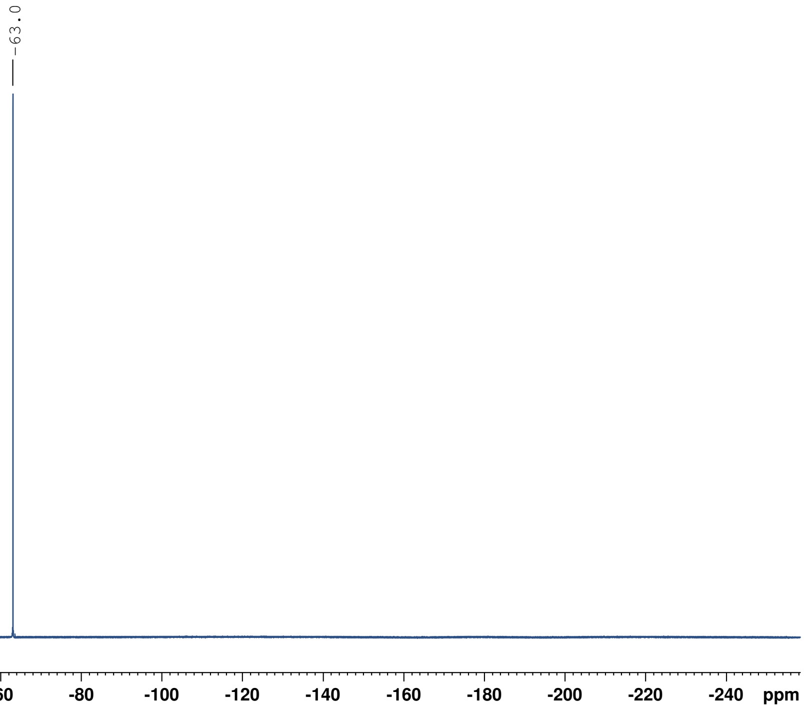

Compound 4j

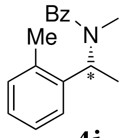

4j

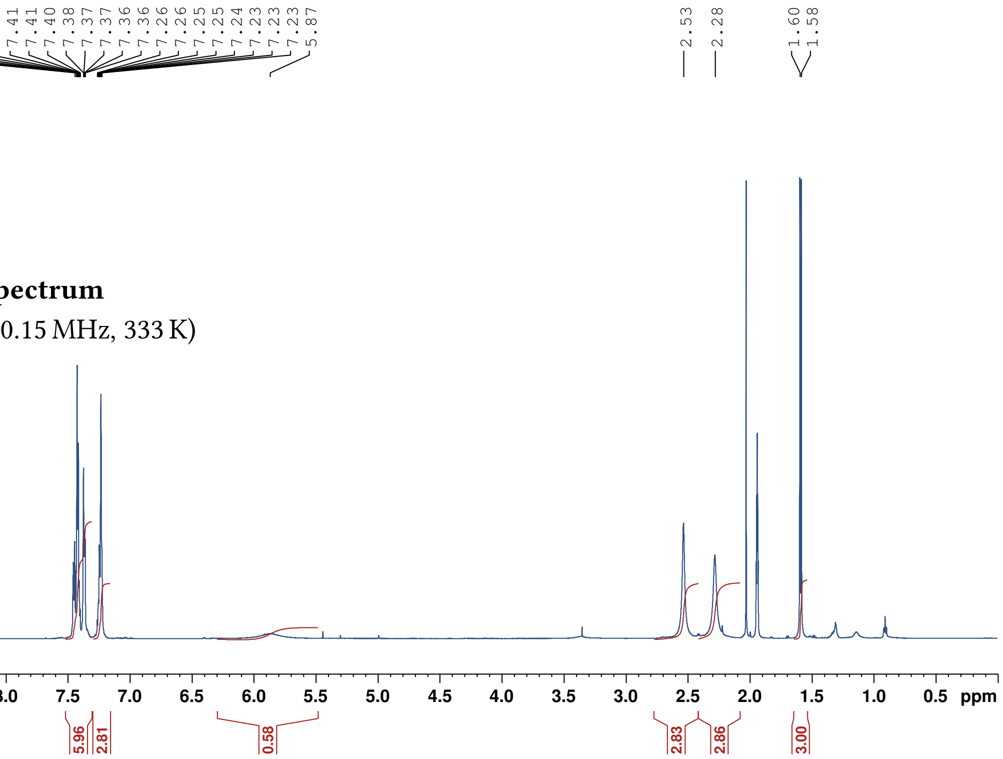

^{1}H NMR spectrum
(CD$_3$CN, 600.15 MHz, 333 K)

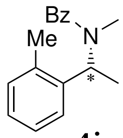

4j

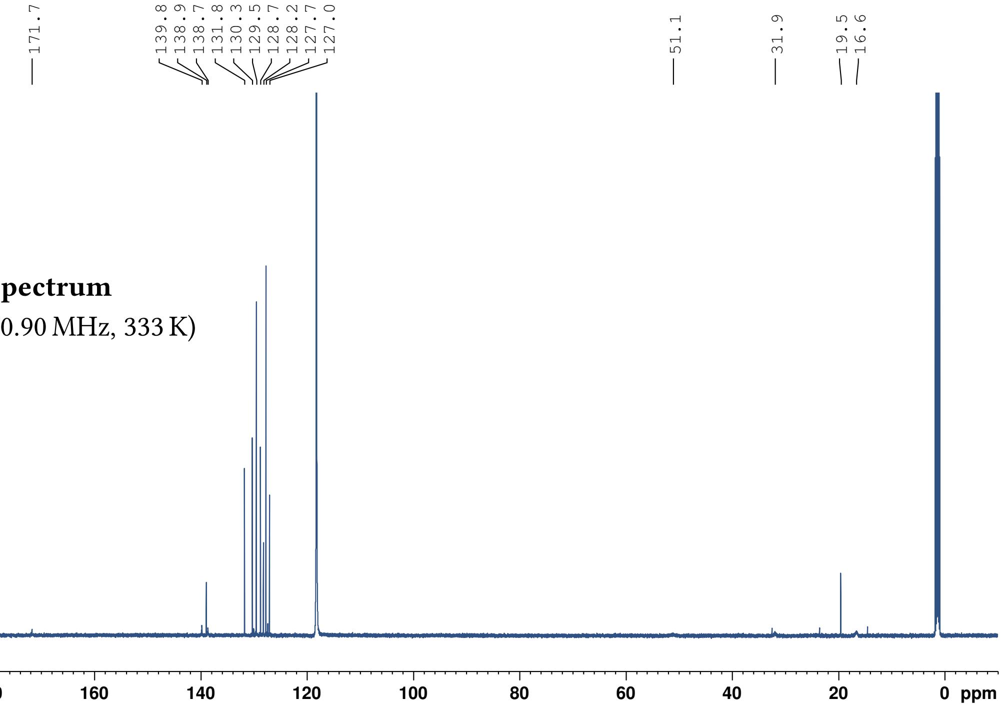

^{13}C NMR spectrum
(CD$_3$CN, 150.90 MHz, 333 K)

Compound 4k

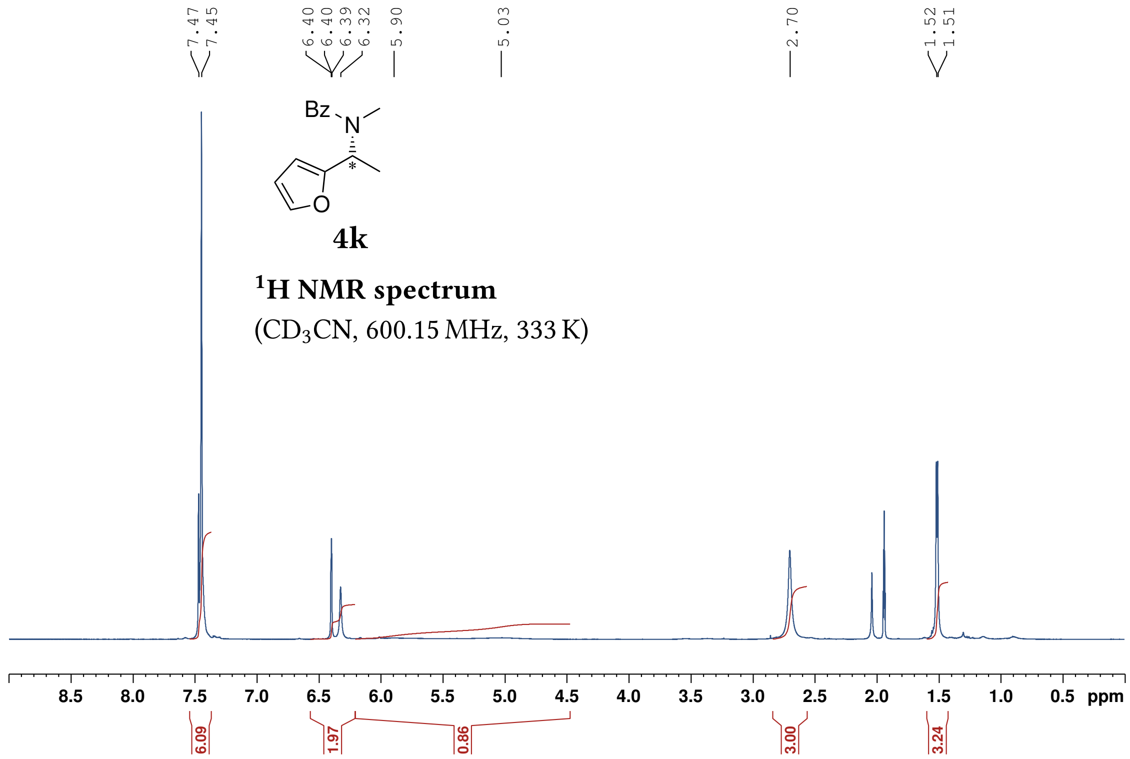

4k

^{1}H NMR spectrum

(CD_3CN, 600.15 MHz, 333 K)

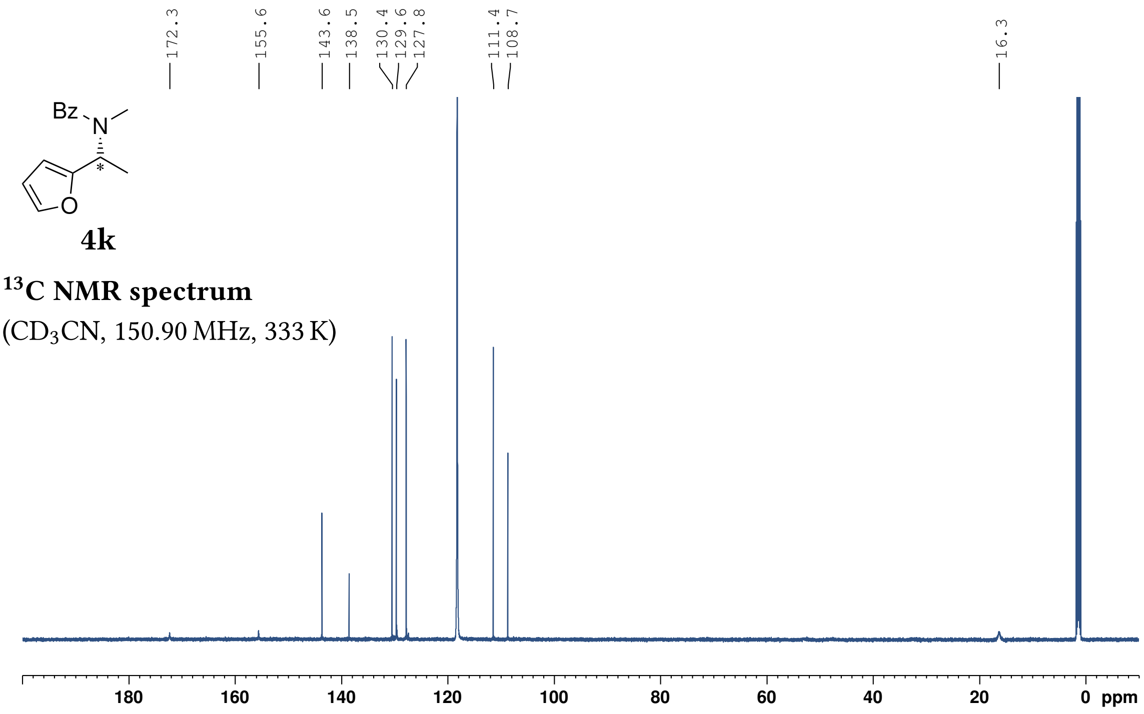

4k

^{13}C NMR spectrum

(CD_3CN, 150.90 MHz, 333 K)

Compound 4l

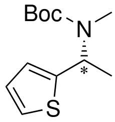

4l

^{1}H NMR spectrum

(CD_3CN, 600.15 MHz, 333 K)

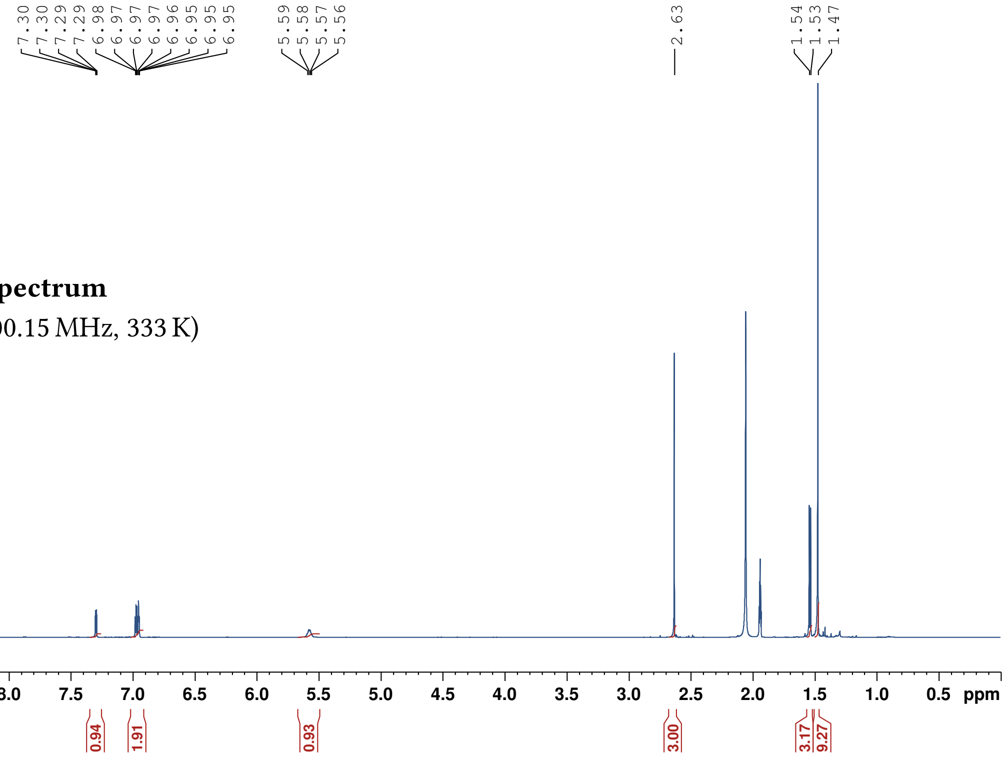

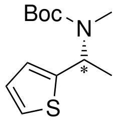

4l

^{13}C NMR spectrum

(CD_3CN, 150.90 MHz, 333 K)

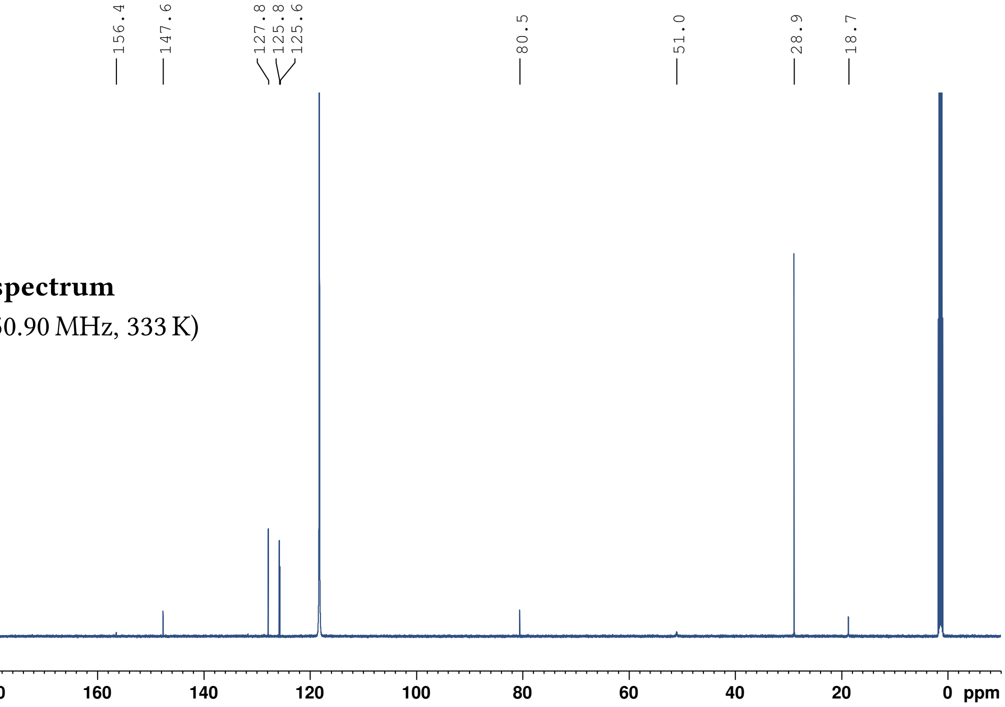

Compound 4m

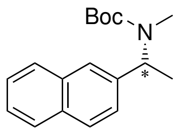

4m

^{1}H NMR spectrum

(CD_3CN, 600.15 MHz, 333 K)

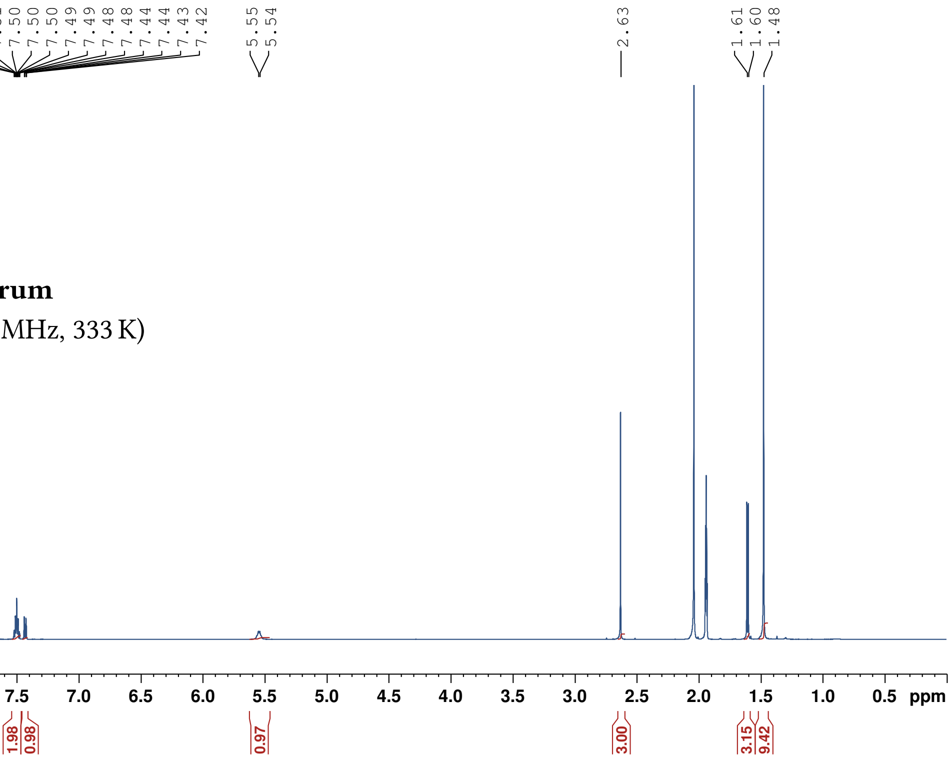

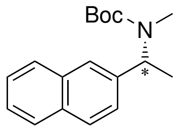

4m

^{13}C NMR spectrum

(CD_3CN, 150.90 MHz, 333 K)

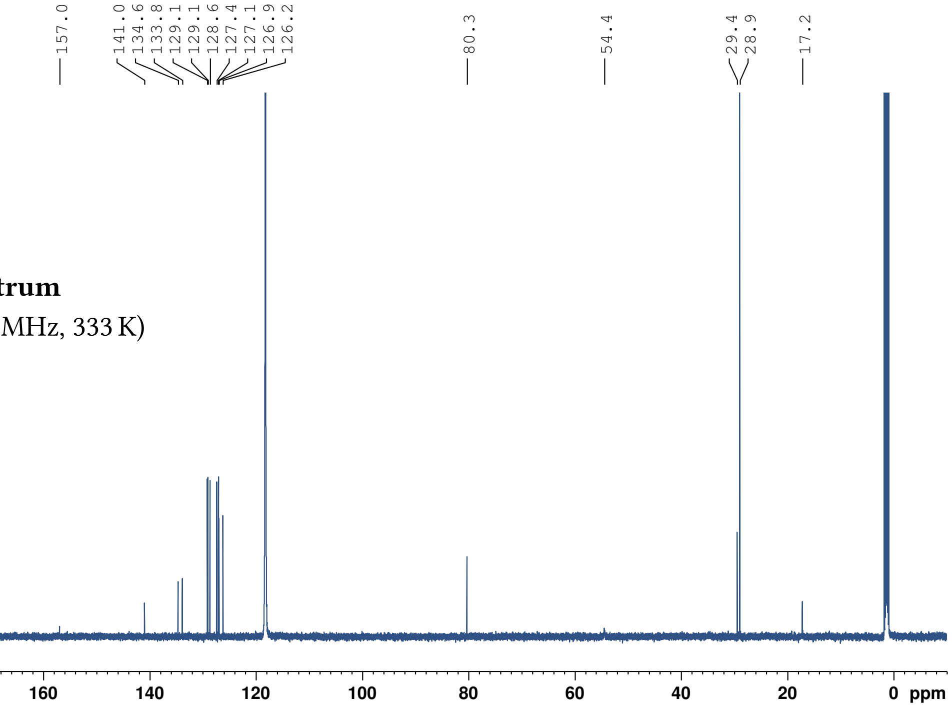

Compound 4n

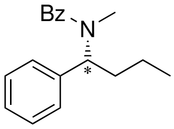

4n

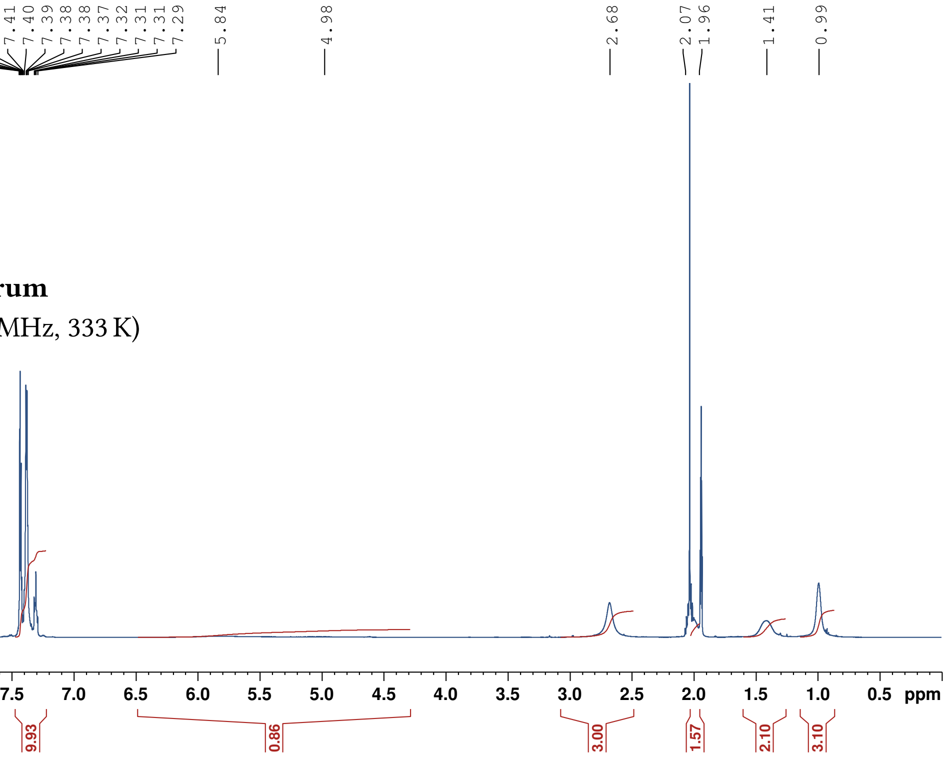

^{1}H NMR spectrum

(CD_3CN, 600.15 MHz, 333 K)

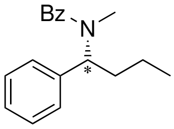

4n

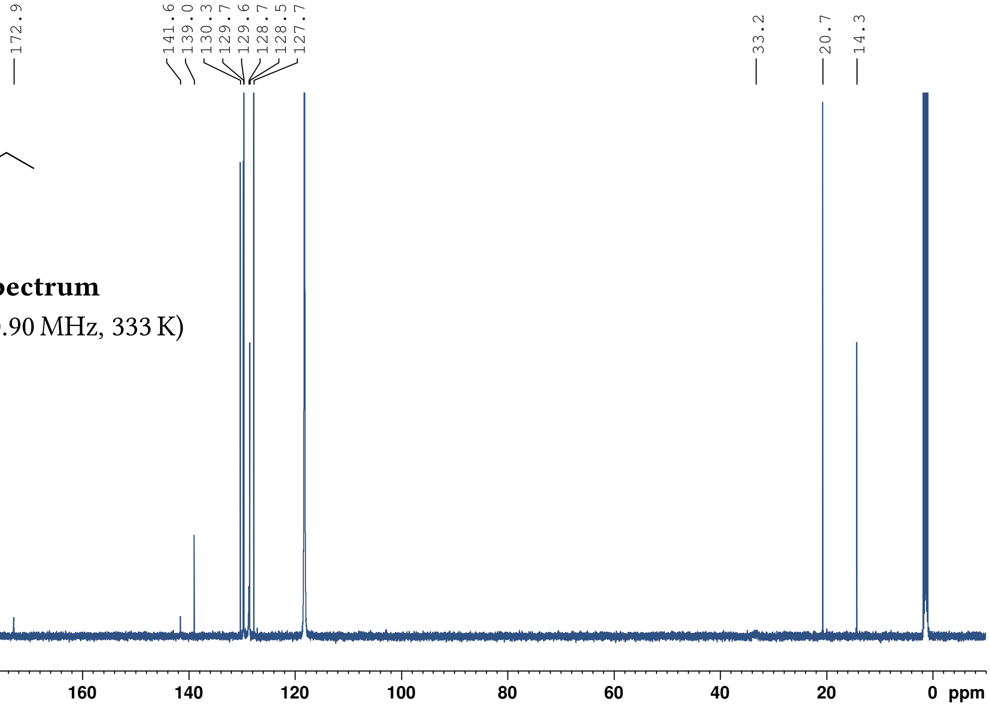

^{13}C NMR spectrum

(CD_3CN, 150.90 MHz, 333 K)

Compound 4o

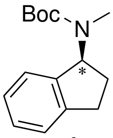

4o

^{1}H NMR spectrum

(CD_3CN, 600.15 MHz, 333 K)

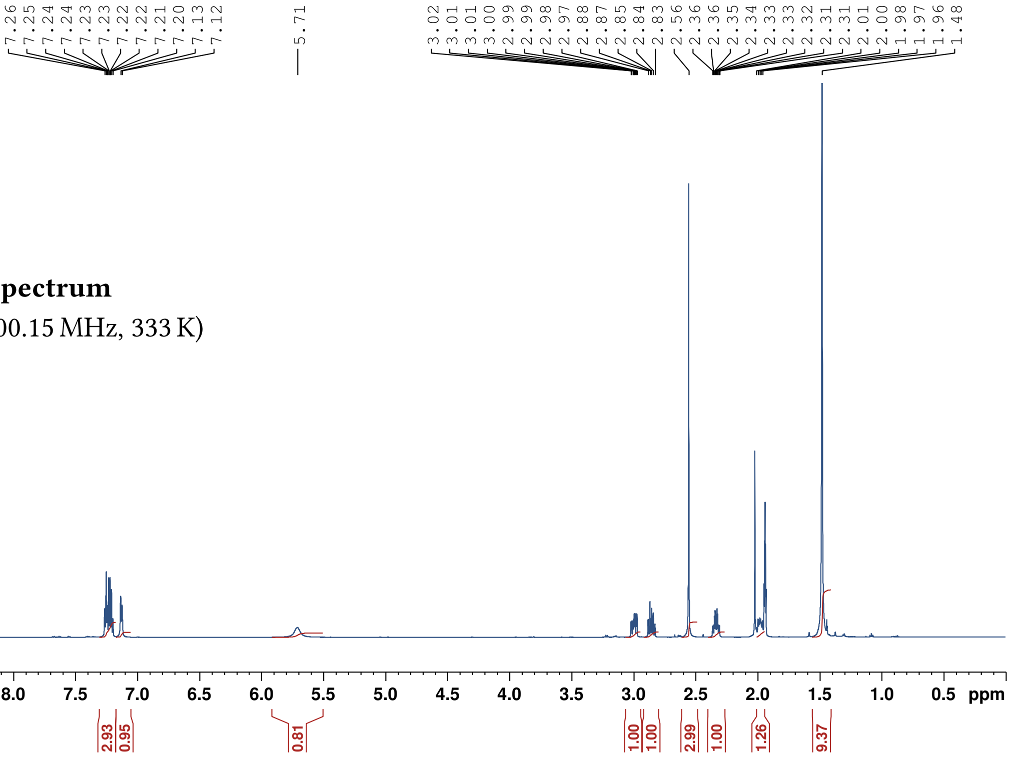

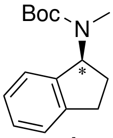

4o

^{13}C NMR spectrum

(CD_3CN, 150.90 MHz, 333 K)

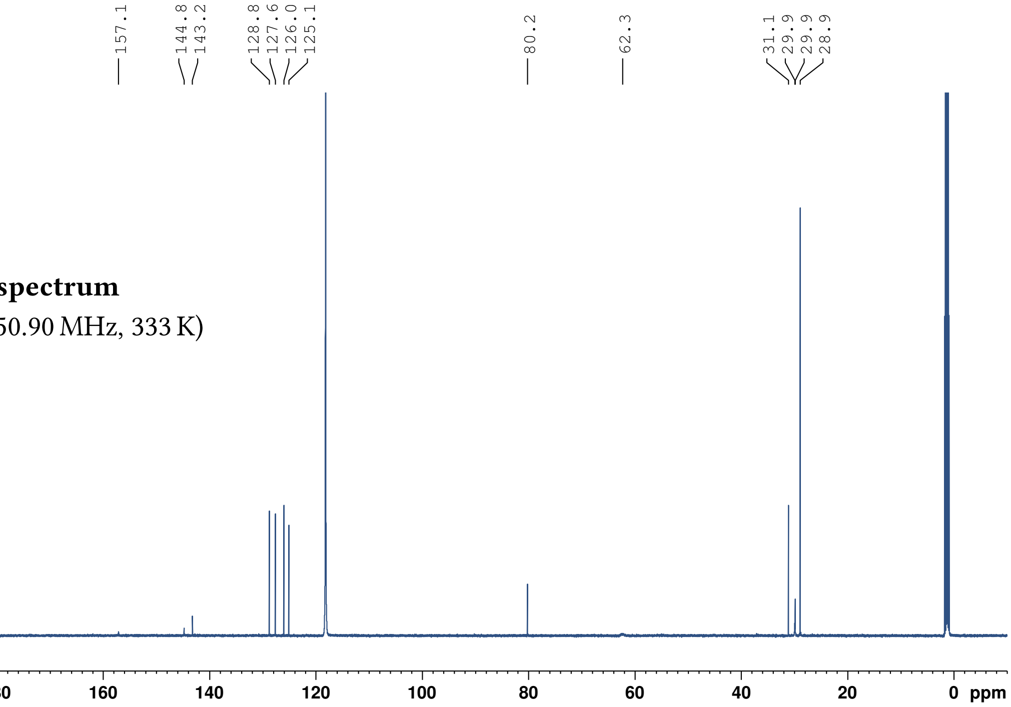

Compound 4p

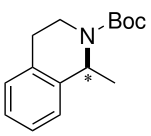

4p

^{1}H NMR spectrum
(CD_3CN, 600.15 MHz, 333 K)

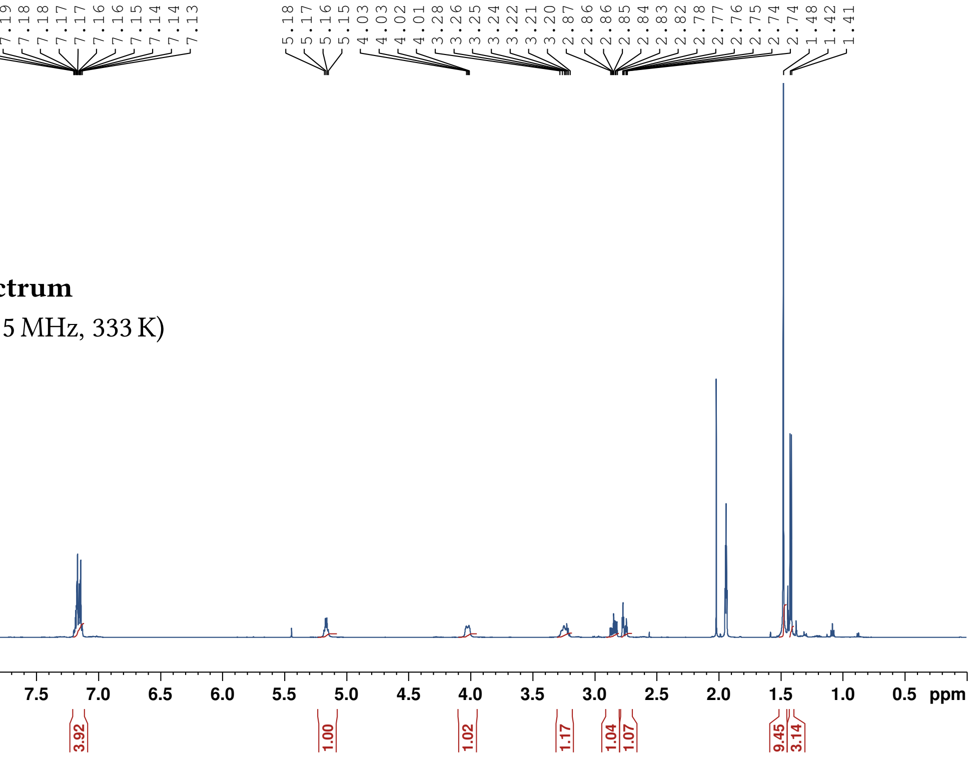

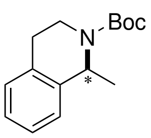

4p

^{13}C NMR spectrum
(CD_3CN, 150.90 MHz, 333 K)

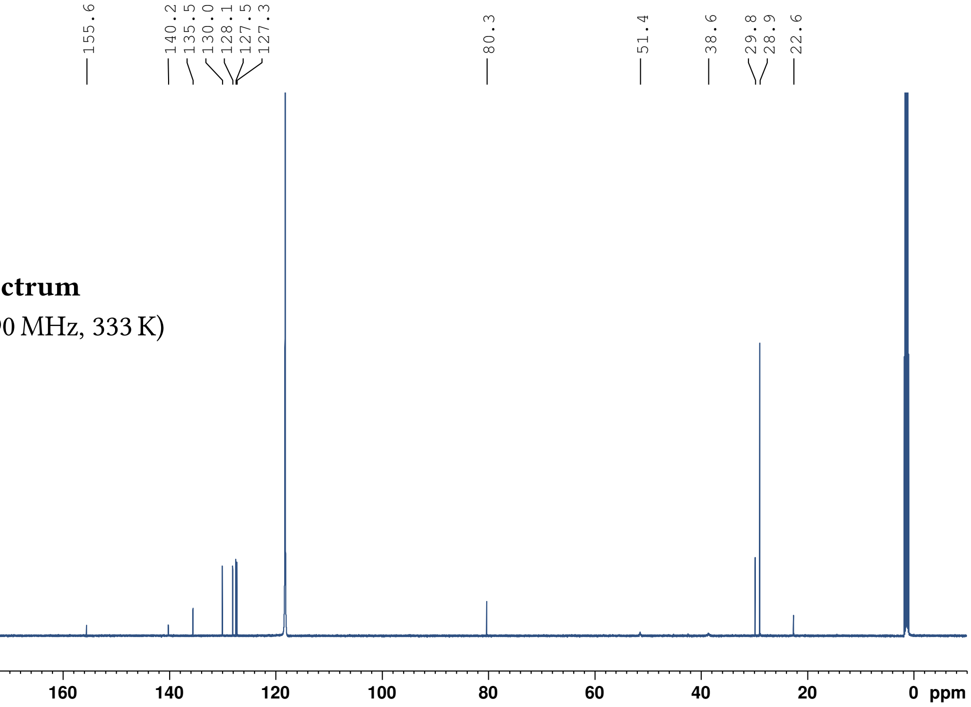

Compound 4q

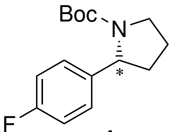

4q

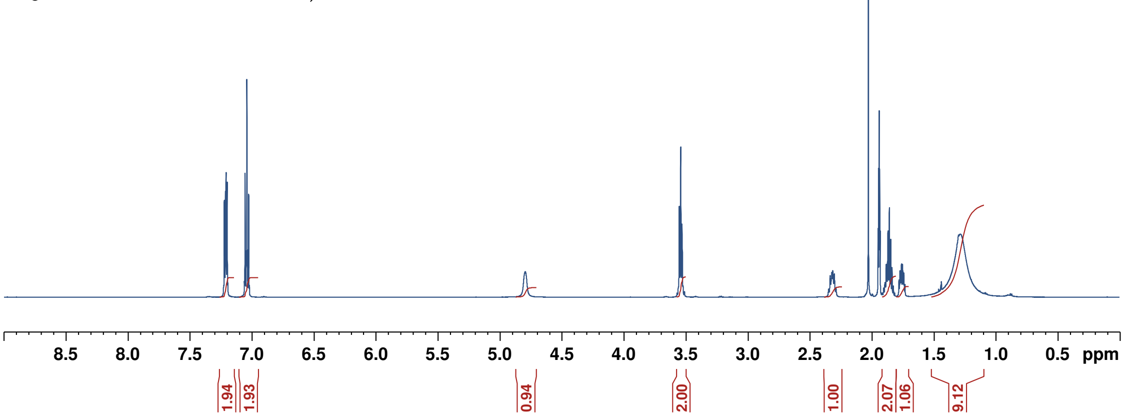

^{1}H NMR spectrum

(CD_3CN, 600.15 MHz, 333 K)

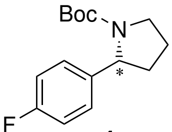

4q

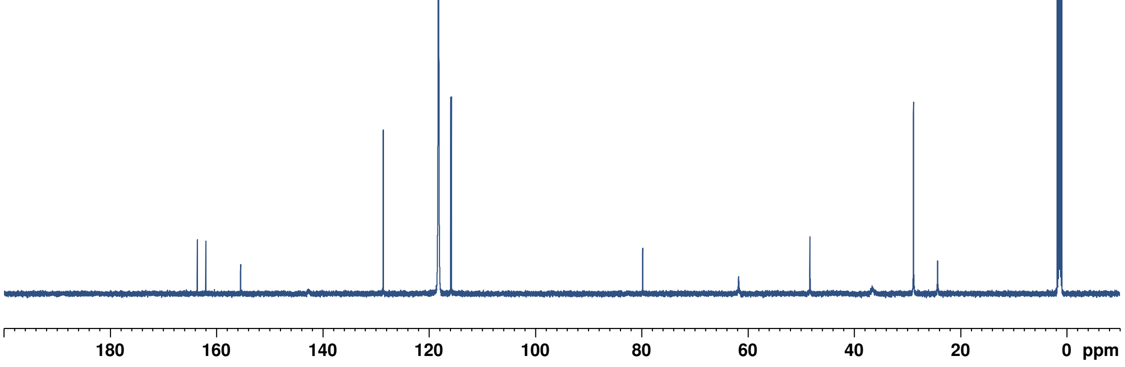

^{13}C NMR spectrum

(CD_3CN, 150.90 MHz, 333 K)

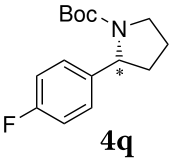

4q

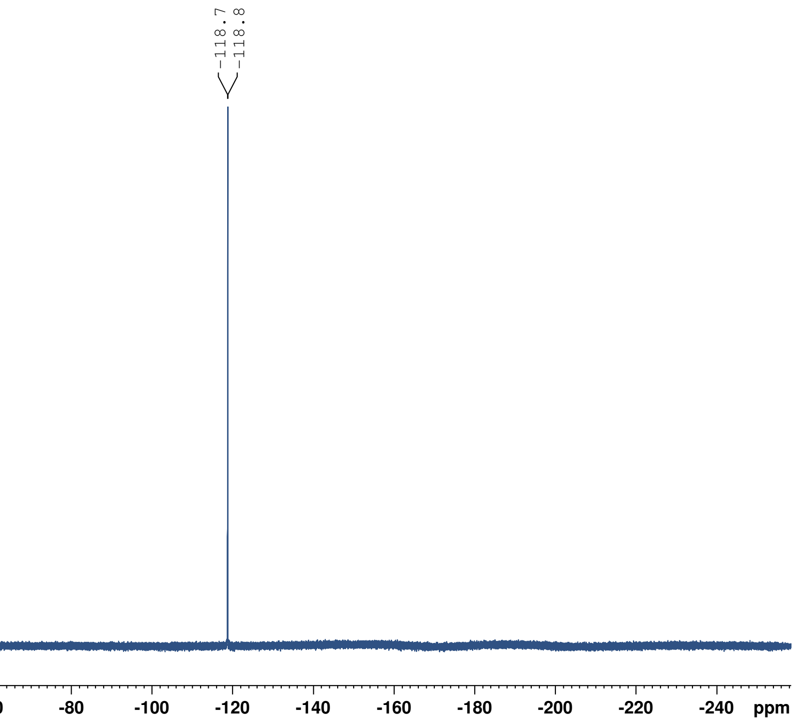

^{19}F NMR spectrum

(CD_3CN, 376.27 MHz, 295 K)

Compound 4r

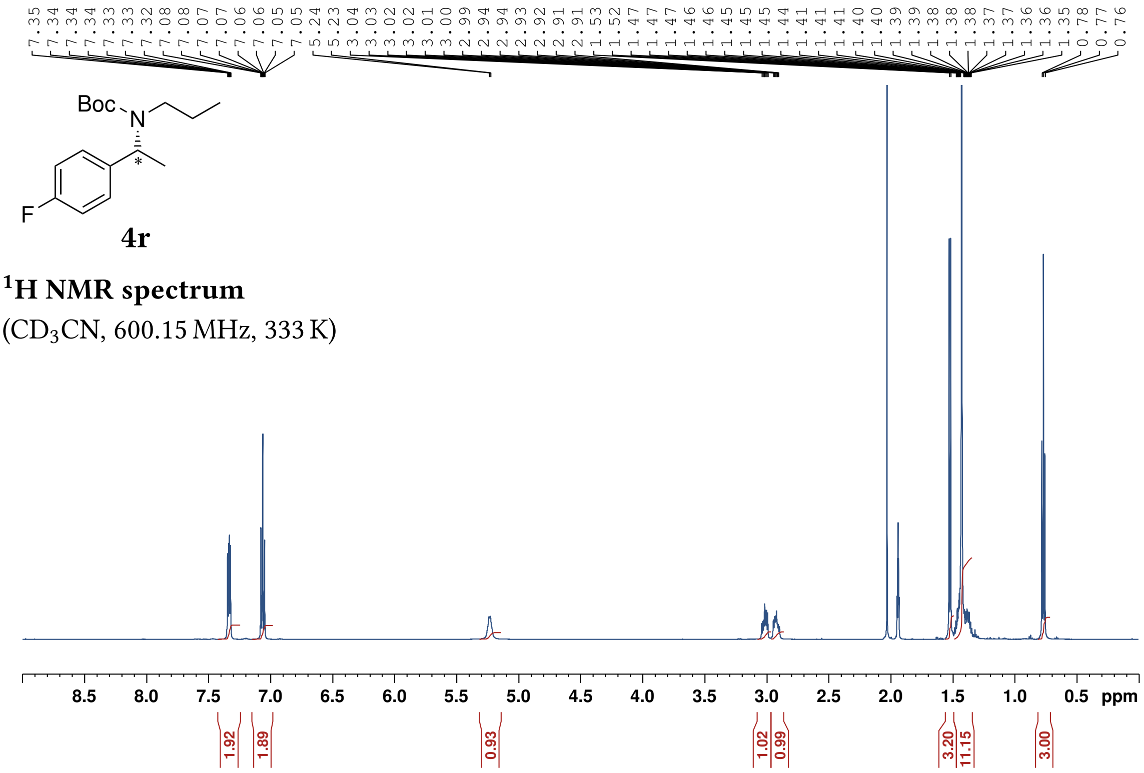

^{1}H NMR spectrum
(CD_3CN, 600.15 MHz, 333 K)

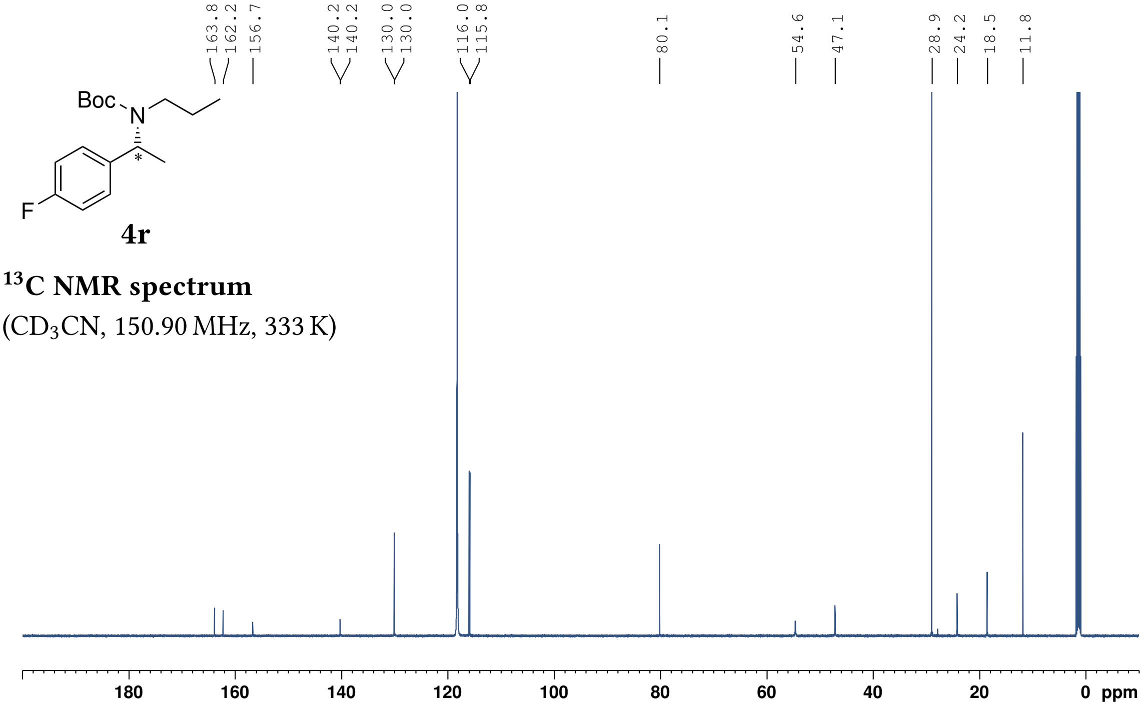

^{13}C NMR spectrum
(CD_3CN, 150.90 MHz, 333 K)

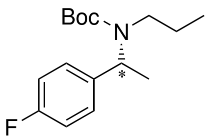

4r

^{19}F NMR spectrum

(CD_3CN, 376.27 MHz, 295 K)

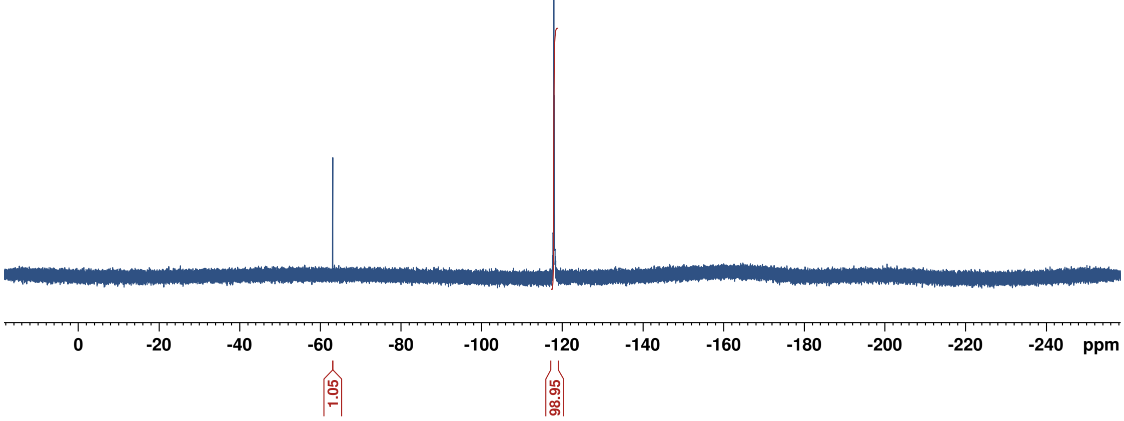

Compound 4s

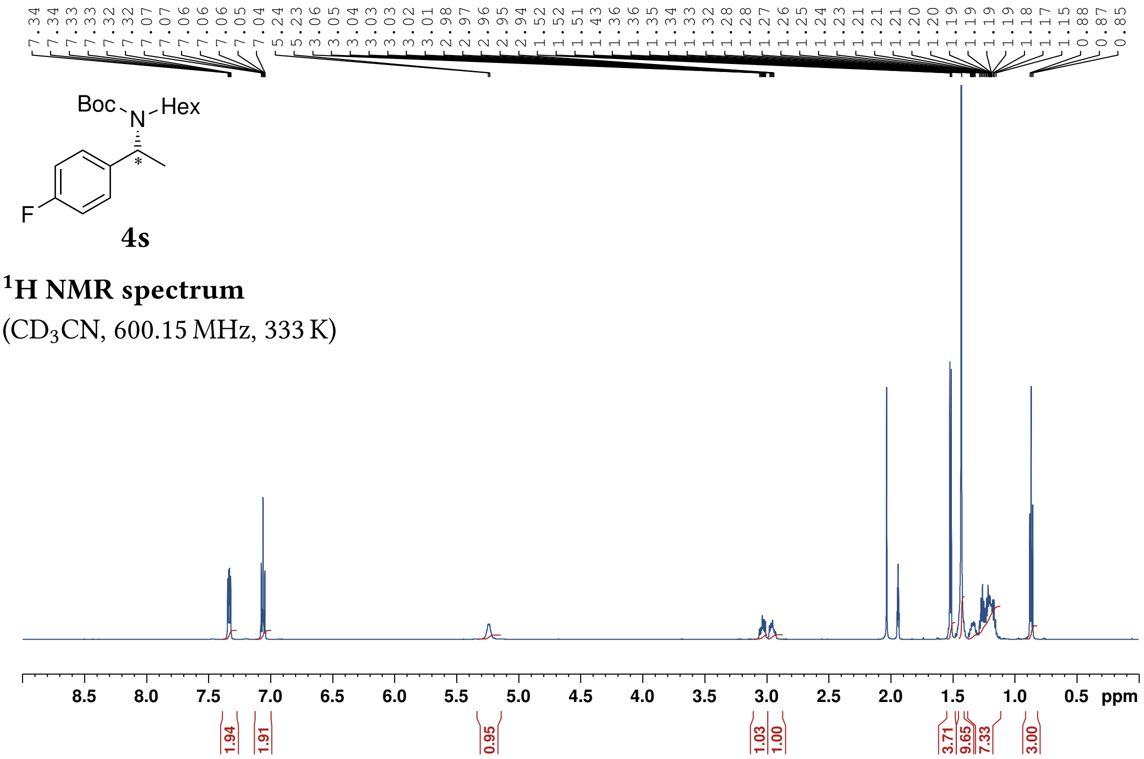

4s

^{1}H NMR spectrum

(CD_3CN, 600.15 MHz, 333 K)

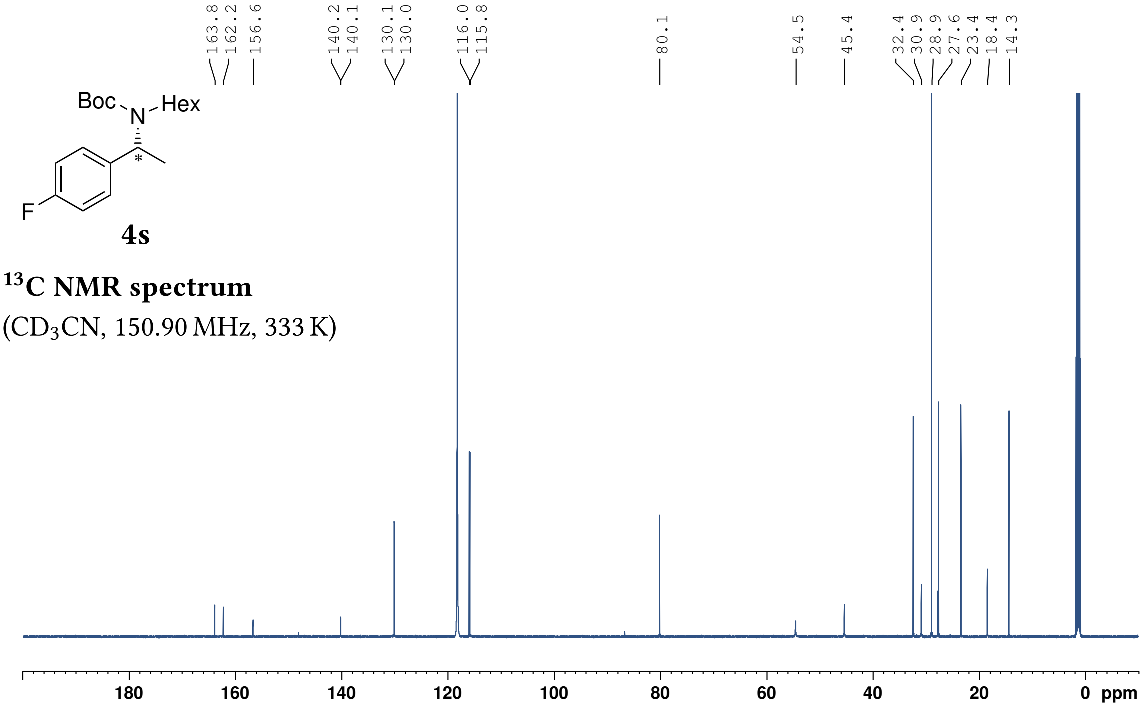

4s

^{13}C NMR spectrum

(CD_3CN, 150.90 MHz, 333 K)

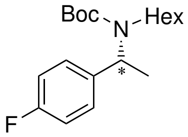

4s

^{19}F NMR spectrum
(CD_3CN, 376.27 MHz, 295 K)

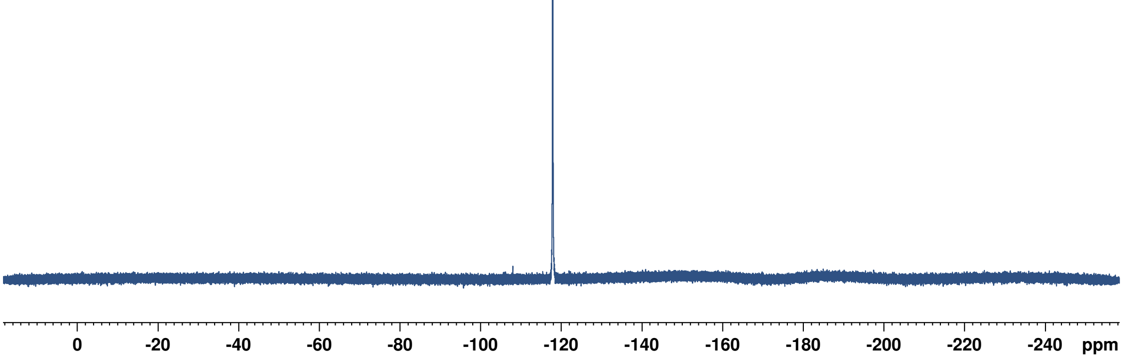

Compound 3t

3t

^{1}H NMR spectrum
($CDCl_3$, 600.15 MHz, 295 K)

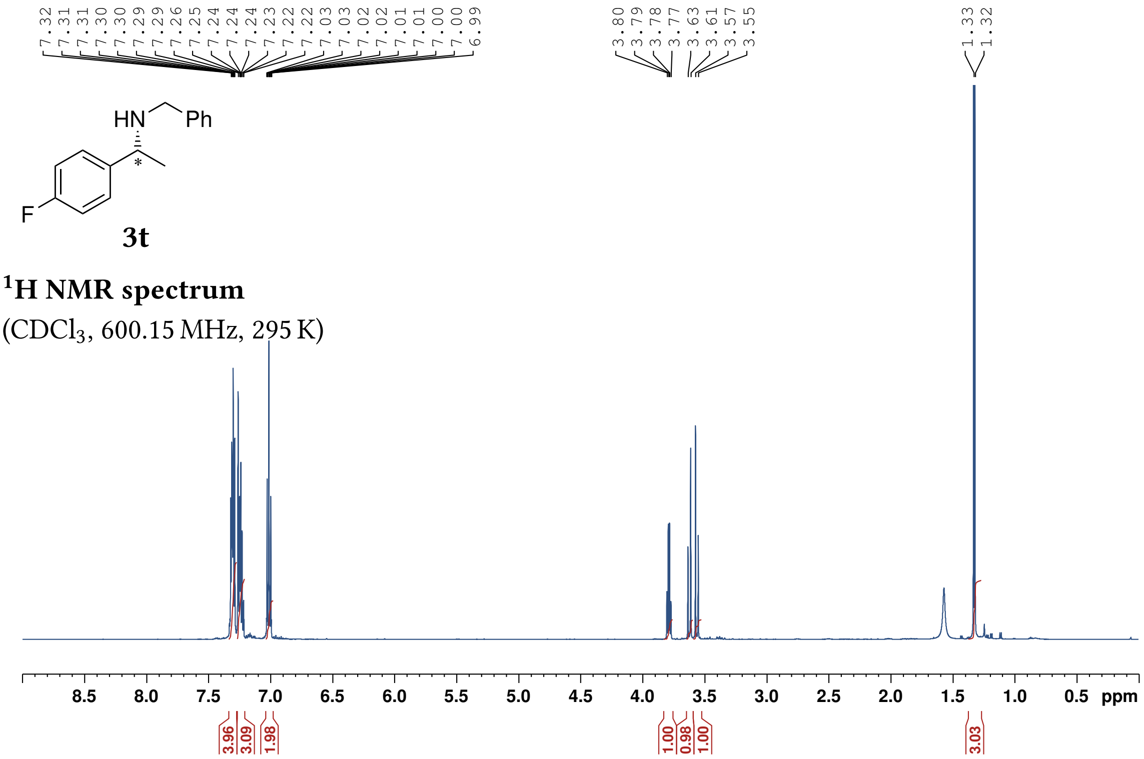

3t

^{13}C NMR spectrum
($CDCl_3$, 150.90 MHz, 295 K)

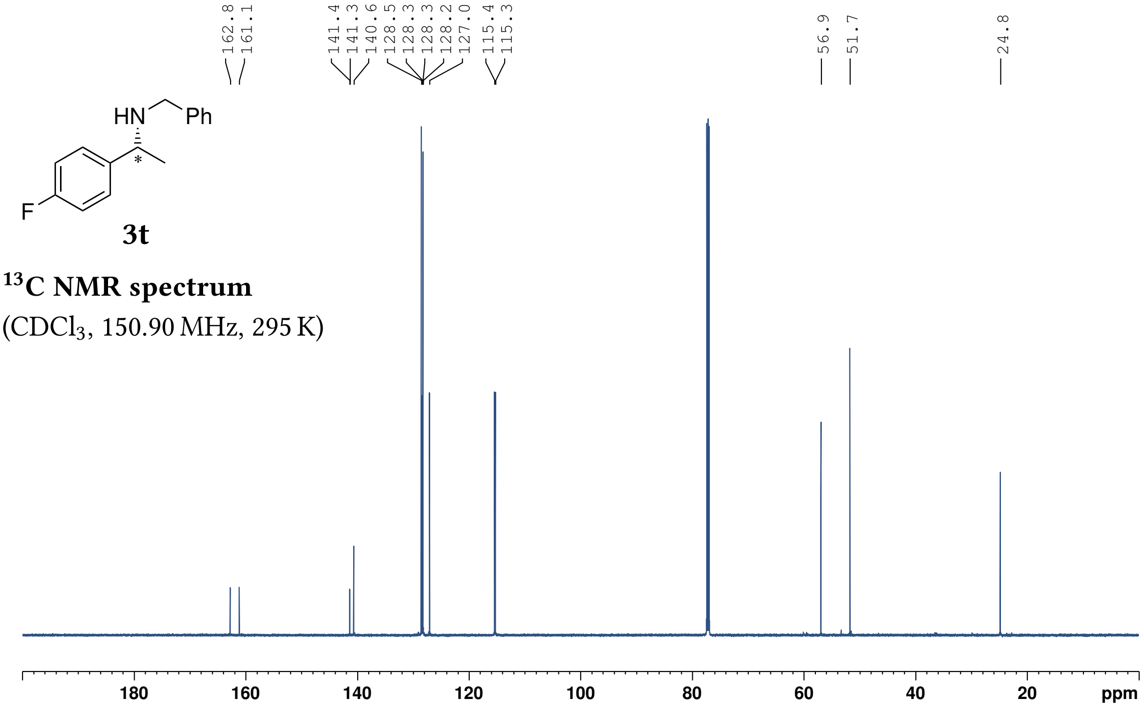

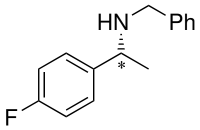

3t

^{19}F NMR spectrum
($CDCl_3$, 376.27 MHz, 295 K)

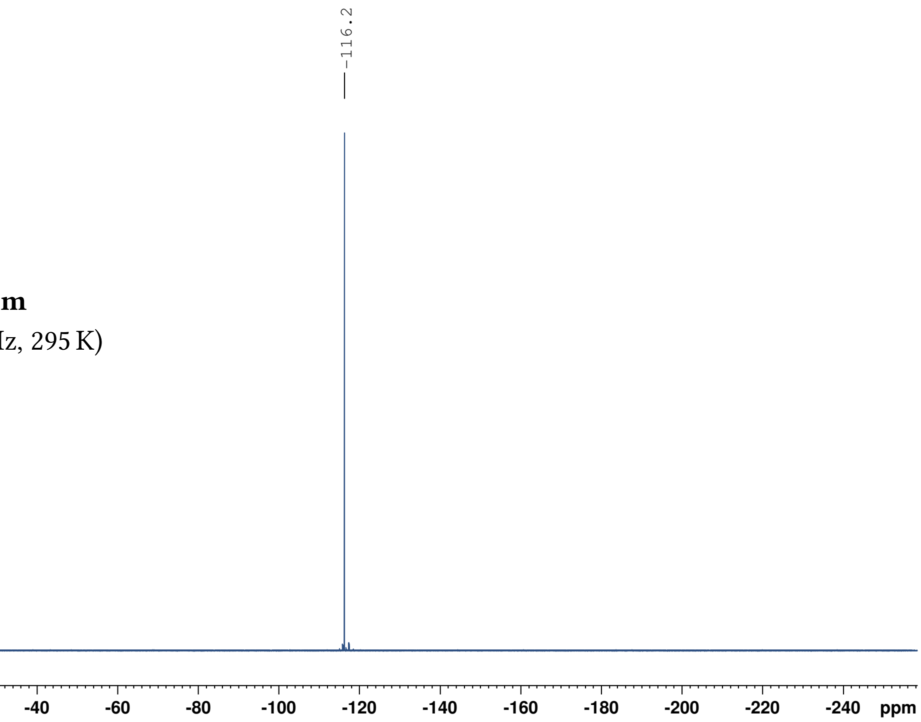

Compound 3u

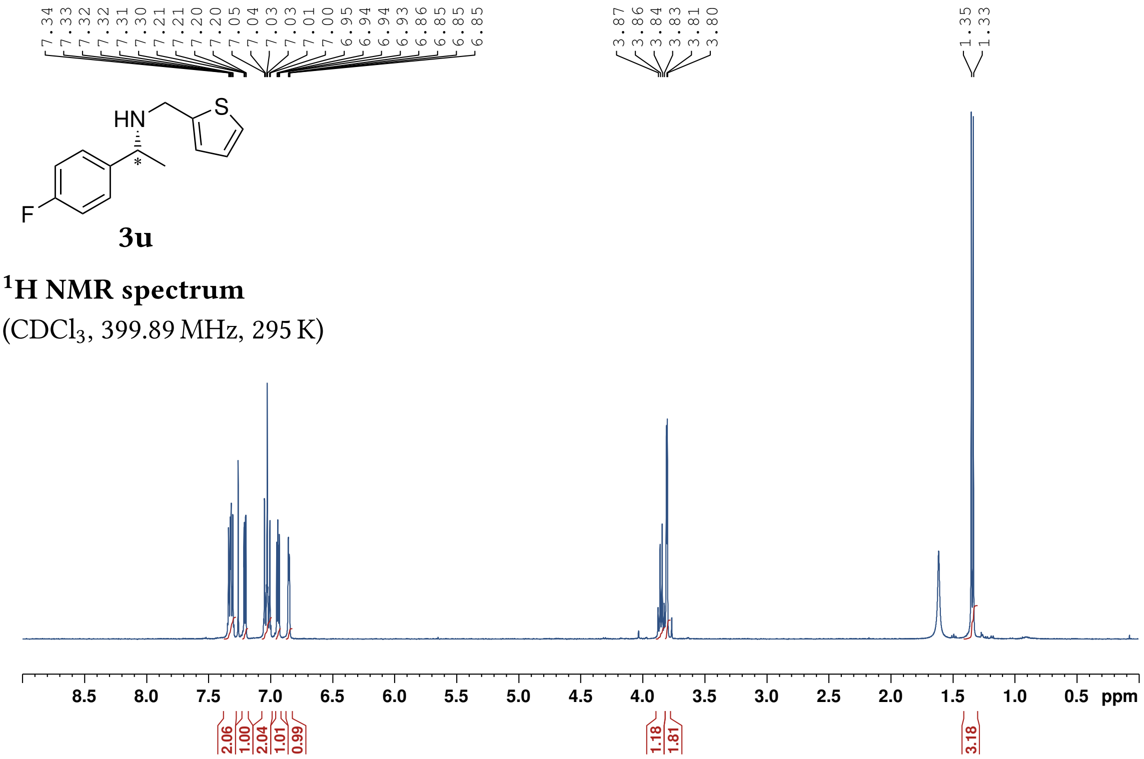

3u

^{1}H NMR spectrum
($CDCl_3$, 399.89 MHz, 295 K)

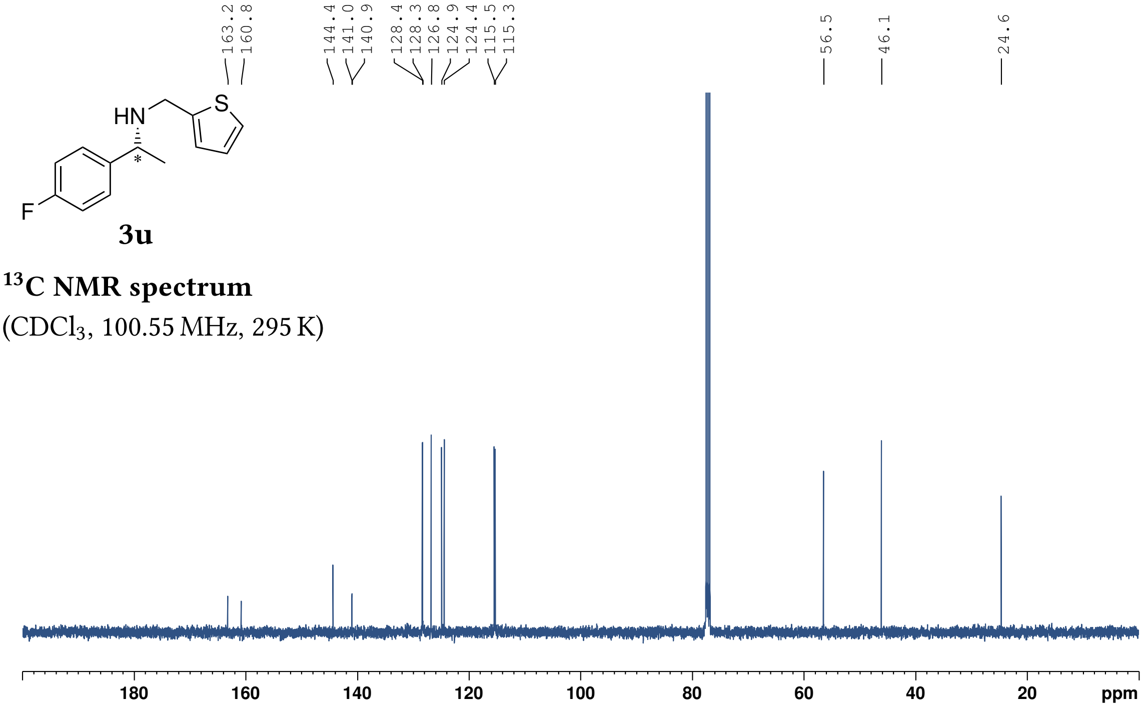

3u

^{13}C NMR spectrum
($CDCl_3$, 100.55 MHz, 295 K)

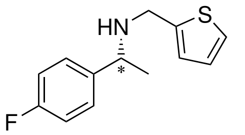

3u

^{19}F NMR spectrum
($CDCl_3$, 376.27 MHz, 295 K)

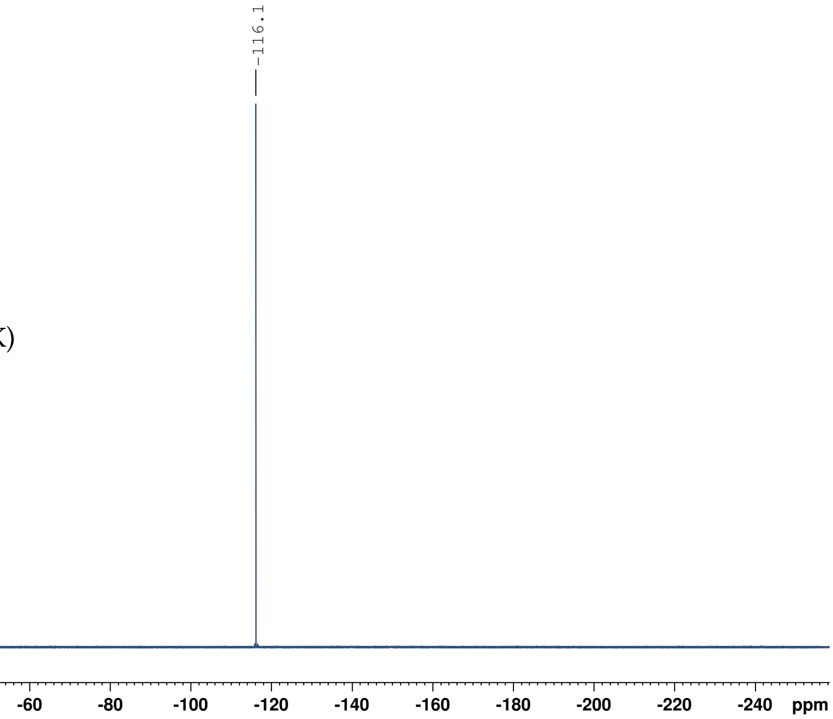

Compound 6

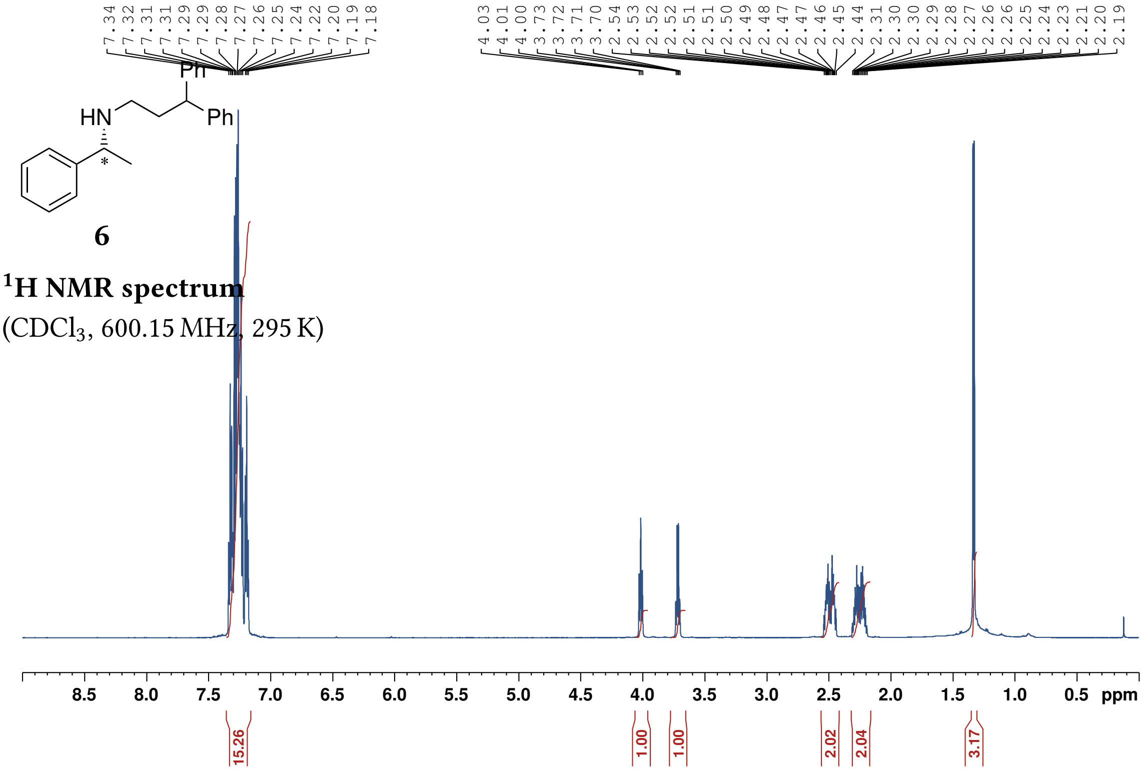

6

^{1}H NMR spectrum
($CDCl_3$, 600.15 MHz, 295 K)

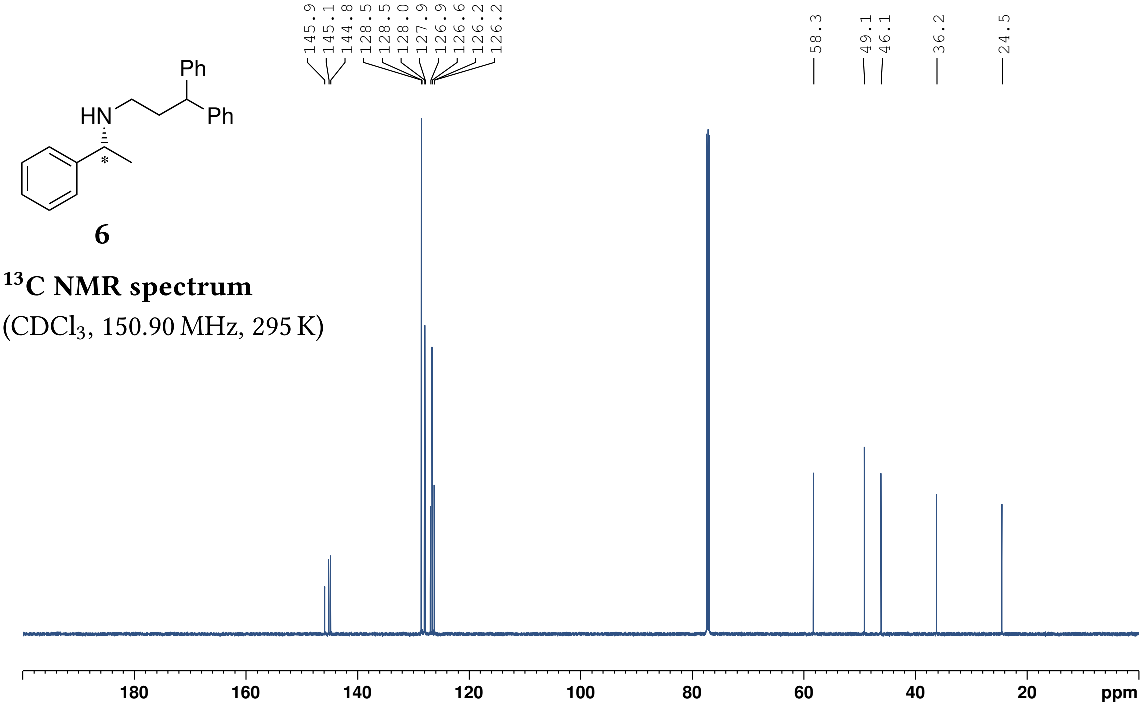

6

^{13}C NMR spectrum
($CDCl_3$, 150.90 MHz, 295 K)

Compound 8

8

^{1}H NMR spectrum
($CDCl_3$, 399.89 MHz, 295 K)

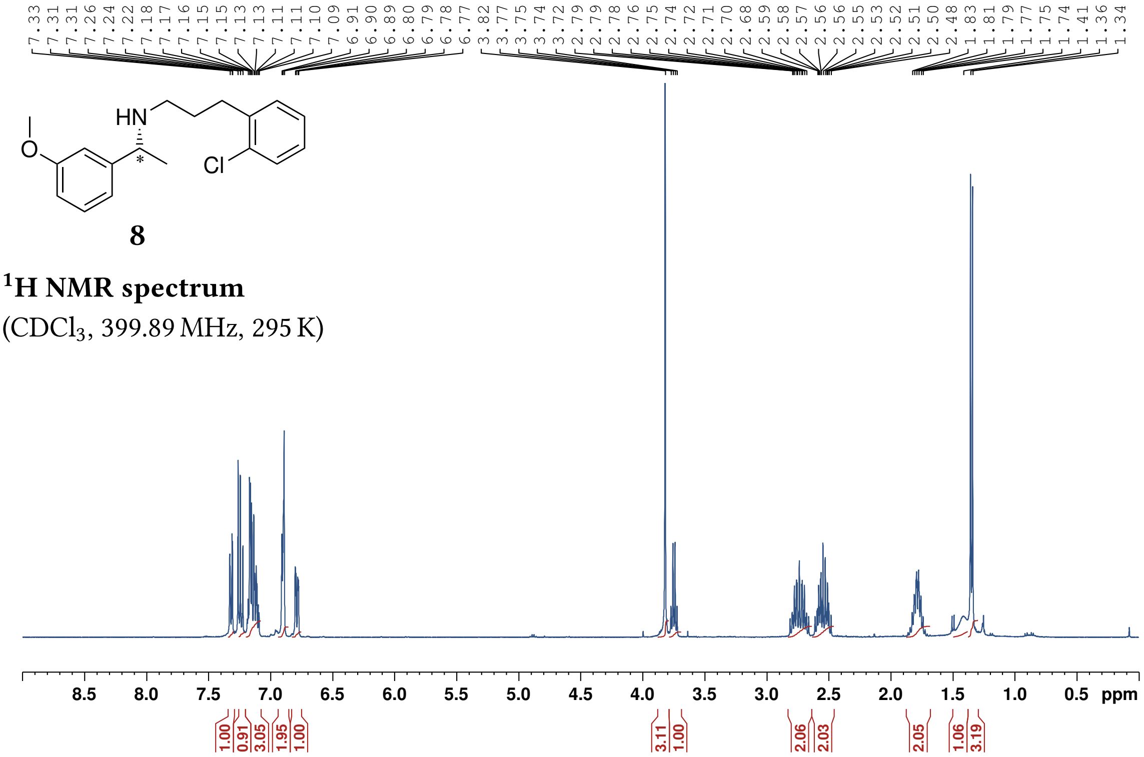

8

^{13}C NMR spectrum
($CDCl_3$, 100.55 MHz, 295 K)

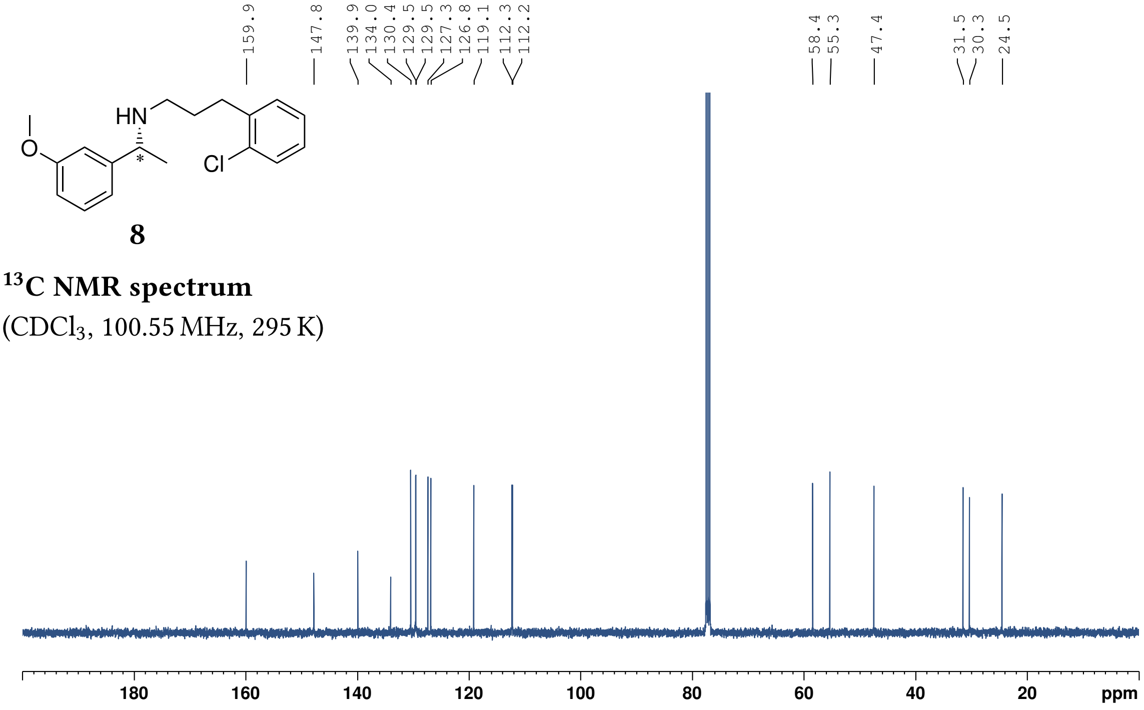

References

[1] W. L. F. Armarego, C. L. L. Chai, *Purification of laboratory chemicals*, Elsevier/Butterworth-Heinemann, Amsterdam, Boston, **2009**.

[2] H. E. Gottlieb, K. Vadim, A. Nudelman, *J. Org. Chem.* **1997**, *62*, 7512–7515.

[3] G. R. Fulmer, A. J. M. Miller, N. H. Sherden, H. E. Gottlieb, A. Nudelman, B. M. Stoltz, J. E. Bercaw, K. I. Goldberg, *Organometallics* **2010**, *29*, 2176–2179.

[4] C. K. Blasius, V. Vasilenko, L. H. Gade, *Angew. Chem. Int. Ed.* **2018**, *57*, 10231–10235.

[5] V. N. Wakchaure, P. S. J. Kaib, M. Leutzsch, B. List, *Angew. Chem. Int. Ed.* **2015**, *54*, 11852–11856.

[6] J. Clayden, M. Donnard, J. Lefranc, A. Minassi, D. J. Tetlow, *J. Am. Chem. Soc.* **2010**, *132*, 6624–6625.

[7] M. R. Adams, C. H. Tien, R. McDonald, A. W. Speed, *Angew. Chem. Int. Ed.* **2017**, *56*, 16660–16663.

[8] F. Chen, Z. Ding, J. Qin, T. Wang, Y. He, Q. H. Fan, *Org. Lett.* **2011**, *13*, 4348–4351.

[9] K. Gao, H. Yorimitsu, A. Osuka, *Angew. Chem. Int. Ed.* **2016**, *55*, 4573–4576.

[10] S. S. Al-Showiman, I. M. Al-Najjar, A. M. Al-Shalaan, *Spectrochim. Acta* **1987**, *43a*, 1055–1058.

[11] S. Al-Showiman, A. Al-Shalaan, I. Al-Najjar, *Arab Gulf J. Sci. Res.* **1987**, *A5*, 359–375.

[12] R. R. Prasad, S. E. Seidner, D. B. Cordes, M. M. Lozinska, D. M. Dawson, M. J. Thompson, T. Düren, K. K. Chakarova, M. Y. Mihaylov, K. I. Hadjiivanov, F. Hoffmann, A. M. Slawin, S. E. Ashbrook, M. L. Clarke, P. A. Wright, *J. Mater. Chem. A* **2019**, *7*, 5685–5701.

[13] L. Benmekhbi, F. Louafi, T. Roisnel, J. P. Hurvois, *J. Org. Chem.* **2016**, *81*, 6721–6739.

[14] J. Qin, Z. Zhou, T. Cui, M. Hemming, E. Meggers, *Chem. Sci.* **2019**, *10*, 3202–3207.

[15] C. Wang, X. Wu, L. Zhou, J. Sun, *Chem. Eur. J.* **2008**, *14*, 8789–8792.

[16] K. Han, Y. Kim, J. Park, M. J. Kim, *Tetrahedron Lett.* **2010**, *51*, 3536–3537.
